# Supplementary material for: How growers make decisions impacts plant disease control
Source: PLoS Comput Biol. 2022 Aug 22;18(8):e1010309. doi: 10.1371/journal.pcbi.1010309 (PMC9394827; doi:10.1371/journal.pcbi.1010309)
Supplement: S1 Text — We dissect the behaviour that drives the dynamics of the “strategy vs.” models, specifically the response to the change in the number of infected fields. Fig A: Probability of infection, responsiveness of growers, expected profits, and switching terms for the “strategy vs. population” model. A For the default parameterisation, the probability of infection increases as the number of infected growers increase (qN and qC), though the probability non-controllers will be infected via horizontal transmission (pN(Horiz)) falls as I increases. B The expected profits for controllers, non-controllers and the population for different proportions of controllers in the population (c). For the default parameters, PC < P for all values of I. C Switching probabilities for controllers and non-controllers for different c. As PC < P, controllers should always have a non-zero probability of switching strategy, whereas non-controllers should never start to control. D The responsiveness of growers (η) does not affect the final equilibrium values, though it does impact the time it takes to reach equilibrium. E shows the expected profits for controllers, non-controllers and the population when the cost of control ϕ, = 0.125. Now, at high levels of I, it is profitable to control. Where PC = PN, both strategies will be present at equilibrium. F For ϕ = 0.125, for low values of I controllers should switch strategy, but as I increases non-controllers should have a non-zero probability of switching into the clean seed system. For this parameter set, the equilibrium value of I is therefore 411, with 349 infected controllers and 63 infected non-controllers. (PDF) [file pcbi.1010309.s001.pdf]

1 S1: Underlying behaviour of "strategy vs." models.

1.1 Probability of infection, responsiveness, profits, and switching terms in the "strategy vs." models

To demonstrate the underlying drivers of dynamics in our models, we dissect the behaviour of the "strategy vs. population" model in some depth. We first investigated the response of the probability of infection, the expected profits and the probability of switching strategy to the total number of infected fields (FigA). Generally, as the number of infected fields increases, so too does the probability of infection for controllers and non-controllers (q_C and q_N respectively) (FigAA). However, the increased number of infected fields changes the relative importance of the infection pathways for non-controllers. With an increase in the number of infected fields, the instantaneous probability of horizontal transmission for non-controllers (p_N(Horiz), Equation 19 in the main text), decreases as field infection has already taken place through vertical transmission (p_N(Vert), Equation 18 in the main text) (FigAA).

For the default parameterisation (FigAB), the expected profits for the non-controllers (P_N) are always higher than that of the population, so they should always have a zero probability of switching strategy (FigAC). Conversely, P_C is always below the population average, and thus they should always have a non-zero probability of switching. This results in an equilibrium where there are no controllers present, as all of them have abandoned using the unprofitable CSS. However, by reducing the cost of clean seed ($\phi = 0.125$, equivalent to a 50% subsidy), at around $I = 411$ fields control becomes more profitable and non-controllers have a non-zero probability of switching strategy (FigAE). As at this point controllers cannot switch strategy, there is an "all control" equilibrium. The point at which $P_N = P_C$ (i.e. when $I \approx 411$, which is the total number of infected fields in the system and does not describe the number of infected controllers and non-controllers) leads to an equilibrium where both control strategies are present.

The value of η (the responsiveness of growers has no effect on the equilibrium attained by the

growers in the "strategy vs. population" models, as the equilibrium is based only on the relative values of P_C, P_N and P, though it did affect the time it took to reach equilibrium (FigAD). This also holds true for the "strategy vs. alternative" model.

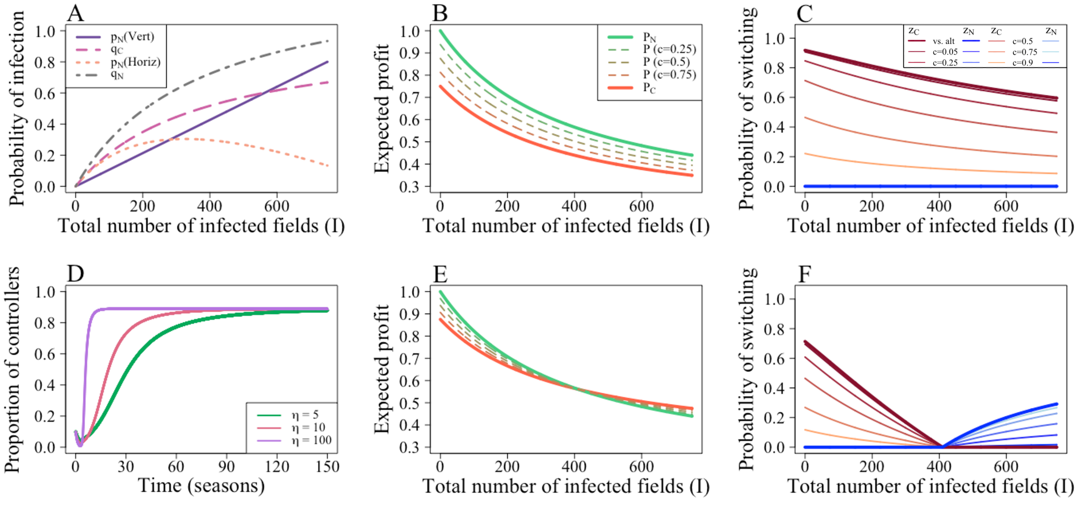

Fig A: Probability of infection, responsiveness of growers, expected profits, and switching terms for the "strategy vs. population" model. A For the default parameterisation, the probability of infection increases as the number of infected growers increase (q_N and q_C), though the probability non-controllers will be infected via horizontal transmission ($p_{N(\text{Horiz})}$) falls as I increases. B The expected profits for controllers, non-controllers and the population for different proportions of controllers in the population (c). For the default parameters, $P_C < P$ for all values of I. C Switching probabilities for controllers and non-controllers for different c. As $P_C < P$, controllers should always have a non-zero probability of switching strategy, whereas non-controllers should never start to control. D The responsiveness of growers (η) does not affect the final equilibrium values, though it does impact the time it takes to reach equilibrium. E shows the expected profits for controllers, non-controllers and the population when the cost of control $\phi, = 0.125$. Now, at high levels of I, it is profitable to control. Where $P_C = P_N$, both strategies will be present at equilibrium. F For $\phi = 0.125$, for low values of I controllers should switch strategy, but as I increases non-controllers should have a non-zero probability of switching into the clean seed system. For this parameter set, the equilibrium value of I is therefore 411, with 349 infected controllers and 63 infected non-controllers.
