# Supplementary material for: How growers make decisions impacts plant disease control
Source: PLoS Comput Biol. 2022 Aug 22;18(8):e1010309. doi: 10.1371/journal.pcbi.1010309 (PMC9394827; doi:10.1371/journal.pcbi.1010309)
Supplement: S2 Text — A comparison of the spatial-stochastic and deterministic models show good agreement between the predictions made by each model. Fig A: Comparison of default behaviour of the “grower vs. alternative” spatial-stochastic and deterministic models. A Dynamics for spatial model. As with the deterministic model C, under the default parameterisation no growers use the CSS after 10 seasons. Adding a subsidy in B and D allows for the two-strategy equilibrium. The figures show the mean for 100 runs of each model, and the error bars show one standard deviation. The equilibrium values and dynamics for spatial (A and C) and non-spatial models (B and D) are very similar in both cases, emphasised in E and F, which show the proportions controlling and infected for the spatial-stochastic (“stoch.”) and deterministic (“det.”) models. Fig B: Response to changes in the rate of horizontal transmission and cost of control for the “grower vs. alternative” models. A and C The proportion of controllers and infected fields after 50 seasons for the spatial-stochastic model and B and D the equilibrium values of control (SC + IC) and infection (IN + IC) for the deterministic model. Aside from the parameters being scanned over, the default parameters are used (Table 1 and Table A in S2 Text). The results in A and C closely align with the equilibrium values in the non-spatial model (B and D), indicating that our results are robust to spatial and stochastic effects. In A and C the means over 100 runs and the error bars show one standard deviation around the mean. Fig C: Effect of systematic misestimation in the spatial-stochastic model. A For the default parameters, as the perceptions of the dispersal scale for the whitefly vector (να) increase, fewer growers use the control scheme as they estimate that they would likely end up infected. B As perceptions of the rate of horizontal transmission increase (νβ), fewer growers use the CSS in a pattern that matches that seen in Fig 6A in the main text. Th [file pcbi.1010309.s002.pdf]

1 S2: Spatial-stochastic Model

As a supplementary question, we investigated whether the results we obtained with the "grower vs. alternative" assessment of profitability were robust to the addition of space and stochasticity.

1.1 Methods

1.1.1 Host landscape and initial conditions

Disease spreads on a 1,000 Ha square landscape containing $N = 750$ identically-sized fields (a similar density to [1]), placed at uniform random locations. All simulations are started with 75 growers using clean seed (i.e. 10% of the total population), distributed randomly across the landscape. A random selection of eight non-controllers is set to be infected initially ($\approx$ 1% total population). The location of the growers was kept constant between simulation runs, though the growers that were either initially infected or used the clean seed system differed.

1.1.2 Dispersal and force of infection

Since horizontal transmission is a consequence of the movement of whitefly, the probability that a particular infected field leads to infection of a susceptible field depends upon the distance d between them. We model this using an exponential dispersal kernel

$$K(d, \alpha) = \frac{1}{2\pi\alpha^2} \exp\left(-d/\alpha\right), \tag{1}$$

in which α is the dispersal scale [2, 3]. This sets the force of infection, Γ_k, upon the field belonging to grower k

$$\Gamma_k = \beta_S \sum_{\substack{l \neq k \\ l \text{ is infected}}} K(d_{k,l}, \alpha), \tag{2}$$

in which the sum runs over all infected fields (l), $d_{k,l}$ is the distance between fields k and l, and β_S is the effective rate of infection in the spatial version of our model (Table A in S2 Text). The

Table A: Summary of parameter values and initial conditions required for the spatial-stochastic model.

Parameter	Meaning	Value	Reference
$1/\gamma$	Length of the growing season	300 days	[1]; [4]
η	Responsiveness of growers	10	Assumed (see main text)
Y	Maximum yield	1	All values scaled relative to yield
L	Loss due to infection	0.6	[5]; [6]
ϕ	Cost of control	0.25	[1]
N	Total number of fields/growers	750	Illustrative
α	Dispersal scale parameter for whitefly	150 m	[1]; [10]
β_S	Effective rate of secondary infection (spatial)	110 day^{-1} field^{-1}	Calibrated to [1]
$S_C(0)$	Initial proportion of susceptible controllers	$0.1N$	Illustrative
$I_C(0)$	Initial proportion of infected controllers	0	Illustrative
$S_N(0)$	Initial proportion of susceptible non-controllers	$0.88.93N$	Illustrative
$I_N(0)$	Initial proportion of infected non-controllers	$0.0107N$	Illustrative

force of infection sets the rate at which grower k becomes infected; it varies in both time and space (since it depends not only on the instantaneous number of infected fields, but also on their spatial arrangement).

1.1.3 Model dynamics

Each individual grower is characterised by their current strategy as adopted on planting (i.e. controller or non-controller), as well as their current infection status (i.e. susceptible or infected). Between harvesting and replanting events, dynamics are independent of the strategy adopted. We simulate the model using Gillespie's algorithm [7].

The only event that can affect an infected grower (i.e. class I_C or I_N) is harvesting; this occurs at rate γ. As well as harvesting, susceptible growers (i.e class S_C or S_N) can also become horizontally infected. This occurs, for grower k, at rate Γ_k (Equation 2). As noted above, the force of infection not only depends on time, but also takes different values for each grower.

1.1.4 Replanting and grower behaviour

Replanting is assumed to occur immediately after harvesting, and potentially leads to changes in both strategy and infection status. Different growers make different assessments of the probability of horizontal infection next season depending on their instantaneous force of infection, with

$$\begin{aligned}\begin{array}{c}\text{Estimated probability of}\\ \text{horizontal infection for grower } k\end{array} &= \frac{\text{Force of infection on grower } k}{\text{Force of infection on grower } k + \text{Harvesting rate}}, \quad (3)\\ &= \frac{\Gamma_k}{\Gamma_k + \gamma}, \quad (4)\end{aligned}$$

We assume growers make decisions according to spatial analogues of Equations 17 and 20 in the main text:

$$\begin{aligned}q_C^k &= \text{Grower } k\text{'s estimate of the probability of infection next season if control is adopted}, \quad (5)\\ &= \frac{\Gamma_k}{\Gamma_k + \gamma}, \quad (6)\end{aligned}$$

and

$$\begin{aligned}q_N^k &= \text{Grower } k\text{'s estimate of the probability of infection next season if control is not adopted},\\ &= \frac{p(I_C + I_N)}{N} + \left(\frac{\Gamma_k}{\Gamma_k + \gamma}\right)\left(1 - \frac{p(I_C + I_N)}{N}\right). \quad (7)\end{aligned}$$

All other aspects of the behavioural model follow essentially unchanged, although the switching terms now enter the model via a single Bernoulli trial [8] at the time of planting to determine whether a grower switches strategy. Whether or not non-controllers become vertically infected is also simulated using a single Bernoulli trial at the time of planting (rather than via a systematic alteration to a rate parameter as in Equations 5-8 in the main text).

1.1.5 Misestimating parameters

We also investigated misestimation of epidemiological parameters in the spatial model, focusing on the dispersal scale (α) and and the rate of horizontal transmission, β_S:

$$q_\alpha = \nu_\alpha \alpha, \tag{8}$$

$$q_{\beta_S} = \nu_{\beta_S} \beta_S. \tag{9}$$

The exponential dispersal kernel ($\tilde{K}(d, q_\alpha)$) used in growers' decision making is now given by:

$$\tilde{K}(d, q_\alpha) = \frac{1}{2\pi q_\alpha^2} \exp\left(-d/q_\alpha\right), \tag{10}$$

which sets the estimated force of infection, $\tilde{\Gamma}_k$, upon the field belonging to grower k via

$$\tilde{\Gamma}_k = q_{\beta_S} \sum_{\substack{l \neq k \\ l \text{ is infected}}} \tilde{K}(d_{k,l}, q_\alpha). \tag{11}$$

1.1.6 Parameterisation

The majority of parameters in the spatial model took the same values to those in the simpler non-spatial model that was introduced in the main text (Table 1 in the main text). However, following [1] and based on dispersal of *B. tabaci* reported by [9] and [10], we set the scale parameter of our dispersal kernel to be $\alpha = 150$ m. The move to a spatially-explicit model required a re-scaling of the rate of secondary infection, which we set to $\beta = 110$ day^{-1} field^{-1}. This value was again chosen to ensure that, after 10 seasons, on average 50% of fields would be infected.

1.2 Results

Under the default parameterisation, no grower controls (Fig AA), though providing a 50% subsidy encouraged participation in the CSS (Fig AB). Disease spreads rapidly between fields, both due to the high values of β and ϕ (which discourage control) and the non-spatial aspect of trade, which allows disease to spread far away from its initial source.

The results of our spatial model are comparable with the deterministic version of the model (Fig B). For equivalent rates of horizontal transmission, the equilibrium values for the deterministic model are similar to the values attained after 50 seasons in the spatial-stochastic model (Fig B A-B). Disease extinction in the stochastic model was rare; it occurred in every simulation when $\beta_S = 0$ day^{-1} field^{-1}, and around 40% of the time when $\beta_S = 40$ day^{-1} field^{-1}. Above $\beta_S = 40$ day^{-1} field^{-1}, disease extinction was uncommon.

As in Fig 6 of the main text, which shows the analogous result for the non-spatial deterministic model, the kinks in these graphs are a result of susceptible controllers (S_C) growers ceasing to switch strategy (around $\beta_s = 50$ day^{-1} field^{-1} and $\beta = 0.003$ day^{-1} in Fig BA-B). As the infection pressure is increasing, these susceptible growers then become infected and switch into the non-control strategy, causing an overall decrease in controllers.

In both cases, when ϕ is varied, at around $\phi = 0.19$, growers should stop using the control strategy (Fig BC-D).

1.2.1 Effect of systematic uncertainty

Growers misestimating the dispersal scale for the whitefly vector (α) has a similar effect to when growers misestimated β in the deterministic model. As the perceived value of α (q_α, given by $q_\alpha = \nu_\alpha \alpha$, Table A) increases, fewer growers participate in the CSS as they estimated that they would be paying the dual penalty of the cost of control and loss due to disease (Fig C).

When growers misestimate the rate of horizontal transmission, β_S, the pattern of CSS use again resembles that of the deterministic model (Fig 6A). At low values of the perceived value of β (q_{β_S},

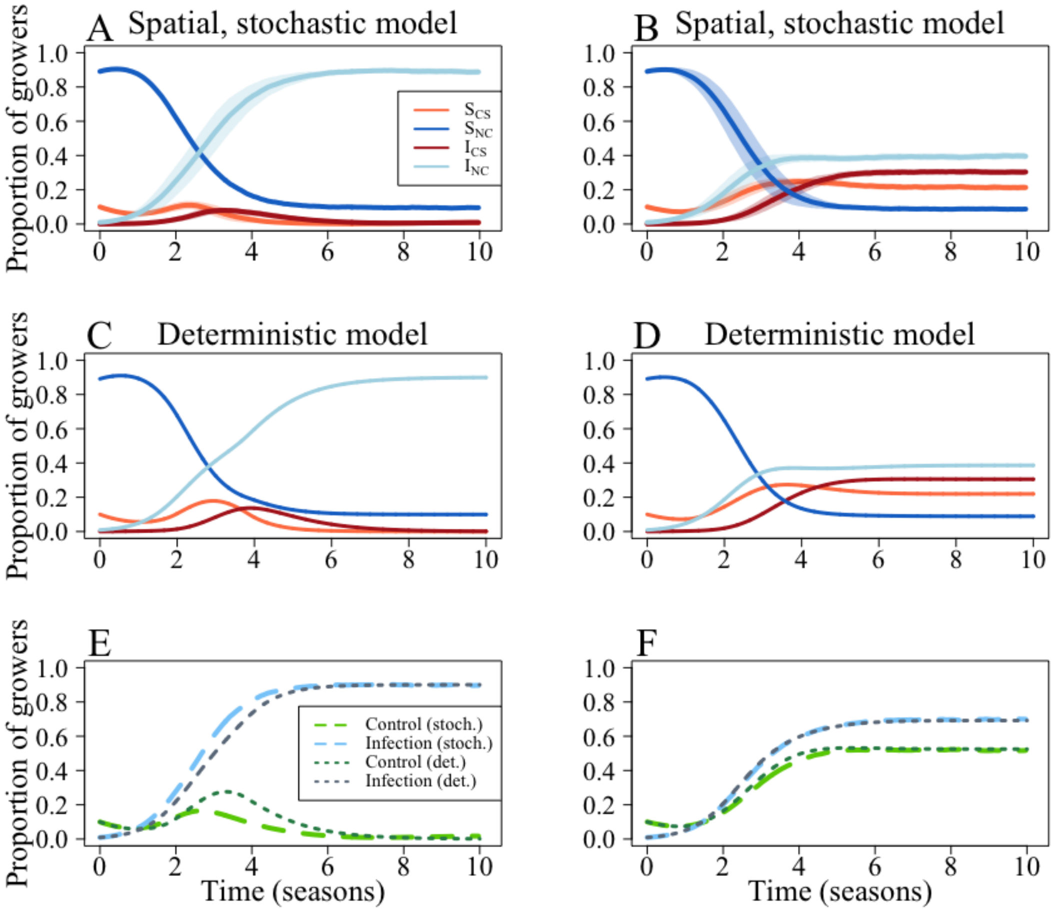

Fig A: Comparison of default behaviour of the "grower vs. alternative" spatial-stochastic and deterministic models. A Dynamics for spatial model. As with the deterministic model C, under the default parameterisation no growers use the CSS after 10 seasons. Adding a subsidy in B and D allows for the two-strategy equilibrium. The figures show the mean for 100 runs of each model, and the error bars show one standard deviation. The equilibrium values and dynamics for spatial (A and C) and non-spatial models (B and D) are very similar in both cases, emphasised in E and F, which show the proportions controlling and infected for the spatial-stochastic ("stoch.") and deterministic ("det.") models.

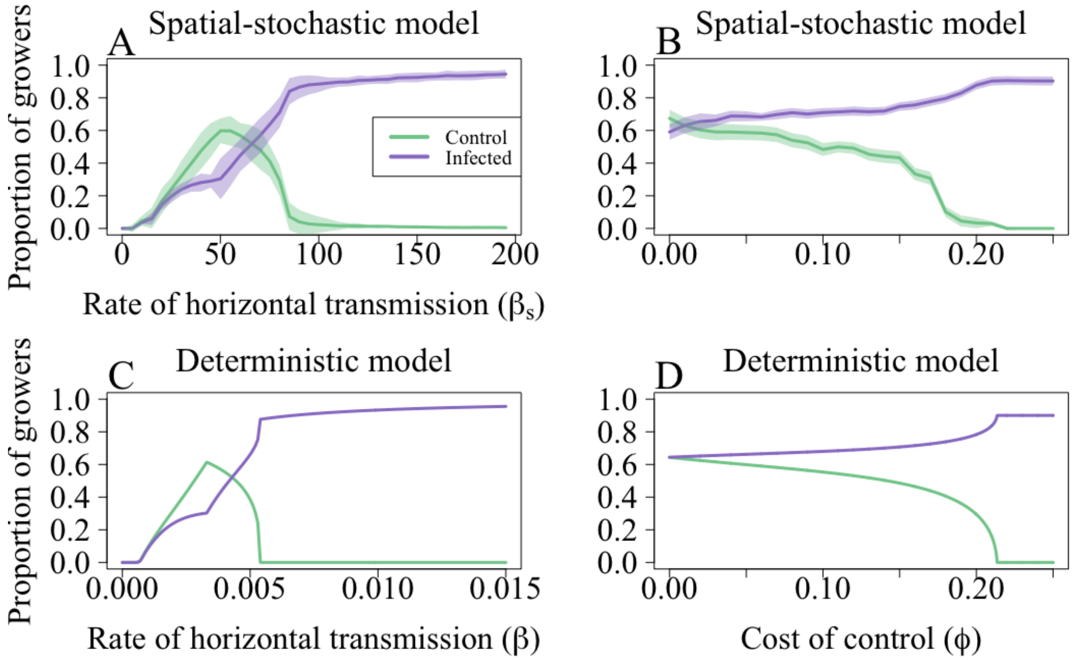

Fig B: Response to changes in the rate of horizontal transmission and cost of control for the "grower vs. alternative" models. A and C The proportion of controllers and infected fields after 50 seasons for the spatial-stochastic model and B and D the equilibrium values of control ($S_C + I_C$) and infection ($I_N + I_C$) for the deterministic model. Aside from the parameters being scanned over, the default parameters are used (Table 1 in the main text and Table A in S2 Text). The results in A and C closely align with the equilibrium values in the non-spatial model (B and D), indicating that our results are robust to spatial and stochastic effects. In A and C the means over 100 runs and the error bars show one standard deviation around the mean.

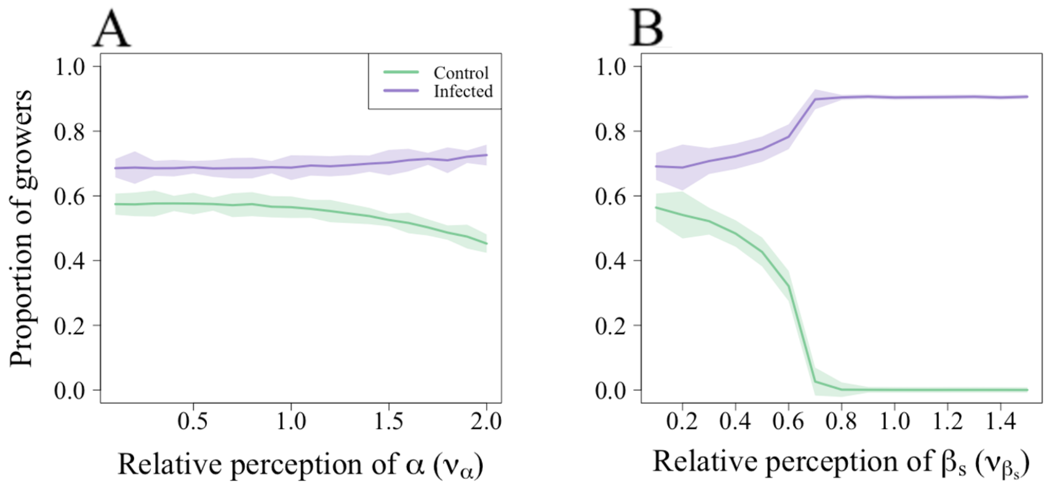

Fig C: Effect of systematic misestimation in the spatial-stochastic model. A For the default parameters, as the perceptions of the dispersal scale for the whitefly vector (ν_α) increase, fewer growers use the control scheme as they estimate that they would likely end up infected. B As perceptions of the rate of horizontal transmission increase (ν_β), fewer growers use the CSS in a pattern that matches that seen in Fig 6A in the main text. The mean values were calculated over 100 runs are the error bars show one standard deviation around the mean.

with $q_{\beta_S} = \nu_{\beta_S}\beta_S$, Table A), more growers use the control scheme as they believe that they are unlikely to be infected. As q_{β_S} increases, growers believe that they are likely to pay the dual penalty of the cost of control, ϕ, and the loss due to infection, L, and therefore abandon the CSS.

1.2.2 Spatial spread of disease.

Fig D shows the spatial component of disease spread in the spatial-stochastic model, restricting attention to the case when there is only horizontal transmission ($p = 0$). Disease then spreads in a much more "wavelike" pattern, as expected from the thin-tailed exponential dispersal kernel as adopted for whitefly. As there was no vertical transmission, we also removed "control" from the strategy set of the growers, as there was no benefit to controlling for disease and thus the strategy would quickly disappear from the population. Unlike the other models, we also start our epidemic with a cluster of infected fields. Making these changes allows us to focus on the underlying dynamics of the model.

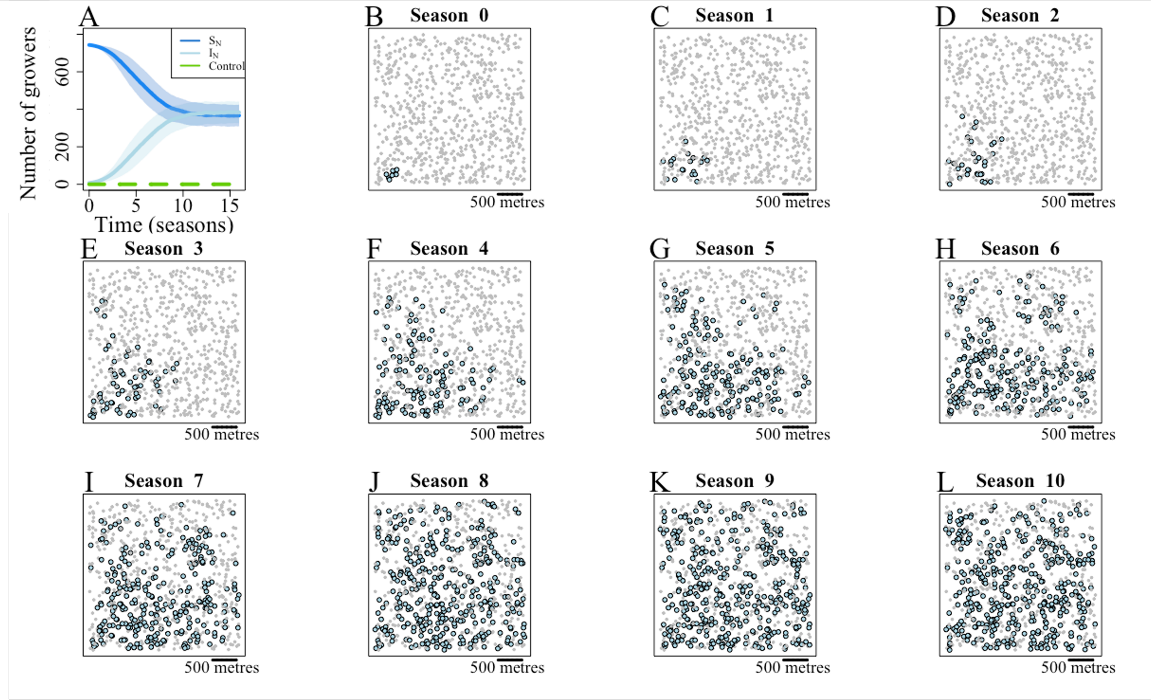

Fig D: Spatial spread of infection in spatial-stochastic model. To emphasise the spatial component of infection, vertical transmission has been removed from the model (i.e. $p = 0$). Additionally, we have removed the "control" strategy from the growers as, without vertical transmission, this would have disappeared from the population within 5 seasons. The light blue dots show infected fields, whilst the grey dots are uninfected fields.

1.3 Discussion

The results for this model were comparable with those for the equivalent deterministic model (Fig B), demonstrating the robustness of our results to stochastic and spatial effects. This was in part due to the non-spatial aspect of trade, as it allowed for transmission across the relatively small landscape considered here [11]. When trade was not included (i.e. $p = 0$) the disease spread across the landscape in a more "wavelike" pattern as expected from the thin-tailed dispersal kernel for the whitefly [12] (Fig D). However, when trade occurs via a market or central organisation the majority of transactions occur over a scale larger than our landscape (a square with sides 3.16 km), with around 70 % of transactions occur over a scale of 10-50 km [13]. For more informal trade settings, with growers interacting with each other, a proximity-based kernel may be more appropriate [11], [1] (though both of these were modelled over a larger landscape than ours). However, such exchanges are hard to parameterise as the probability of exchanging planting material is highly variable across settings (varying between 30 - 92.51% over unspecified spatial scales; [14], [15], [16], [17] and [18]).

References

[1] McQuaid CF, Gilligan CA, van den Bosch F. Considering behaviour to ensure the success of a disease control strategy. Royal Society Open Science. 2017a;4(12):170721. doi:10.1098/rsos.170721.

[2] Cunniffe NJ, Gilligan CA. Use of Mathematical Models to Predict Epidemics and to Optimize Disease Detection and Management. In: Ristaino JB, Records A, editors. Emerging Plant Diseases and Global Food Security. APS Press; 2020.

[3] Fabre F, Coville J, Cunniffe NJ. Optimising reactive disease management using spatially explicit models at the landscape scale. In: Scott P, Strange R, Korsten L, Gullino ML, editors. Plant Diseases and Food Security in the 21st Century:. Springer; 2021.

[4] Jeger MJ, Holt J, van den Bosch F, Madden LV. Epidemiology of insect-transmitted plant viruses: modelling disease dynamics and control interventions. Physiological Entomology. 2004;29:291–304.

[5] Hillocks RJ, Raya MD, Mtunda K, Kiozia H. Effects of Brown Streak Virus Disease on Yield and Quality of Cassava in Tanzania. Journal of Phytopathology. 2001;149(7-8):389–394. doi:https://doi.org/10.1111/j.1439-0434.2001.tb03868.x.

[6] Ephraim N, Yona B, Evans A, Sharon A, Titus A. Effect of cassava brown streak disease (CBSD) on cassava (Manihot esculenta Crantz) root storage components, starch quantities and starch quality properties. International Journal of Plant Physiology and Biochemistry. 2015;doi:DOI:10.5897/IJPPB2015.0227.

[7] Keeling MJ, Rohani P. Modeling Infectious Diseases in Humans and Animals. Princeton University Press; 2008. Available from: http://www.jstor.org/stable/j.ctvcm4gk0.

[8] Grimmett G, Welsh D. Probability: an introduction. Oxford University Press; 2014.

[9] Byrne DN, Rathman RJ, Orum TV, Palumbo JC. Localized migration and dispersal by the sweet potato whitefly, Bemisia tabaci. Oecologia. 1996;105:320–328.

[10] Byrne DN. Migration and dispersal by the sweet potato whitefly, Bemisia tabaci. Agricultural and Forest Meteorology. 1999;97(4):309–316. doi:https://doi.org/10.1016/S0168-1923(99)00074-X.

[11] McQuaid CF, van den Bosch F, Szyniszewska A, Alicai T, Pariyo A, Chikoti PC, et al. Spatial dynamics and control of a crop pathogen with mixed-mode transmission. PLOS Computational Biology. 2017b;13(7):1–18. doi:10.1371/journal.pcbi.1005654.

[12] Shaw MW. Simulation of population expansion and spatial pattern when individual dispersal distributions do not decline exponentially with distance. Proceedings of the Royal Society of London Series B: Biological Sciences. 1995;259(1356):243–248. doi:10.1098/rspb.1995.0036.

[13] Szyniszewska AM, Chikoti PC, Tembo M, Mulenga R, Gilligan CA, van den Bosch F, et al. Cassava planting material movement and grower behaviour in Zambia: implications for disease management. bioRxiv. 2019;doi:10.1101/528851.

[14] Ntawuruhunga P, Legg J, Okidi J, Okao-Okuja G, Tadu G, Remington T. Southern Sudan, Equatoria Region, Cassava Baseline Survey Technical Report. IITA. 2007;.

[15] Chikoti P, Melis R, Shanahan P. Farmer's Perception of Cassava Mosaic Disease, Preferences and Constraints in Lupaula Province of Zambia. American Journal of Plant Sciences. 2016; p. 1129–1138. doi:10.4236/ajps.2016.77108.

[16] Djaha KE, Abo K, Kone T, Kone D, Kone M. Analysis of the population structure of cassava growers, production systems, and plots' sanitary state in Côte d'Ivoire. Journal of Animal & Plant Sciences. 2018; p. 5833–5843.

[17] Houngue JA, Pita JS, Cacaï GHT, Zandjanakou-Tachin M, Abidjo EAE, Ahanhanzo C. Survey of farmers' knowledge of cassava mosaic disease and their preferences for cassava cultivars in three agro-ecological zones in Benin. Journal of Ethnobiology Ethnomedicine. 2018;doi:https://doi.org/10.1186/s13002-018-0228-5.

[18] Teeken B, Olaosebikan O, Haleegoah JAS, Oladejo E, Madu TU, Bello A, et al. Cassava Trait Preferences of Men and Women Farmers in Nigeria: Implications for Breeding. Economic Botany. 2018;72:263 – 277.
