# Supplementary material for: How growers make decisions impacts plant disease control
Source: PLoS Comput Biol. 2022 Aug 22;18(8):e1010309. doi: 10.1371/journal.pcbi.1010309 (PMC9394827; doi:10.1371/journal.pcbi.1010309)
Supplement: S3 Text — The “strategy vs.” models allowed more detailed mathematical analysis, whilst the stability of equilibria in the “grower vs.” models had to be determined numerically. Fig A: Possible equilibria for the “grower vs. population” model when p = 1. With the higher probability of vertical transmission, the “no control” equilibrium is possible for the “grower vs. population” model as there will be no non-infected, non-controlling (SN) growers at equilibrium (Eq 56). However, now that p = 1, there can never be a disease-free equilibrium for this parameter set (as R0 > 1). Table A: Range of values used for parameters when evaluating the stability of the “grower vs.” models. (PDF) [file pcbi.1010309.s003.pdf]

1 S3: Mathematical derivations

1.1 The basic reproductive number (R_0) for the "strategy vs." models.

To find the value for R_0, we calculated the next-generation matrix (NGM; [1]). This relies on the decomposing a linearised version of the model into two matrices: the first contains the terms relating to disease transmission (matrix J_F) whilst the second has terms relating to non-epidemiological transitions between states (matrix J_V). The NGM, K, is given by $K = J_F J_V^{-1}$ [2]. To shorten the notation in what follows, we introduce the following function of state variables:

$$\Gamma = S_C z_{SC} + I_C z_{IC} + S_N(1 - z_{SN}) + I_N(1 - z_{IN}). \tag{1}$$

We focus only on the infected compartments in the general model, leading to:

$$\frac{dI_C}{dt} = \beta S_C(I_C + I_N) - \gamma I_C, \tag{2}$$

$$\frac{dI_N}{dt} = \gamma\Gamma\left(\frac{p(I_C + I_N)}{N}\right) + \beta S_N(I_C + I_N) - \gamma I_N. \tag{3}$$

The disease-free equilibrium (DFE) is given by $(S_C, I_C, S_N, I_N) = (0, 0, N, 0)$. Given that we have restricted the model to just the infected compartments, the DFE can also be written as $(I_C, I_N) = (0, 0)$

The matrix of rates at which new infections occur is

$$F = \begin{bmatrix} \beta S_C(I_C + I_N) \\ \gamma\Gamma\left(\frac{p(I_C+I_N)}{N}\right) + \beta S_N(I_C + I_N) \end{bmatrix}. \tag{4}$$

The matrix of rates at which infections are removed is

$$V = \begin{bmatrix} \gamma I_C \\ \gamma I_N \end{bmatrix}. \tag{5}$$

The Jacobians for these matrices are:

$$J_F = \begin{bmatrix} \beta S_C & \beta S_C \\ \gamma\Gamma\left(\frac{p}{N}\right) + \gamma\frac{\partial\Gamma}{\partial I_C}\left(\frac{p(I_C+I_N)}{N}\right) + \beta S_N & \gamma\Gamma\left(\frac{p}{N}\right) + \gamma\frac{\partial\Gamma}{\partial I_N}\left(\frac{p(I_C+I_N)}{N}\right) + \beta S_N \end{bmatrix}, \tag{6}$$

and

$$J_V = \begin{bmatrix} \gamma & 0 \\ 0 & \gamma \end{bmatrix}. \tag{7}$$

We need to evaluate the Jacobians at the DFE. Note that $\gamma\Gamma$ is the net rate at which fields in which there is no control are planted, and so at the DFE, $\Gamma = N$. Note too that, since both are multiplied by $\frac{p(I_C+I_N)}{N}$ (which is zero at the DFE), the partial derivatives and $\frac{\partial\Gamma}{\partial I_C}$ and $\frac{\partial\Gamma}{\partial I_N}$ do not need to be calculated.

At the DFE, the J_F becomes

$$J_F = \begin{bmatrix} 0 & 0 \\ \gamma p + \beta N & \gamma p + \beta N \end{bmatrix}, \tag{8}$$

and J_V^{-1} is given by:

$$J_V^{-1} = \begin{bmatrix} 1/\gamma & 0 \\ 0 & 1/\gamma \end{bmatrix}. \tag{9}$$

The NGM, $K = J_F J_V^{-1}$, is therefore given by:

$$\begin{bmatrix} 0 & 0 \\ p + \frac{\beta N}{\gamma} & p + \frac{\beta N}{\gamma} \end{bmatrix} \tag{10}$$

The dominant eigenvalue for this matrix is $\lambda_1 = \frac{\beta N}{\gamma} + p$, which gives the R_0 for the system. This can be further broken down into distinct components corresponding to horizontal ($R_0^H = \frac{\beta N}{\gamma}$) and

vertical ($R_0^V = p$) transmission [3].

1.2 Stability of equilibria in the "strategy vs. population" model.

There are four equilibria, which can be distinguished by the presence of disease and the proportion of growers controlling at equilibrium.

- **Disease-free equilibrium** at which the disease is not able to spread even when there is no control via clean seed

$$(S_C, I_C, S_N, I_N) = (0, 0, N, 0). \tag{11}$$

- **Control-free, disease-endemic equilibrium** at which the disease is able to spread in the absence of control, but nevertheless no grower controls

$$(S_C, I_C, S_N, I_N) = \left(0, 0, \frac{\gamma(1-p)}{\beta}, \frac{\beta N - \gamma(1-p)}{\beta}\right). \tag{12}$$

- **All-control, disease-endemic equilibrium** at which all growers control, but nevertheless disease is still present in the system

$$(S_C, I_C, S_N, I_N) = \left(\frac{\gamma}{\beta}, \frac{\beta N - \gamma}{\beta}, 0, 0\right) \tag{13}$$

- **Two-strategy, disease-endemic equilibrium** at which both disease and control equilibrate at some intermediate level, with

$$S_C = \frac{N(R_0(\gamma pL - \beta\phi N) - \gamma pL)}{\gamma p^2 L}, \tag{14}$$

$$I_C = \frac{\beta\phi N^2(R_0(\beta\phi N - \gamma pL) + \gamma pL)}{\gamma p^2 L(\beta\phi N - \gamma pL)}, \tag{15}$$

$$S_N = \frac{N(\phi R_0 - pL)(\beta N(\beta\phi N - \gamma pL) + \gamma^2 pL)}{\gamma p^2 L(\beta\phi N - \gamma pL)}, \tag{16}$$

$$I_N = \frac{-\phi N R_0(\beta N(\beta\phi N - \gamma Lp) + \gamma^2 Lp)}{\gamma p^2 L(\beta\phi N - \gamma Lp)}. \tag{17}$$

We also note that none of Equations 11, 12, 12 or 14 - 17 have a dependence on the responsiveness of growers, η. Thus, the equilibrium attained is independent of this parameter, though it does affect the dynamics approaching equilibrium (FigAD in 1 Text).

The stability of each of these equilibria will be discussed in turn.

1.2.1 Disease-free equilibrium.

We determined the conditions for stability of each equilibrium in the "strategy vs." models by first evaluating the Jacobian matrix for the system at each possible equilibrium and then determining the eigenvalues for the matrix. The system can be reduced to three state variables, as $N = S_C + I_C + S_N + I_N$. The therefore becomes:

$$\frac{dS_C}{dt} = \gamma\left(S_C(1 - z_{SC}) + I_C(1 - z_{IC}) + (N - S_C - I_C)z_{IN}\right) - \beta S_C\left(I_C + I_N\right) - \gamma S_C, \tag{18}$$

$$\frac{dI_C}{dt} = \beta S_C\left(I_C + I_N\right) - \gamma I_C, \tag{19}$$

$$\frac{dI_N}{dt} = \gamma\left(S_C z_{SC} + I_C z_{IC} + (N - S_C - I_C)(1 - z_{IN})\right)\left(\frac{p(I_C + I_N)}{N}\right) + \beta(N - S_C - I_C - I_N)\left(I_C + I_N\right) - \gamma I_N. \tag{20}$$

where the switching terms are given by:

$$z_{SC} = z_{IC} = \max\left(0, 1 - e^{-\eta(P-P_C)}\right), \tag{21}$$

$$z_{SN} = z_{IN} = \max\left(0, 1 - e^{-\eta(P-P_N)}\right). \tag{22}$$

The DFE is given by $(S_C, I_C, I_N) = (0, 0, 0)$. The Jacobian matrix evaluated with these values is given by:

$$\begin{bmatrix} -\gamma(1-\exp(-\eta\phi)+\eta\phi) & \gamma(\exp(-\eta\phi)-\eta\phi) & 0 \\ 0 & -\gamma & 0 \\ 0 & \beta N + \gamma p & \beta N + \gamma p - \gamma \end{bmatrix}$$

From this, we can see the first eigenvalue is $\beta N - \gamma + \gamma p$. The 2×2 matrix that remains is given by:

$$\begin{bmatrix} -\gamma(1-\exp(-\eta\phi)+\eta\phi) & \gamma(\exp(-\eta\phi)-\eta\phi) \\ 0 & -\gamma \end{bmatrix}$$

This matrix is upper triangular, so the eigenvalues are given by the diagonal elements. We can see that $-\gamma$ and $-\gamma(1-\exp(-\eta\phi)+\phi)$ are the remaining eigenvalues for the matrix.

As $-\gamma$ and $-\gamma(1-\exp(-\eta\phi)+\phi)$ are always negative, the stability of this equilibrium is dependent upon $\beta N - \gamma + \gamma p < 0$. This corresponds to the R_0 found using the NGM.

1.2.2 Disease-endemic, all control equilibrium.

The disease-endemic, all control equilibrium is given by: $(S_C, I_C, I_N) = \left(\frac{\gamma}{\beta}, \frac{\beta N-\gamma}{\beta}, 0\right)$. As there are no non-controllers at this equilibrium, $P_C = P$ (Equation 23 in the main text).

The Jacobian matrix evaluated using these values is given by:

$$\begin{bmatrix} \frac{\partial \dot{S_C}}{\partial S_C} & \frac{\partial \dot{S_C}}{\partial I_C} & -\gamma \\ \beta N - \gamma & 0 & \gamma \\ \frac{\partial \dot{I_N}}{\partial S_C} & \frac{\partial \dot{I_N}}{\partial I_C} & -\gamma \end{bmatrix} \tag{23}$$

with:

$$\frac{\partial \dot{S_C}}{\partial S_C} = \gamma \left(\exp\left(\frac{-\eta(\beta N(\gamma pL - \beta\phi N) - \gamma^2 pL)}{\beta^2 N^2} \right) \right) - \frac{-\eta(\beta N(\gamma pL - \beta\phi N) - \gamma^2 pL)}{\beta^2 N^2} - \beta N \tag{24}$$

$$\frac{\partial \dot{S_C}}{\partial I_C} = -\gamma \left(1 - \exp\left(\frac{-\eta(\beta N(\gamma pL - \beta\phi N) - \gamma^2 pL)}{\beta^2 N^2} \right) \right) - \frac{-\eta(\beta N(\gamma pL - \beta\phi N) - \gamma^2 pL)}{\beta^2 N^2} \tag{25}$$

$$\frac{\partial \dot{I_N}}{\partial S_C} = \beta N + \gamma - \frac{\gamma p(\beta N - \gamma)}{\beta N} \left(\exp\left(\frac{-\eta(\beta N(\gamma pL - \beta\phi N) - \gamma^2 pL)}{\beta^2 N^2} \right) - \frac{-\eta(\beta N(\gamma pL - \beta\phi N) - \gamma^2 pL)}{\beta^2 N^2} \right) \tag{26}$$

$$\frac{\partial \dot{I_N}}{\partial I_C} = \frac{\partial \dot{I_N}}{\partial S_C}. \tag{27}$$

We can see that

$$\frac{\partial \dot{S_C}}{\partial S_C} = \frac{\partial \dot{S_C}}{\partial I_C} - \gamma + \beta N. \tag{28}$$

The characteristic equation for Matrix 23 is given by

$$-(\lambda + \gamma) \left(\lambda^2 - \lambda \left(\frac{\partial \dot{S_C}}{\partial I_C} - \gamma + \beta N \right) - (\beta N - \gamma) \left(\frac{\partial \dot{S_C}}{\partial I_C} \right) \right) \tag{29}$$

Solving this equation, we find the eigenvalues to be $-\gamma$, $\beta N - \gamma$ and

$$\frac{\partial \dot{S_C}}{\partial I_C} = -\gamma \left(1 - \exp\left(\frac{-\eta(N\beta(\gamma pL - \beta\phi N) - \gamma^2 pL)}{\beta^2 N^2} \right) + \frac{\eta(\beta N(\gamma pL - \beta\phi N) - \gamma^2 pL)}{\beta^2 N^2} \right). \tag{30}$$

As $-\gamma$ is always negative, the stability depends on the remaining eigenvalues. The second

eigenvalue is always negative for $\frac{\beta N}{\gamma} > 1$, which is also the R_0 for horizontal transmission (R_0^H). Given that this expression can be re-written as:

$$\frac{\partial \dot{S_C}}{\partial I_C} = -\gamma\left(1 - \exp\left(-x\right) + x\right), \tag{31}$$

where $x = \frac{\eta(\beta N(\gamma pL - \beta\phi N) - \gamma^2 pL)}{\beta^2 N^2}$, the final eigenvalue is negative once when the term in the exponent (x) is negative:

$$\frac{-\eta(\beta N(\beta\phi N - \gamma pL) + \gamma^2 pL)}{\beta N} < 0 \tag{32}$$

$$(\beta N(\beta\phi N - \gamma pL) + \gamma^2 pL) < 0 \tag{33}$$

$$\frac{\beta N(\beta\phi N - \gamma pL)}{\gamma} < \gamma pL \tag{34}$$

$$\gamma pL\left(\frac{\gamma - \beta N}{\beta^2 N^2}\right) > \phi \tag{35}$$

$$pL\left(\frac{\gamma - \beta N}{\beta N}\right)\left(\frac{\gamma}{\beta N}\right) > \phi. \tag{36}$$

For clarity, this can be re-arranged as follows:

$$\begin{matrix}\text{Cost of} \\ \text{control}\end{matrix} < \begin{matrix}\text{Loss due to} \\ \text{disease}\end{matrix} \times \begin{matrix}\text{Probability of} \\ \text{vertical transmission}\end{matrix} \times \begin{matrix}\text{Proportion of} \\ \text{infected fields}\end{matrix} \times \left(1 - \begin{matrix}\text{Probability of} \\ \text{horizontal transmission}\end{matrix}\right),$$

$$\phi < Lp\left(\frac{\beta N - \gamma}{\beta N}\right)\left(1 - \frac{\beta N - \gamma}{\beta N}\right). \tag{37}$$

That is, for the "all control" equilibrium to be stable, $R_0^H > 1$ and the cost of control (ϕ) must be less than the expected losses of infected non-controllers (L).

1.2.3 Disease-endemic, control-free equilibrium.

The disease-endemic, control-free equilibrium is given by: $(S_C, I_C, I_N) = \left(0, 0, \frac{\beta N - \gamma(1-p)}{\beta}\right)$. Additionally, as there are no controllers at this equilibrium, $P_N = P$ (Equation 23 in the main text). The entries for the Jacobian matrix evaluated at the disease-endemic, no control equilibrium are given as follows:

$$\begin{bmatrix} \frac{\partial \dot{S_C}}{\partial S_C} & \frac{\partial \dot{S_C}}{\partial I_C} & 0 \\ \gamma(R_0 - 1) & -\gamma & 0 \\ \frac{\partial \dot{I_N}}{\partial S_C}^* & \frac{\partial \dot{I_N}}{\partial I_C}^* & \gamma(R_0 - 1) \end{bmatrix} \tag{38}$$

Entries marked with "*" are not written out in full as they are not needed for further analysis. Remaining entries are given as follows:

$$\frac{\partial \dot{S_C}}{\partial S_C} = \gamma \left(\exp\left(-\frac{\eta(\gamma R_0(\beta\phi N - \gamma pL) + \gamma^2 pL)}{\beta\gamma N R_0} \right) - \frac{\gamma\eta(R_0(\beta\phi N - \gamma pL) + \gamma pL)}{\beta N R_0} - R_0 \right) \tag{39}$$

$$\frac{\partial \dot{S_C}}{\partial I_C} = \gamma \left(\exp\left(-\frac{\eta(\gamma R_0(\beta\phi N - \gamma pL) + \gamma^2 pL)}{\beta\gamma N R_0} \right) - \frac{\gamma\eta(R_0(\beta\phi N - \gamma pL) + \gamma pL)}{\beta N R_0} \right) \tag{40}$$

from which we can see that

$$\frac{\partial \dot{S_C}}{\partial S_C} = \frac{\partial \dot{S_C}}{\partial I_C} - \gamma R_0. \tag{41}$$

From this matrix, we can see that the first eigenvalue is given by $\gamma(R_0 - 1)$. This is negative for $R_0 > 1$.

The remaining eigenvalues can be found by solving the equation:

$$\lambda^2 - a_1\lambda + a_2 = 0 \tag{42}$$

where

$$a_1 = \frac{\partial \dot{S_C}}{\partial I_C} - \gamma(R_0 - 1) \tag{43}$$

$$a_2 = -\gamma R_0 \left(\frac{\partial \dot{S_C}}{\partial I_C} - \gamma \right). \tag{44}$$

Solving this, we find the remaining eigenvalues to be: $-\gamma R_0$, and

$$\gamma \left(\exp\left(-\frac{\eta(\gamma R_0(\beta\phi N - \gamma p L) + \gamma^2 p L)}{\beta\gamma N R_0} \right) - 1 + \frac{-\eta(R_0((\beta\phi N - \gamma p L)) - \gamma\eta p L)}{\beta N} \right). \tag{45}$$

Clearly, $-\gamma R_0$ is always negative. The final eigenvalue is negative once

$$1 - \exp\left(-\frac{\eta(\gamma R_0(\beta\phi N - \gamma p L) + \gamma^2 p L)}{\beta\gamma N R_0} \right) < \frac{-\eta(R_0((\beta\phi N - \gamma p L)) - \gamma\eta p L)}{\beta N} \tag{46}$$

That is, the eigenvalue is negative once the term in the exponent is negative. This can be rearranged as follows:

$$Lp \left(\frac{\beta N - \gamma(1-p)}{\beta N} \right) \left(\frac{\gamma}{\beta N + \gamma p} \right) < \phi \tag{47}$$

For clarity, this can be re-written in terms of the probability of infection:

$$\begin{matrix}\text{Loss due to} \\ \text{disease}\end{matrix} \times \begin{matrix}\text{Probability of} \\ \text{vertical transmission}\end{matrix} \times \begin{matrix}\text{Proportion of} \\ \text{infected fields}\end{matrix} \times \left(1 - \begin{matrix}\text{Probability of} \\ \text{horizontal transmission.}\end{matrix} \right) < \begin{matrix}\text{Cost of} \\ \text{control,}\end{matrix} \tag{48}$$

$$Lp \left(\frac{\beta N - \gamma(1-p)}{\beta N} \right) \left(1 - \frac{\beta N - \gamma(1-p)}{\beta N + \gamma p} \right) < \phi. \tag{49}$$

Therefore, the control-free, disease-endemic equilibrium is stable for $R_0 > 1$ and when the expected losses due to disease for an infected non-controller are less than the cost of control.

1.3 Stability of two-strategy equilibrium in the “strategy. vs” models.

Our “strategy vs.” models are discontinuous, as the switching terms take different forms depending on the relative values of the expected profits for each strategy and the population. Due to this discontinuity, the stability of the two-strategy equilibrium for the “strategy vs. population” model cannot be assessed by linearising around the equilibrium. We instead used a numerical approach to assess the stability of this equilibrium. We first generated a parameter set by sampling parameters from a plausible range (given in S3 Table A). We then ran the model for each parameter set and found the equilibrium values for each state variable ($E^* = (S_U^*, I_U^*, S_C^*, I_C^*)$, taken after 500 seasons). Then, for the same parameter set, the model was run with a set of 10,000 different initial conditions again for 500 seasons. The difference between the final equilibrium value for each state variable and E^* was calculated. If the difference was greater than 10^{-8}, it was deemed large enough to conclude that the equilibrium was unstable based on different initial conditions. We repeated this for 5,000 different parameter sets and, in each instance, the equilibrium attained was that expected for the parameter set.

Table A: Range of values used for parameters when evaluating the stability of the “grower vs.” models.

Parameter	Meaning	Range of values
$1/\gamma$	Length of the growing season	$1 - 600$ days
β	Rate of secondary infection	$0 - 2 \times 10^{-5}$ day^{-1} field^{-1}
p	Probability of getting infected cuttings	$0 - 1$
η	Responsiveness of growers	$1 - 200$
L	Loss due to infection	$0 - 1$
ϕ	Cost of control	$0 - 1$
$S_C(0)$	Initial proportion of susceptible controllers	$0 - N$
$I_C(0)$	Initial proportion of infected controllers	$0 - N$
$S_N(0)$	Initial proportion of susceptible non-controllers	$0 - N$
$I_N(0)$	Initial proportion of infected non-controllers	$0 - N$

1.4 Mutual exclusivity of equilibria

For the "no control" equilibrium to be stable, the expected losses due to vertical transmission must be less than the cost of control. That is, from Equation 49, the following must be true:

$$Lp\left(\frac{\beta N-\gamma(1-p)}{\beta N}\right)\left(1-\frac{\beta N-\gamma(1-p)}{\beta N+\gamma p}\right)<\phi \tag{50}$$

Conversely, for the "all control" equilibrium to be stable, the costs must be less than the expected losses due to vertical transmission. From Equation 37, this means that:

$$\phi<Lp\left(\frac{\beta N-\gamma}{\beta N}\right)\left(1-\frac{\beta N-\gamma}{\beta N}\right) \tag{51}$$

Thus, for both equilibria to be stable, the following condition must be met:

$$Lp\left(\frac{\beta N-\gamma(1-p)}{\beta N}\right)\left(1-\frac{\beta N-\gamma(1-p)}{\beta N+\gamma p}\right)<Lp\left(\frac{\beta N-\gamma}{\beta N}\right)\left(1-\frac{\beta N-\gamma}{\beta N}\right) \tag{52}$$

This can be simplified to:

$$\left(\frac{\beta N-\gamma(1-p)}{\beta N+\gamma p}\right)<\left(\frac{\beta N-\gamma}{\beta N}\right), \tag{53}$$

which, in turn, leaves us with the condition that $0<-\gamma^2 p$ must be true. However, as both γ and p are positive parameters, this is not possible and thus both equilibria cannot be simultaneously stable.

1.5 Numerical assessment of equilibria in the "grower vs." models.

The more complex form of these models precludes mathematical analysis, as conditions for model equilibria no longer simply depend on the difference in profits between controllers and non-controllers. For these "grower vs." models to reach a two-strategy equilibrium, the flow between the two strategies

must be equal, i.e.

$$z_{IN}I_N = z_{SC}S_C + z_{IC}I_C \tag{54}$$

Consequently, we evaluated the stability of the possible equilibrium using numerical methods. Using the same method as when assessing the stability of the "two-strategy equilibrium" in the "strategy vs." models, we tested 10,000 sets of initial conditions and ran the model to equilibrium for 5,000 different parameter sets (chosen from a range of the plausible range parameters described in S3 Table A). In no case for either of the "grower vs." models was it found that the difference between equilibrium values of the simulated model was larger than our difference threshold of 10^{-8}. We thus concluded that the final equilibrium attained was not dependent on initial conditions.

The forms of the switching terms will differ between the two "grower vs." models, leading to different equilibria for each model (Fig 3C-D in the main text). There are two important differences from the "strategy vs." models:

- The "all control" equilibrium is impossible because growers who controlled but nevertheless became infected always consider switching strategy, as they are earning the lowest possible payoff (P_{IC}, the "sucker's payoff"). As long as there is a non-zero probability of infection (which is necessary for control to be worthwhile) there will always be non-controlling growers.

- For the "grower vs. population" model, the "no control" equilibrium is only possible if all non-controllers are infected at equilibrium. For a "no control" equilibrium, the expected profit of the population must be less than or equal to P_{IN} to prevent I_N growers switching strategies (i.e. $P \leq P_{IN}$, Equation 34 in the main text). At the "no control" equilibrium $P = P_N$ (Equation 23 in the main text), leading to:

$$P = \frac{P_N(S_N + I_N)}{N} \leq P_{IN} \tag{55}$$

As, in the “no control” equilibrium, $S_N + I_N = N$, this can be simplified:

$$\begin{aligned} P_N &\leq P_{IN} \\ q_N P_{IN} + (1 - q_N) P_{SN} &\leq P_{IN} \\ (1 - q_N) P_{SN} &\leq P_{IN}(1 - q_N) \\ P_{SN} &\leq P_{IN} \end{aligned} \tag{56}$$

The non-zero value of the loss due to disease (L) means that the conclusion presented in Equation 56 is impossible (see Equation 11 & 12 in the main text). Therefore, for a “no-control” equilibrium to be possible, all fields must be infected (i.e. $N = I_N$ and $q_N = 1$). In Fig 2B (main text), a “no control” equilibrium would require higher β and p (Fig A).

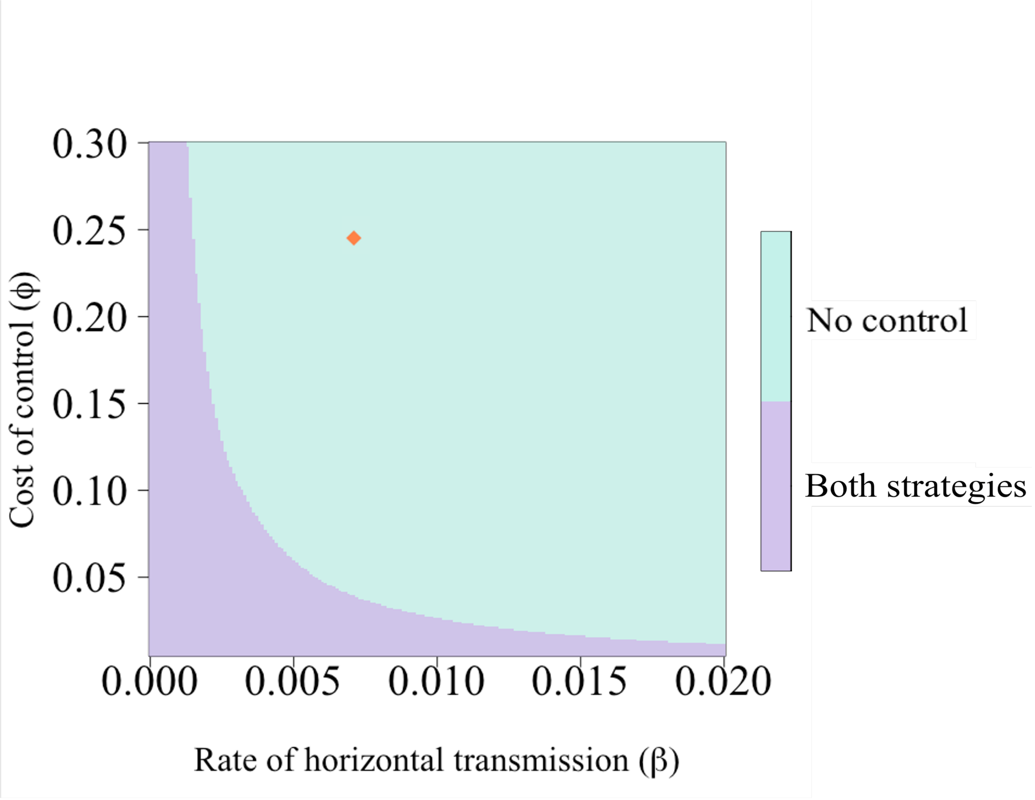

Fig A: Possible equilibria for the "grower vs. population" model when $p = 1$. With the higher probability of vertical transmission, the "no control" equilibrium is possible for the "grower vs. population" model as there will be no non-infected, non-controlling (S_N) growers at equilibrium (Equation 56). However, now that $p = 1$, there can never be a disease-free equilibrium for this parameter set (as $R_0 > 1$).

Additionally, unlike in the "strategy vs." models, where the equilibrium is not dependent on the value of responsiveness of growers (η), for the "growers vs." models η does affect the final equilibrium values (see also Fig 1 in S4 Text).

References

[1] Diekmann O, Heesterbeek JAP, Roberts MG. The construction of next-generation matrices for compartmental epidemic models. Journal of The Royal Society Interface. 2010;7(47):873–885. doi:10.1098/rsif.2009.0386.

[2] van den Driessche P. Reproduction numbers of infectious disease models. Infectious Disease Modelling. 2017;2(3):288–303. doi:https://doi.org/10.1016/j.idm.2017.06.002.

[3] Hamelin FM, Bowen B, Bernhard P, Bokil VA. Optimal Control of Plant Disease Epidemics with Clean Seed Usage. Bulletin of mathematical biology. 2021;83 5:46.
