# Supplementary material for: How growers make decisions impacts plant disease control
Source: PLoS Comput Biol. 2022 Aug 22;18(8):e1010309. doi: 10.1371/journal.pcbi.1010309 (PMC9394827; doi:10.1371/journal.pcbi.1010309)
Supplement: S4 Text — Fig A: Effect of changes in responsiveness and horizontal transmission on the proportion of controllers at equilibrium. Below a certain threshold, there is a decrease in the proportion of controllers with an increase in η, as the higher responsiveness causes more SC growers to switch strategy. Above this threshold, an increase in η causes an increase in controllers. The parameter values mean that SC growers no longer change strategy, so they cannot leave the CSS. However, an increase in η means that IN growers have a higher probability of switching into the CSS. The solid vertical line denotes where this threshold is crossed (β = 0.03301 day −1). Fig B: Effect of responsiveness (η) on the “grower vs. alternative” model. A The flow of growers between the non-infected controllers (SC), infected controllers (IC) and infected non-controllers (IN) based on their probability of switching strategy (zSC, zIC and zIN respectively) with η = 1. B Full model dynamics for η = 1. C The flow of growers between SC, IC and IN based on their probability of switching with η = 10. Note that will lower values of η, there are fewer growers moving between strategies. D Full model dynamics for η = 10. Parameters are as in Table 1, except for β = 0.004 day −1, which was used to allow for an disease- and control-endemic equilibrium (Fig 2). Fig C: Effect of parameters on participation in the CSS. A—C shows results for the “strategy vs. population” and “strategy vs. alternative” model formulations; D—F are for the “grower vs. population” model. Parameters are as in Table 1 in the main text; the orange diamonds mark the default values. A and D examine the impact of changing the cost of control (ϕ) and the rate of horizontal transmission (β). At low values of β, no-one should control as the probability of infection is sufficiently low that it is not necessary. As β increases, controlling is more beneficial and the cost of control is perceived to be worthwhile. However, as infection becomes more [file pcbi.1010309.s004.pdf]

1 S4: Supplementary results

1.1 Effect of responsiveness (η) on the "grower vs." models.

Unlike in the "strategy vs." models, where the equilibrium values are not dependent on the value of responsiveness of growers, for the "growers vs." models η does affect the final equilibrium values. Fig A shows how the proportion of controllers changes with varying values of horizontal transmission (β) and η.

Interestingly, there is a directionality to the response. Below a certain threshold (which here is $\beta = 0.003301$ day $^{-1}$), an increase in η decreases the proportion controlling. The increase in η means that all growers who have the potential to switch strategy have a higher probability of doing so, and if η is sufficiently high then each switching term approaches 1. This means that there is a decrease in controllers, as all S_C and I_C growers will switch strategy, though only I_N growers will start controlling.

At higher values of β, the increase in η causes an increase in controllers. For these values, z_{sc} eventually falls to zero, and having higher values of η increases the rate at which this happens. As the parameter values permit an two-strategy equilibrium, but the S_C growers are no longer switching strategy, there is an increase in the number of controllers with an increase in η.

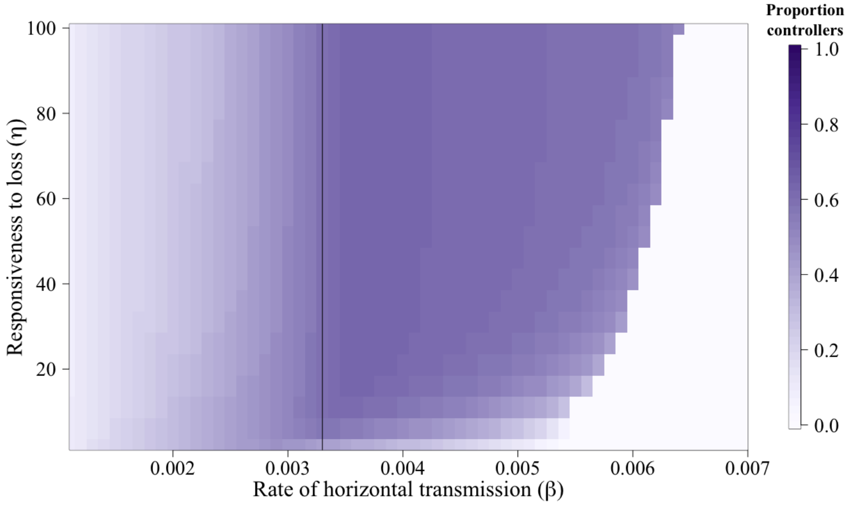

Fig A: Effect of changes in responsiveness and horizontal transmission on the proportion of controllers at equilibrium. Below a certain threshold, there is a decrease in the proportion of controllers with an increase in η, as the higher responsiveness causes more S_C growers to switch strategy. Above this threshold, an increase in η causes an increase in controllers. The parameter values mean that S_C growers no longer change strategy, so they cannot leave the CSS. However, an increase in η means that I_N growers have a higher probability of switching into the CSS. The solid vertical line denotes where this threshold is crossed ($\beta = 0.03301$ day -1).

In the "grower vs." models, equilibrium is reached when $z_{SC}S_C + z_{IC}I_C = z_{IN}I_N$ (i.e. the flow of growers out of one strategy matches the flow of growers out of the other) (Fig B). As the switching terms are in part set by the value of η, for changes in responsiveness then the values of S_C, I_C and I_N at equilibrium must change to ensure the equilibrium condition is still met.

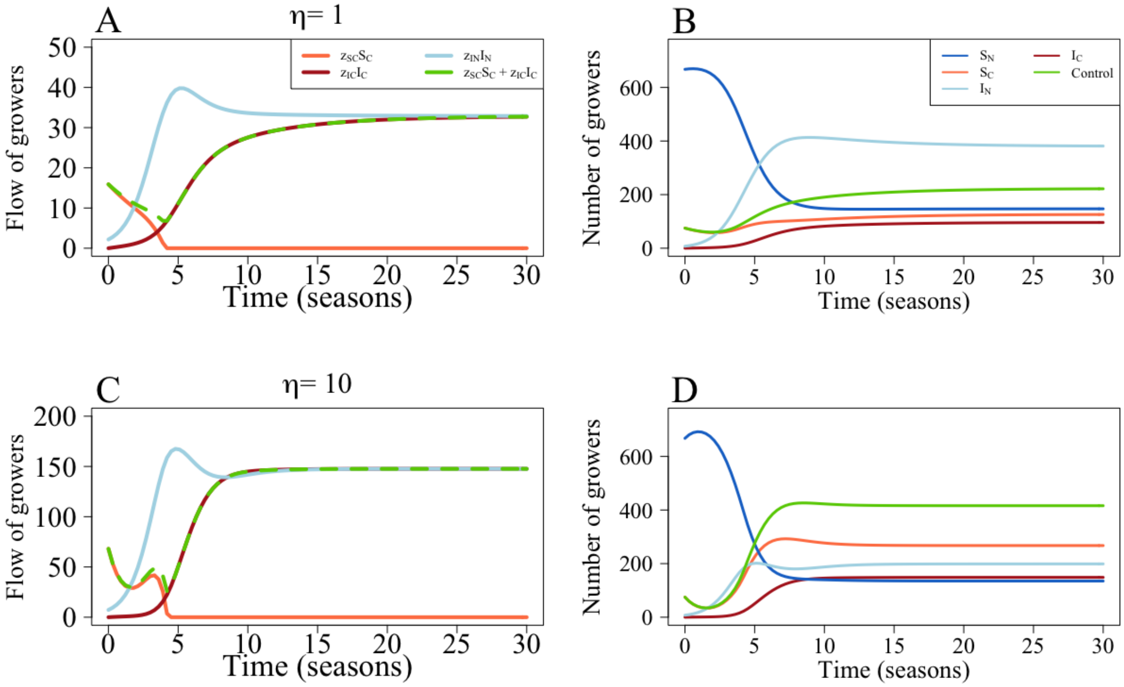

Fig B: Effect of responsiveness (η) on the "grower vs. alternative" model. A The flow of growers between the non-infected controllers (S_C), infected controllers (I_C) and infected non-controllers (I_N) based on their probability of switching strategy (z_{SC}, z_{IC} and z_{IN} respectively) with $\eta = 1$. B Full model dynamics for $\eta = 1$. C The flow of growers between S_C, I_C and I_N based on their probability of switching with $\eta = 10$. Note that will lower values of η, there are fewer growers moving between strategies. D Full model dynamics for $\eta = 10$. Parameters are as in Table 1 in the main text, except for $\beta = 0.004$ day -1, which was used to allow for an disease- and control-endemic equilibrium (Fig 2 in the main text).

1.2 Effect of parameters on the "strategy vs." and "grower vs. population" models.

We will now consider the three other behavioural models (the "strategy vs. population", "strategy vs. alternative" and "grower vs. population") that were not a focus of the main text. Each model formulation responded very differently to changes in parameter values. In all cases, when the rate of horizontal transmission (β) is sufficiently low, no grower should use the CSS (Fig C). At medium-to-low values of β, more growers use the CSS, though as β increased, the higher probability of infection narrows the range of costs for which a controller will consider participation in the CSS. The two "strategy vs." models - which had the same equilibria" - allowed an "all control" equilibrium at low costs of control (ϕ). This was never possible for the "grower vs. population model, as controllers managing infected fields should always consider switching strategy as they have received the "sucker's payoff".

Overall, the "strategy vs." models saw lower participation in the CSS than the "grower vs." models. The high default value of the cost of control ($\phi = 0.25$) means that growers will participate in the CSS only at very high probabilities of vertical transmission (p) and loss due to disease (L). In the "grower vs." models, however, the non-controllers with infected fields will likely have a lower payoff than the population average, and thus should have a higher probability of switching strategy for a wider range of these parameter values.

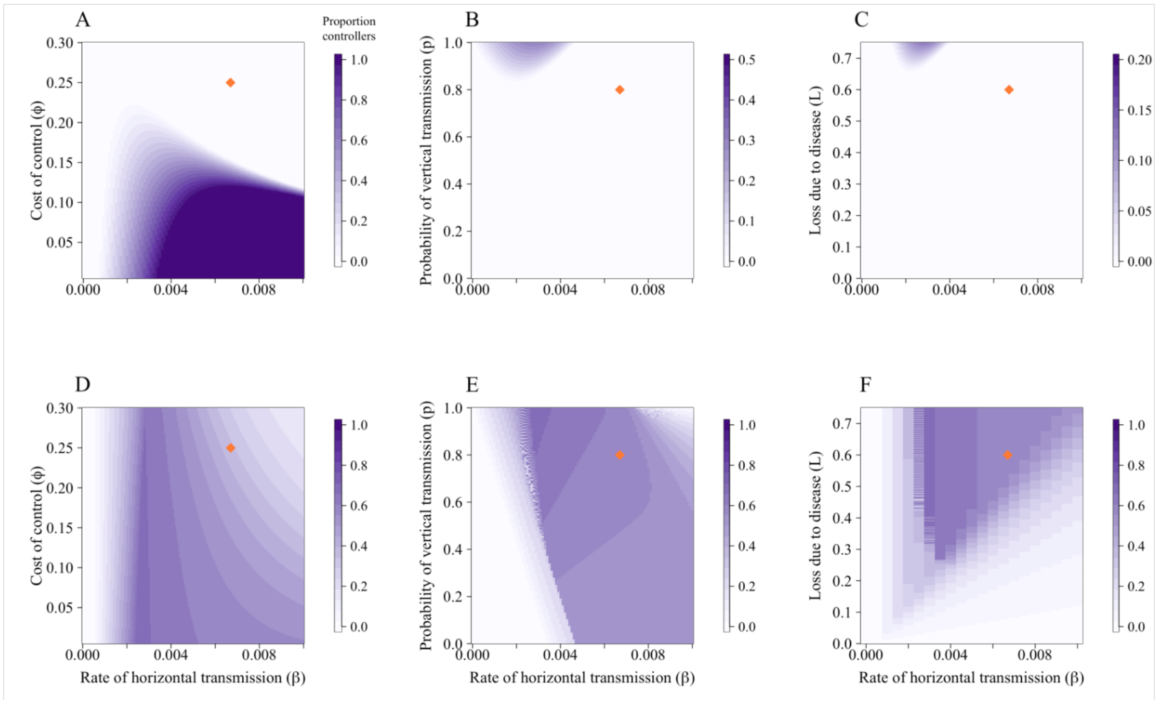

Fig C: Effect of parameters on participation in the CSS. A – C shows results for the "strategy vs. population" and "strategy vs. alternative" model formulations; D – F are for the "grower vs. population" model. Parameters are as in Table 1 in the main text; the orange diamonds mark the default values. A and D examine the impact of changing the cost of control (ϕ) and the rate of horizontal transmission (β). At low values of β, no-one should control as the probability of infection is sufficiently low that it is not necessary. As β increases, controlling is more beneficial and the cost of control is perceived to be worthwhile. However, as infection becomes more likely, the value of control diminishes so it is not worthwhile to invest in control. In the "strategy vs." comparisons, it is possible to reach an "all control" equilibrium, though this cannot happen for the "grower vs. population" model. B and C At very high probabilities of vertical transmission (p) and loss due to disease (L), growers will participate in the CSS, though only for a narrow range of β. However, for the "grower vs. population" models, a much wider range of all parameters allowed for participation. The irregular contours in E and F are due to oscillations around the equilibrium.

1.3 Effect of subsidy on parameter scans.

Using the default parameters, there is a narrow range of parameter values for which growers should consider control at equilibrium in the "strategy vs." models (Fig DD). In the "grower vs." models, though there is a wider range of parameters for which growers control at equilibrium, there are still low levels of participation in the CSS (Fig DE-F). Even when the probability of vertical transmission is high, there are few growers using the CSS due to the high cost of participation. However, providing

a 50% subsidy such that $\phi = 0.125$ increases both the level of participation and the range of parameter values for which growers control (Fig D).

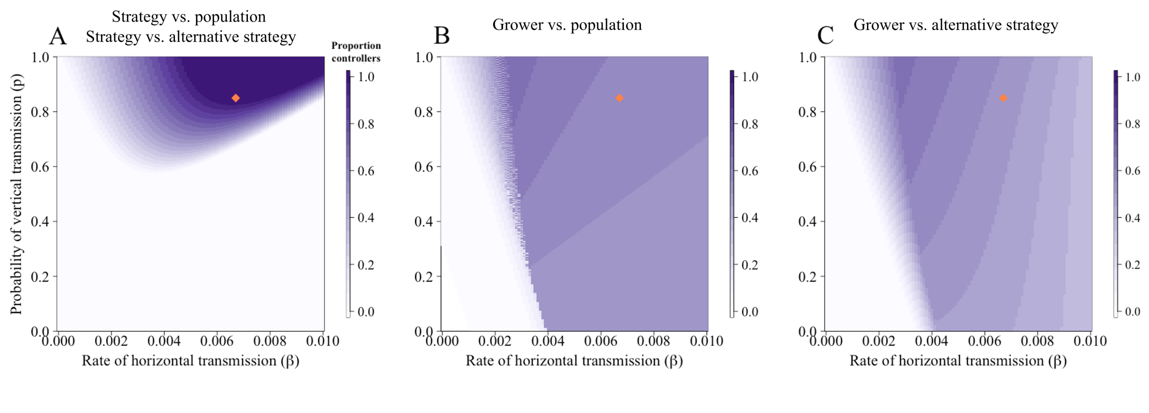

Fig D: Effect of changes in the rate of secondary transmission (β) and probability of vertical transmission (p) on the proportion of controllers when $\phi = 0.125$. Compared to the default value of $\phi = 0.25$, a higher proportion of growers control for a broader range of parameter values.
