# Supplementary material for: How growers make decisions impacts plant disease control
Source: PLoS Comput Biol. 2022 Aug 22;18(8):e1010309. doi: 10.1371/journal.pcbi.1010309 (PMC9394827; doi:10.1371/journal.pcbi.1010309)
Supplement: Attachment — Submitted filename: PCOMPBIOL-D-21-02295_review.pdf [file pcbi.1010309.s006.pdf]

PLOS Computational Biology

It ain't what you do, it's what drives why you do it: how the basis of growers' decision making affects the effectiveness of disease control

--Manuscript Draft--

**Manuscript Number:**	PCOMPBIOL-D-21-02295
**Full Title:**	It ain't what you do, it's what drives why you do it: how the basis of growers' decision making affects the effectiveness of disease control
**Short Title:**	Human behaviour and plant disease control management
**Article Type:**	Research Article
**Keywords:**	epidemiological model, behavioural model, game theory, clean seed systems, cassava, cassava brown streak disease
**Abstract:**	While the spread of plant disease depends strongly on biological factors controlling transmission, ~~epidemics clearly~~ also have a human dimension. Disease control depends on decisions made by individual growers, who are in turn influenced by a broad range of factors. Despite this, human behaviour has rarely been included in plant epidemic models. Considering Cassava Brown Streak Disease, we model how the perceived benefit of disease control influences participation in clean seed systems (CSS). Our models are rooted in game theory, with growers making strategic decisions based on the expected profitability of different control strategies. We find that both the information used by growers to assess profitability and the perception of economic and epidemiological parameters influence long-term participation in the CSS. Over-estimation of infection risk leads to lower participation in the CSS, as growers perceive that paying for the CSS will be futile. Additionally, though the CSS can reduce the burden of disease, and allow a scenario in which all growers control, disease is not eliminated from the system. These results are robust to stochastic and spatial effects. Our work highlights the importance of including human behaviour in plant disease models, but also the significance of how that behaviour is included.
**Additional Information:**	
**Question**	**Response**
**Financial Disclosure**<br><br>Enter a financial disclosure statement that describes the sources of funding for the work included in this submission. Review the submission guidelines for detailed requirements. View published research articles from *PLOS Computational Biology* for specific examples.<br><br>This statement is required for submission and **will appear in the published article** if the submission is accepted. Please make sure it is accurate.	REMW acknowledges the Biotechnology and Biological Sciences Research Council of the United Kingdom (BBSRC; \texttt{https://bbsrc.ukri.org/}) for support via a University of Cambridge DTP PhD studentship (Project Reference 2119272). The funders had no role in study design, data collection and analysis, decision to publish, or preparation of the manuscript.

**Unfunded studies**<br>Enter: *The author(s) received no specific funding for this work.*<br><br>**Funded studies**<br>Enter a statement with the following details:<br>• Initials of the authors who received each award<br>• Grant numbers awarded to each author<br>• The full name of each funder<br>• URL of each funder website<br>• Did the sponsors or funders play any role in the study design, data collection and analysis, decision to publish, or preparation of the manuscript?<br>• **NO** - Include this sentence at the end of your statement: *The funders had no role in study design, data collection and analysis, decision to publish, or preparation of the manuscript.*<br>• **YES** - Specify the role(s) played.<br><br>* typeset	
**Competing Interests**<br><br>Use the instructions below to enter a competing interest statement for this submission. On behalf of all authors, disclose any competing interests that could be perceived to bias this work—acknowledging all financial support and any other relevant financial or non-financial competing interests.<br><br>This statement **will appear in the published article** if the submission is accepted. Please make sure it is accurate. View published research articles from *PLOS Computational Biology* for specific examples.	The authors have declared that no competing interests exist.

Powered by Editorial Manager® and ProduXion Manager® from Aries Systems Corporation

**NO authors have competing interests**<br><br>Enter: *The authors have declared that no competing interests exist.*<br><br>**Authors with competing interests**<br><br>Enter competing interest details beginning with this statement:<br><br>*I have read the journal's policy and the authors of this manuscript have the following competing interests: [insert competing interests here]*<br><br>* typeset<br><br>This statement is required for submission and **will appear in the published article** if the submission is accepted. Please make sure it is accurate and that any funding sources listed in your Funding Information later in the submission form are also declared in your Financial Disclosure statement.	
**Data and Code Availability**<br><br>From the time of publication, Authors are required to make fully available and without restriction all data and computational code underlying their findings. Please see our PLOS Data Policy page for detailed policy information, and our Code Sharing page for specific information on code sharing.<br><br>A **Data Availability Statement**, detailing where the data (and code, if applicable) can be accessed, is required at first submission. Insert your Data Availability Statement in the box below. The statement you provide **will be published in the article**, if accepted.<br><br>Please see the Data Reporting section of our submission guidelines for instructions on what you need to include in your Data Availability Statement.	The code used in the models will be made available upon publication.

Powered by Editorial Manager® and ProduXion Manager® from Aries Systems Corporation

If the data and code are all contained in your submission files, please state: *All relevant data are within the manuscript and its Supporting Information files.*<br><br>**PLOS allows rare exemptions to address legal and ethical concerns.** If you have legal or ethical restrictions, please detail these in your Data Availability Statement below for the Journal team to consider.	

Manuscript
Click here to access/download;Manuscript;Grower_behaviour_paper-1-49.pdf

It ain't what you do, it's what drives why you do it: how the basis of growers' decision making affects the effectiveness of disease control

Rachel E. Murray-Watson *[1], Frédéric M. Hamelin[2], and Nik J. Cunniffe[1]

[1]Department of Plant Sciences, University of Cambridge, Cambridge, United Kingdom

[2]IGEPP, INRAE, Institut Agro, Univ Rennes, Rennes, France

Short title: Game theory and plant epidemic management.

*For correspondence: rm844@cam.ac.uk

Abstract

While the spread of plant disease depends strongly on biological factors controlling transmission, epidemics clearly also have a human dimension. Disease control depends on decisions made by individual growers, who are in turn influenced by a broad range of factors. Despite this, human behaviour has rarely been included in plant epidemic models. Considering Cassava Brown Streak Disease, we model how the perceived benefit of disease control influences participation in clean seed systems (CSS). Our models are rooted in game theory, with growers making strategic decisions based on the expected profitability of different control strategies. We find that both the information used by growers to assess profitability and the perception of economic and epidemiological parameters influence long-term participation in the CSS. Over-estimation of infection risk leads to lower participation in the CSS, as growers perceive that paying for the CSS will be futile. Additionally, though the CSS can reduce the burden of disease, and allow a scenario in which all growers control, disease is not eliminated from the system. These results are robust to stochastic and spatial effects. Our work highlights the importance of including human behaviour in plant disease models, but also the significance of how that behaviour is included.

1 Introduction

Human behaviour is intuitively important in the control of infectious disease, as individuals often face choices when adopting infection-limiting behaviours. This has been widely considered in the context of human (e.g. [1]; reviewed in [2]) and animal (e.g. [3], [4]) diseases, though there have been fewer studies for plant diseases ([5], [6], [7], [8], [9]). Where studies do consider individual growers' decisions there is little justification of how decision-making is modelled, although it clearly will affect the outcomes of their decisions.

Decisions regarding disease control are intrinsically linked to disease prevalence. Clearly, they must also depend upon considerations such as the cost of control, the consequences of infection

and the perceived risk of becoming infected. Additionally, and crucially, decisions made by one grower will influence the decisions of another. Voluntary disease control programmes can be viewed as a collective action problem (also termed social dilemmas; [10]) as they generate externalities that affect the outcomes of those not involved in the programme. By reducing or eliminating their own chance of infection, growers also lower the probability that their neighbours will be infected, thereby generating a positive externality that disincentives their neighbours' engagement in disease prevention [11].

Such "free-riding" behaviour is indirectly encouraged when there is higher overall participation in control schemes, as growers are less likely to become infected whenever the proportion controlling is high. Yet if too many growers engage in free-riding behaviour, there will be a resurgence of infections, making disease control more likely. These feedback loops are believed to be a major reason why some public health schemes for human disease, such as subsidised vaccination campaigns, have failed to lead to disease eradication [11]. Consequently, the success of voluntary control policies may be self-limiting.

The strategic nature of these behaviours lends itself to game-theoretic analysis. ~~Game theory is used to study decision making in settings where an individual (or "player") can choose from a variety of strategies, with the aim of choosing one that will maximise their desired outcome ("payoff") [12]. No individual makes a decision in isolation, and the best strategy to play will depend on the strategy choices of others.~~

Game theoretic approaches have been used in human and veterinary disease modelling, often based on evolutionary game theory (~~for example, [13] and [3] both use replicator-dynamic inspired approaches to model vaccination uptake~~). In this formalism, in a two-strategy model, the performance of the player's own strategy is compared against that of the average performance of the population. If the payoff is less than that of the population, the focal player changes strategy; if it is higher, they remain with their current strategy. For plant disease, this would involve growers comparing the average profit of all of those using their own control strategy with the average

profit growers in the population (we call this the "strategy vs. population" model). Of course this assumes, perhaps unrealistically, that growers will consider the profit of all those using the same control strategy.

Alternatively, in both [5] and [6], growers compare their own profit with the average profit of those playing the alternative strategy (henceforth the "grower vs. alternative" model). Ostensibly this seems a more likely comparison a grower will make, as it requires less information. Other models combine elements of both approaches, since growers compare their strategy with the expected profit of the alternative strategy ("strategy vs. alternative" model) or compare their own profit with the average of the population ("grower vs. population" model). To our knowledge, the "grower vs." models have never been compared ~~to date in publication~~.

To examine the effects of these different models of growers' decision making, we use a simple model describing the spread of Cassava Brown Streak Disease (CBSD) based on the model presented in [6]. Cassava (*Manihot esculenta*) is a staple food in Sub-Saharan Africa, where it is predominantly grown as a subsistence crop. Cassava production is threatened by CBSD, a viral disease caused by either cassava brown streak virus (CBSV, [14]) or Ugandan CBSV (UCBSV; [15]). Infection results in necrosis of the stems and tubers, leading to yield losses of up to 70% [16]. Coupled with its increasing spread from east to west Africa, this positions CBSD as a major challenge to cassava cultivation across the region [17].

Both viruses are transmitted horizontally by a whitefly vector (*Bemisia tabaci*, [18]) and vertically through the replanting of infected stem cuttings. Informal trade of cassava cuttings is widespread amongst growers, though the subtlety of symptoms often means that the practice often contributes to vegetative propagation of infected material [19] and long-range spread of CBSD between growers [20].

These transmission pathways mean that clean seed systems (CSSs) are a plausible CBSD management strategy [21]. CSSs disseminate virus-free planting material, thereby avoiding the vegetative propagation of infected material. The success of CSSs will depend on grower behaviour, as participa-

tion will be governed by the balance of costs and benefits of the scheme. This, and the combination of trade- and whitefly-mediated pathogen transmission, means that a grower's profit will depend on the action of others, making the system amenable to game theoretic analysis.

The overall objective of this work is to investigate how including grower behaviour in plant epidemic models affects the outcome of control schemes, and how the factors influencing behaviour can best be manipulated to encourage control uptake. We use our behavioural model alongside the CBSD case study to address the following: (1) When a control strategy provides incomplete protection from disease, can a scenario where all growers adopt control be attained and maintained in a population? (2) How does grower participation in the CSS depend on epidemiologically and economically important parameters? (3) How do these results differ when the quantities of comparisons driving growers' decision-making change? (4) How does systemic uncertainty in epidemiological parameters affect participation in the CSS? (5) Are the results affected by adding stochasticity and space to the model?

2 Materials and Methods

2.1 Non-spatial epidemiological model

Our underlying non-spatial model (Figure 1(A)) is a simplified representation of a CSS. Fields are classified by infection status (susceptible, S, or infected, I), as well as whether their growers currently control, i.e. used certified clean seed the last time the field was planted (subscripts: controller, C, or non-controller, N). We assume each grower cultivates only a single field, and so use the terms "grower" and "field" synonymously. Infection status and control status are binary classifications; we do not model within-field spread of disease, and assume controllers plant only certified virus-free material.

Susceptible fields are vulnerable to horizontal infection (at rate β) due to viruliferous vectors spreading from infected fields (fields of class I_C and I_N) (Table 1). Vertical infection also occurs via

infected propagation material. We assume the clean seed program is effective, and so the propagation material used by controllers is never infected. The probability of a non-controller becoming vertically infected ($p_N(\text{Vert})$, Equation 19) depends jointly on the probability of acquiring infected planting material from an individual infected field (p) and the prevalence of disease at the time of planting $\left(\frac{I_C+I_N}{N}\right)$. The season length ($1/\gamma$) is independent of the control strategy adopted in, and infection status of, any given field. Since we use a continuous time model, fields are asynchronously harvested and replanted [22], a plausible assumption for cassava cultivation in much of Sub-Saharan Africa [23].

To integrate behavioural dynamics, we represent the decisions made by growers at the time of planting by switching terms. These reflect the probability of growers switching strategy for their following crop, based on their current strategy and the current state of the system, as well as, potentially, whether their previous crop was infected. In general, we distinguish four switching terms (Figure 1(A)).

$$z_{SC} = \mathbb{P}\left(\text{switch from } C \text{ to } N|\text{previous crop remained uninfected } (S)\right), \quad (1)$$

$$z_{IC} = \mathbb{P}\left(\text{switch from } C \text{ to } N|\text{previous crop was infected } (I)\right), \quad (2)$$

$$z_{SN} = \mathbb{P}\left(\text{switch from } N \text{ to } C|\text{previous crop remained uninfected } (S)\right), \quad (3)$$

$$z_{IN} = \mathbb{P}\left(\text{switch from } N \text{ to } C|\text{previous crop was infected } (I)\right). \quad (4)$$

As described below, the probabilities encoded in the switching terms depend on growers' assessments of likely profits, and thus depend on the comparisons we assume are made by growers.

Table 1: Summary of parameter values. Parameters below the horizontal line are only required in the spatially-explicit version of the model.

Parameter	Meaning	Value	Reference
$1/\gamma$	Length of the growing season	300 days	[6]; [24]
β	Rate of secondary infection	8.93×10^{-6} day^{-1} field^{-1}	Calibrated to [6]
p	Probability of planting infected cuttings	0.8	Assumed (see main text)
η	Responsiveness of growers	10	Assumed (see main text)
Y	Maximum yield	1	All values scaled relative to yield
L	Loss due to infection	0.6	[16]; [25]
ϕ	Cost of control	0.25	[6]
N	Total number of fields/growers	750	Illustrative
$S_C(0)$	Initial proportion of susceptible controllers	$0.1N$	Illustrative
$I_C(0)$	Initial proportion of infected controllers	0	Illustrative
$S_N(0)$	Initial proportion of susceptible non-controllers	$0.89N$	Illustrative
$I_N(0)$	Initial proportion of infected non-controllers	$0.01N$	Illustrative
α	Dispersal scale parameter for whitefly	150 m	[6]; [26]
β_S	Effective rate of secondary infection (spatial)	110 day^{-1} field^{-1}	Calibrated to [6]

Our general non-spatial model is

$$\frac{dS_C}{dt} = \gamma\left(S_C(1 - z_{SC}) + I_C(1 - z_{IC}) + S_N z_{SN} + I_N z_{IN}\right) - \beta S_C\left(I_C + I_N\right) - \gamma S_C, \tag{5}$$

$$\frac{dI_C}{dt} = \beta S_C\left(I_C + I_N\right) - \gamma I_C, \tag{6}$$

$$\frac{dS_N}{dt} = \gamma\left(S_C z_{SC} + I_C z_{IC} + S_N(1 - z_{SN}) + I_N(1 - z_{IN})\right)\left(1 - \frac{p(I_C + I_N)}{N}\right) - \beta S_N\left(I_C + I_N\right) - \gamma S_N, \tag{7}$$

$$\frac{dI_N}{dt} = \gamma\left(S_C z_{SC} + I_C z_{IC} + S_N(1 - z_{SN}) + I_N(1 - z_{IN})\right)\left(\frac{p(I_C + I_N)}{N}\right) + \beta S_N\left(I_C + I_N\right) - \gamma I_N. \tag{8}$$

2.2 Strategies, outcomes and profits

For simplicity, we assume only a single control strategy is available, the CSS. Thus, there are only two pure strategies available to growers at the beginning of each season: to control for disease, or to not control (Figure 1(B)). Within each strategy, there are two outcomes realised at the time of harvesting: the grower's field may remain uninfected or have become infected.

Each strategy × outcome combination results in a different payoff (Figure 1(B)). An uninfected field generates yield Y, with loss of income, L, in infected fields. Control incurs a cost ϕ. The profit for each outcome is (Table 3):

$$P_{SN} = \text{Profit for non-controller who remains susceptible} = Y, \tag{9}$$

$$P_{IN} = \text{Profit for non-controller who becomes infected} = Y - L, \tag{10}$$

$$P_{SC} = \text{Profit for controller who remains susceptible} = Y - \phi, \tag{11}$$

$$P_{IC} = \text{Profit for controller who becomes infected} = Y - \phi - L, \tag{12}$$

Rational growers will never control if $L < \phi$, since then the cost of clean seed exceeds the loss due to infection. We therefore only consider $L > \phi$, from which it follows that:

$$P_{SN} > P_{SC} > P_{IN} > P_{IC}. \tag{13}$$

2.3 Risks of infection

We initially focus on the case in which growers can estimate risks of infection relatively precisely. In particular, we assume that growers use their current instantaneous probability of infection as an

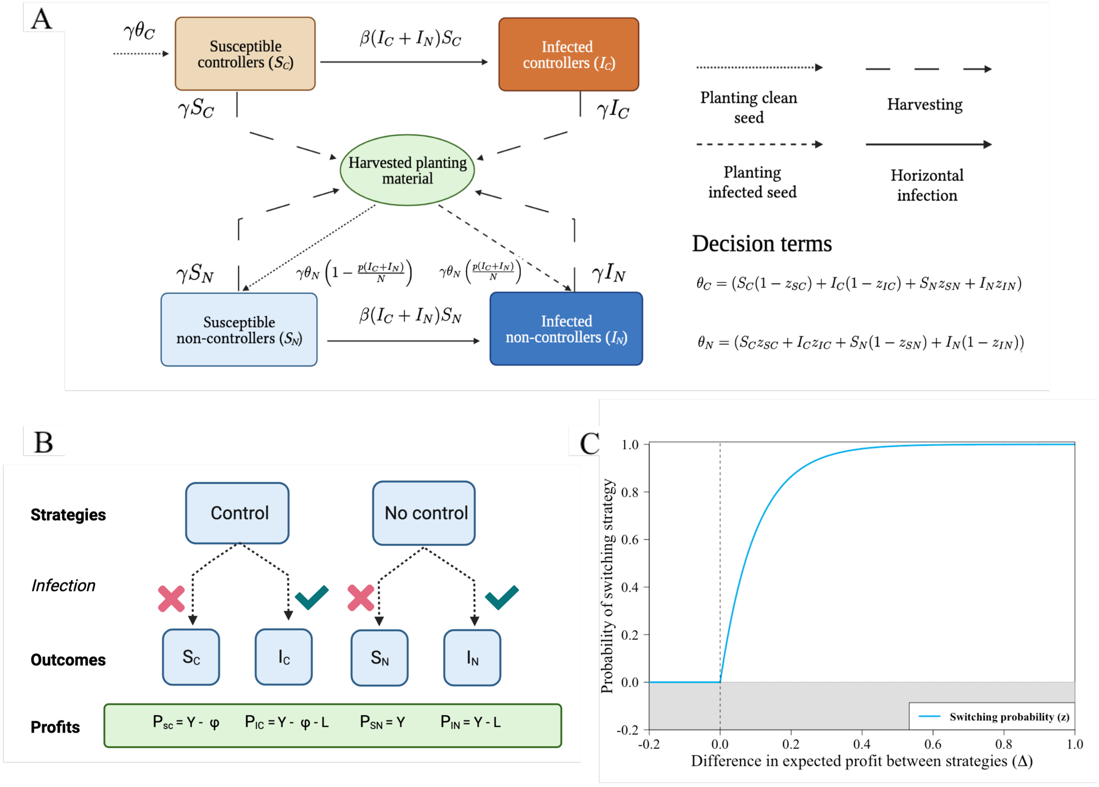

Figure 1: Epidemiological and behavioural models. (A) The epidemiological model distinguishes growers who control (i.e. plant clean seed) from those who do not. Only non-controllers can become infected via vertical transmission, although all growers are subject to horizontal transmission due to movement of vectors from infected fields (Equations 5-8). When replanting growers potentially switch strategies. (B) Strategies, outcomes and profits; the latter account for the loss of yield if infected and the cost of participating in the control scheme. (C) Decisions to switch are based on a switching function, parameterising the probability of switching strategy z as a function of Δ as difference in predicted profits. Figures (A) and (B) were created with BioRender.com.

estimate of their instantaneous probability of horizontal infection over the next season:

$$\begin{aligned} \begin{array}{c}\text{Instantaneous probability}\\ \text{of horizontal infection}\end{array} &= \frac{\text{Instantaneous infection rate}}{\text{Instantaneous infection rate + Harvesting rate}}, && (14)\\ &= \frac{\beta(I_C + I_N)}{\beta(I_C + I_N) + \gamma}. && (15)\end{aligned}$$

For controllers, clean seed rules out vertical transmission. Equation 15 therefore sets a grower's estimate of the probability of infection next season if they were to choose to control.

$$\begin{aligned} q_C &= \text{Grower's estimate of the probability of infection next season if control is adopted}, && (16)\\ &= \frac{\beta(I_C + I_N)}{\beta(I_C + I_N) + \gamma}. && (17)\end{aligned}$$

For non-controllers, vertical infection must also be considered. We again assume growers can estimate the relevant instantaneous probability of vertical transmission (p_N(Vert)) with perfect accuracy:

$$\begin{aligned} p_N(\text{Vert}) &= \frac{\text{Probability of vertical transmission}}{\text{Total number of fields}}, && (18)\\ &= \frac{p(I_C + I_N)}{N}. && (19)\end{aligned}$$

A non-controller's instantaneous probability of horizontal infection (p(Horiz)) must account for the fact that they may first be infected via vertical transmission, leading to:

$$\begin{aligned} p_N(\text{Horiz}) &= \begin{array}{c}\text{Instantaneous probability}\\ \text{of horizontal transmission}\end{array} \times \left(1 - \begin{array}{c}\text{Instantaneous probability}\\ \text{of vertical transmission}\end{array}\right), && (20)\\ &= \left(\frac{\beta(I_C + I_N)}{\beta(I_C + I_N) + \gamma}\right)\left(1 - \frac{p(I_C + I_N)}{N}\right). && (21)\end{aligned}$$

Combining these gives us the instantaneous probability of infection for a non-controller:

$$
\begin{aligned}
q_N &= \text{Grower's estimate of the probability of infection next season if control is not adopted,} && (22)\\
&= p_N(Vert) + p_N(Horiz), && (23)\\
&= \frac{p(I_C + I_N)}{N} + \left(\frac{\beta(I_C + I_N)}{\beta(I_C + I_N) + \gamma}\right)\left(1 - \frac{p(I_C + I_N)}{N}\right). && (24)
\end{aligned}
$$

2.4 Expected profits

Our behavioural models assume that – at the time of planting – individual growers estimate the expected profit for the next season of adopting each strategy. These depend on the payoffs for each strategy × outcome combination (Equations 9-12) and the estimated probabilities of infection under each strategy (Equations 17 and 24). In particular, the estimated expected profit for a grower who controls would be

$$
\begin{aligned}
P_C &= \text{Grower's estimate of the expected profit next season if control is adopted,} && (25)\\
&= q_c P_{IC} + (1 - q_c) P_{SC}, && (26)\\
&= Y - \phi - L\left(\frac{\beta(I_C + I_N)}{\beta(I_C + I_N) + \gamma}\right). && (27)
\end{aligned}
$$

The corresponding estimate without control is

$$
\begin{aligned}
P_N &= \text{Grower's estimate of the expected profit next season if control is not adopted,} && (28)\\
&= q_N P_{IN} + (1 - q_N) P_{SN} && (29)\\
&= Y - L\left(\frac{p(I_C + I_N)}{N} + \left(\frac{\beta(I_C + I_N)}{\beta(I_C + I_N) + \gamma}\right)\left(1 - \frac{p(I_C + I_N)}{N}\right)\right). && (30)
\end{aligned}
$$

The expected profit averaged over all growers is

$$\begin{aligned} P &= \text{Grower's estimate of the expected profit next season averaged over the population}, \qquad (31) \\ &= P_C \left(\frac{S_C + I_C}{N} \right) + P_N \left(\frac{S_N + I_N}{N} \right). \qquad (32) \end{aligned}$$

Estimating P therefore requires growers to know the proportion of the population controlling, and the estimate is instantaneous, i.e. based on the state of the system at the time of planting.

2.5 Behavioural models

We systematically examine different ways to construct the switching terms in Equations 5 - 8. All set z, the probability of an individual grower switching strategy, as a function of Δ, an expected difference in profit, via

$$z = \max\left(0, 1 - e^{-\eta\Delta}\right) = \begin{cases} 1 - e^{-\eta\Delta} & \text{if } \Delta \geq 0 \\ 0 & \text{otherwise.} \end{cases} \qquad (33)$$

Since growers aim to maximise their profit, they only consider switching strategy when the comparison appears to offer a higher payoff (i.e. when $\Delta > 0$; Figure 1(C)). The parameter η sets the responsiveness of growers to a unit difference in profit.

[illegible]erences between behavioural models turn on how Δ is estimated. We consider four possible comparisons.

- Strategy vs. population. Growers compare the expected profit for their current strategy with the expected profit across the entire population of growers.
- Strategy vs. alternative strategy. Growers compare the expected profit for their current strategy with the expected profit of the alternative strategy.
- Grower vs. population. Growers compare their own outcome during the previous season with

Table 2: Summary of the information required by growers to carry out each means of comparison. "Controller prevalence" refers to the proportion of growers that adopted control the last time their field was planted (i.e. $\frac{S_C+I_C}{N}$) and "disease prevalence" is the proportion of growers that are currently infected (i.e. $\frac{I_N+I_C}{N}$).

	Disease prevalence	Controller prevalence	Own outcome
Strategy vs population	✓	✓	X
Strategy vs alternative	✓	X	X
Grower vs population	✓	✓	✓
Grower vs alternative	✓	X	✓

the expected profit across the entire population of growers.

- Grower vs. alternative strategy. Growers compare their own outcome during the previous season with the expected profit of the alternative strategy.

A summary of the information required for each means of comparison is provided in Table 2.

The "strategy vs." models are inspired by evolutionary game theory (e.g. [13] and [3]), with Δ depending on the expected profit for the strategy currently adopted. The "grower vs." models are closer to previous usage in plant disease epidemiology (e.g. [6] and [5]), with the outcome obtained by the grower for their last crop used as the point of comparison. Table 3 provides a summary of the symbols used in the behavioural model.

2.5.1 Strategy vs. population

In the "strategy vs." models, the switching probabilities are decoupled from the outcome during the previous season, i.e. $z_{SC} = z_{IC} = z_C$ and $z_{SN} = z_{IN} = z_N$. The probability of a controller switching to no longer use clean seed is

$$z_{SC} = z_{IC} = z_C = \max\left(0, 1 - e^{-\eta(P-P_C)}\right). \tag{34}$$

This probability is only non-zero when the expected profit for a controller is assessed to be smaller than the expected profit over the entire population (i.e. when $P_C < P$). Conversely, the probability

Table 3: Summary of symbols used in defining the behavioural models. The outcomes $i \in \{S, I\}$ represent – at the end of the season – fields that are (S)usceptible and (I)nfected, respectively. The strategies $j \in \{C, N\}$ correspond to (C)ontrollers (i.e. use clean seed) and (N)on-controllers (i.e. do not use clean seed), respectively.

Symbol	Meaning	Defined in
z_{ij}	Switching probability for grower in infection class $i \in \{S, I\}$ who used strategy $j \in \{C, N\}$	Equations 1-4
P_{ij}	Profit for grower with outcome $i \in \{S, I\}$ who used strategy $j \in \{C, N\}$	Equations 9-12
q_j	Estimated probability at time of planting of infection if go onto use strategy $j \in \{C, N\}$	Equations 17 and 24
p_N(Vert)	Estimated probability of vertical infection for non-controllers	Equation 19
p_N(Horiz)	Estimated probability of horizontal infection for non-controllers	Equation 21
P_j	Estimated profit at time of planting if go onto use strategy $j \in \{C, N\}$	Equations 27 and 30
P	Estimated profit at time of planting averaged over entire population	Equation 32
Δ	Estimated difference in expected profit	Equation 33
q_β	Perceived value of the rate of horizontal transmission (β)	Equation 46
q_I	Perceived number of infected fields	Equation 47
q_j^k	Estimated value of q_j for grower k (only differs by grower in the spatial model)	Equations 55 and 56
q_α	Perceived value of the whitefly dispersal scale parameterα	Equations 57
q_{β_S}	Perceived value of horizontal transmission in the stochastic, spatial model (β_S)	Equations 58

of a non-controller switching is

$$z_{SN} = z_{IN} = z_N = \max\left(0, 1 - e^{-\eta(P - P_N)}\right), \tag{35}$$

which takes a non-zero value only when $P_N < P$. Since P is a convex combination of P_C and P_N (the two bracketed terms in Equation 32 are complementary probabilities), only one of P_C or P_N can be greater than P at any time, meaning z_C and z_N cannot simultaneously be non-zero (although if $P_C = P_N$, i.e. the expected profits are equal, then $P = P_C = P_N$ and so $z_C = z_N = 0$).

2.5.2 Strategy vs. alternative strategy

This model is similar to the "strategy vs. population" model, but now instead of comparing to the expected profit of the population, P, growers compare to the expected profit of the alternative

strategy (i.e. P_C or P_N, as appropriate). The probability of a controller switching is

$$z_{SC} = z_{IC} = z_C = \max\left(0, 1 - e^{-\eta(P_N - P_C)}\right), \tag{36}$$

which takes a non-zero value only when $P_N > P_C$, i.e. when non-controllers are expected to be more profitable next season. Conversely, the probability of a non-controller switching is

$$z_{SN} = z_{IN} = z_N = \max\left(0, 1 - e^{-\eta(P_C - P_N)}\right), \tag{37}$$

which is non-zero only when $P_C > P_N$. Here it is clear that only one of z_C and z_N can be non-zero at any time, since either $P_C > P_N$ or $P_C < P_N$ (or $P_C = P_N$, in which case no grower would switch since the expected profits of the two strategies are equal, and $z_C = z_N = 0$).

2.5.3 Grower vs. population

In the "grower vs." models, decisions are made on the basis of grower's own outcome for the last crop, and so all four switching probabilities can differ. The ordering of profits in Equation 13 means that an uninfected non-controller (i.e. class S_N) should never switch strategy, since these "successful free-riders" [27] obtained the highest possible profit for the last crop. Similarly, having obtained the "suckers' pay off", a grower who controlled for disease but was nevertheless infected (i.e. class I_C) will always have a non-zero probability of switching, since they must have obtained a lower than average profit. Whether or not S_C and I_N growers switch strategy depend on their profit relative

to the expectation over the whole population, P. The switching terms are therefore

$$z_{SN} = 0, \tag{38}$$

$$z_{IN} = \max\left(0, 1 - e^{-\eta(P - P_{IN})}\right), \tag{39}$$

$$z_{SC} = \max\left(0, 1 - e^{-\eta(P - P_{SC})}\right), \tag{40}$$

$$z_{IC} = 1 - e^{-\eta(P - P_{IC})}. \tag{41}$$

2.5.4 Grower vs. alternative strategy

This model is similar to the "grower vs. population" model, but now growers compare to the expected profit of the alternative strategy (i.e. P_C or P_N) rather than that of the population, P, with

$$z_{SN} = 0, \tag{42}$$

$$z_{IN} = \max\left(0, 1 - e^{-\eta(P_C - P_{IN})}\right), \tag{43}$$

$$z_{SC} = \max\left(0, 1 - e^{-\eta(P_N - P_{SC})}\right), \tag{44}$$

$$z_{IC} = 1 - e^{-\eta(P_N - P_{IC})}. \tag{45}$$

2.6 Systematic uncertainty

The models as presented thus far presume that all growers can perfectly perceive the risk of infection by knowing the true values of β and the total number of infected individuals (I). However, these quantities must actually be estimated by growers. To account for this, we introduce two new parameters:

$$q_\beta = \nu_\beta \beta, \tag{46}$$

$$q_I = \nu_I (I_N + I_C). \tag{47}$$

Therefore ν_β and ν_I scale the values of β and $(I_C + I_N)$ respectively, amounting to either an over- or underestimate of their values. This modifies their contributions to the estimated profits in Equation 27 and Equation 30, which become:

$$\tilde{P}_C = Y - \phi - L\left(\frac{q_\beta q_I}{q_\beta q_I + \gamma}\right). \tag{48}$$

and

$$\tilde{P}_N = Y - L\left(\frac{pq_I}{N} + \left(\frac{q_\beta q_I}{q_\beta q_I + \gamma}\right)\left(1 - \frac{pq_I}{N}\right)\right). \tag{49}$$

Importantly, these changes to how growers perceive the risk of infection do not change the epidemiology of the system, only the behaviour of the growers. If the value of $\nu_I(I_N + I_C) > N$, we assume that growers estimate that all fields in the system are infected and thus set $\nu_I(I_N + I_C) = N$.

Though the "strategy vs." models are mathematically tractable and therefore allow for the derivation of analytical expressions, it may be unlikely that growers will make their assessment of profitability based on every other grower using the same strategy as they are, rather than just their own outcomes. Thus, to investigate the effect of systematic uncertainty, we used the "grower vs. alternative" model, which has been used in plant epidemiological models that include behaviour [5], [6].

2.7 Spatial, stochastic model

2.8 Host landscape and initial conditions

Disease spreads on a 1,000 Ha square landscape containing $N = 750$ identically-sized fields (a similar density to [6]), placed at uniform random locations. All simulations are started with 75 growers using clean seed (i.e. 10% of the total population), distributed randomly across the landscape. A random

selection of eight non-controllers is set to be infected initially ($1\tilde{\%}$ total population). The location of the growers was kept constant, though the growers that were either initially infected or used the clean seed system differed.

2.9 Dispersal and force of infection

Since horizontal transmission is a consequence of the movement of whitefly, the probability that a particular infected field leads to infection of a susceptible field depends upon the distance d between them. We model this using an exponential dispersal kernel

$$K(d, \alpha) = \frac{1}{2\pi\alpha^2} \exp\left(-d/\alpha\right), \tag{50}$$

in which α is the dispersal scale [28, 29]. This sets the force of infection, Γ_k, upon the field belonging to grower k

$$\Gamma_k = \beta_S \sum_{\substack{l \neq k \\ l \text{ is infected}}} K(d_{\mathrm{k,l}}, \alpha), \tag{51}$$

in which the sum runs over all infected fields (l), $d_{k,l}$ is the distance between fields k and l, and β_S is the effective rate of infection in the spatial version of our model (see “Parameterisation” in main text). The force of infection sets the rate at which grower k becomes infected; it varies in both time and space (since it depends not only on the instantaneous number of infected fields, but also on their spatial arrangement).

2.10 Model dynamics

Each individual grower is characterised by their current strategy as adopted on planting (i.e. controller or non-controller), as well as their current infection status (i.e. susceptible or infected). Between harvesting and replanting events, dynamics are independent of the strategy adopted. We simulate the model using Gillespie’s algorithm [30].

The only event that can affect an infected grower (i.e. class I_C or I_N) is harvesting; this occurs at rate γ. As well as harvesting, susceptible growers (i.e class S_C or S_N) can also become horizontally infected. This occurs, for grower k, at rate Γ_k (Equation 51). As noted above, the force of infection not only depends on time, but also takes different values for each grower.

2.11 Replanting and grower behaviour

Replanting is assumed to occur immediately after harvesting, and potentially leads to changes in both strategy and infection status. Different growers make different assessments of the probability of horizontal infection next season depending on their instantaneous force of infection, with

$$\begin{aligned}\begin{matrix}\text{Estimated probability of}\\ \text{horizontal infection for grower } k\end{matrix} &= \frac{\text{Force of infection on grower } k}{\text{Force of infection on grower } k + \text{Harvesting rate}}, &(52)\\ &= \frac{\Gamma_k}{\Gamma_k+\gamma}, &(53)\end{aligned}$$

We assume growers make decisions according to spatial analogues of Equations 17 and 24

$$\begin{aligned} q_C^k &= \text{Grower } k\text{'s estimate of the probability of infection next season if control is adopted}, &(54)\\ &= \frac{\Gamma_k}{\Gamma_k+\gamma}, &(55)\end{aligned}$$

and

$$\begin{aligned} q_N^k &= \text{Grower } k\text{'s estimate of the probability of infection next season if control is not adopted},\\ &= \frac{p(I_C+I_N)}{N} + \left(\frac{\Gamma_k}{\Gamma_k+\gamma}\right)\left(1-\frac{p(I_C+I_N)}{N}\right). &(56)\end{aligned}$$

All other aspects of the behavioural models follow essentially unchanged, although the switching terms now enter the model via a single Bernoulli trial at the time of planting to determine whether a

grower switches strategy. Whether or not non-controllers become vertically infected is also simulated using a single Bernoulli trial at the time of planting (rather than via a systematic alteration to a rate parameter as in Equations 5-8).

2.12 Accounting for uncertainty in the spatial model

We also investigated misestimation of epidemiological parameters in the spatial model, focusing on the dispersal scale (α) and and the rate of horizontal transmission, β_S:

$$q_\alpha = \nu_\alpha \alpha, \tag{57}$$

$$q_{\beta_S} = \nu_{\beta_S} \beta_S. \tag{58}$$

The exponential dispersal kernel ($\tilde{K}(d, q_\alpha)$) used in growers' decision making is now given by:

$$\tilde{K}(d, q_\alpha) = \frac{1}{2\pi q_\alpha^2} \exp\left(-d/q_\alpha\right), \tag{59}$$

which sets the estimated force of infection, $\tilde{\Gamma}_k$, upon the field belonging to grower k via

$$\tilde{\Gamma}_k = q_{\beta_S} \sum_{\substack{l \neq k \\ l \text{ is infected}}} \tilde{K}(d_{\mathrm{k,l}}, q_\alpha). \tag{60}$$

2.13 Parameterisation

2.13.1 Non spatial model

Baseline parameter values for CBSD were taken from previous studies where possible (Table 1). The rate of secondary infection, β, was set by matching the results of our non-spatial model against [6]. In particular, we used the baseline behaviour of that model to motivate the choice that, in the absence of trade-mediated transmission, with 1% of fields initially infected and with no option of

controlling for disease, 50% of fields would be infected within 10 seasons. To only account for the horizontal transmission, we $p = 0$ when matching these results.

The value of 60% of fields' potential profit lost due to infection was assumed, to account for both the loss of starch content – which causes up to 40% loss of yield [25] – and the reduced market value of infected materials [16].

Vertical transmission requires cuttings taken from infected plants to be replanted and cause infection in the next season. The probability of vertical transmission is therefore reduced by selection, in which growers avoid replanting visibly infected stems [31] and by reversion, in which low viral titres mean healthy cuttings can be taken even from infected plants [32], a process that has been acknowledged for a range of virus diseases [33]. It is difficult to quantify these effects into a single parameter value; we therefore take $p = 0.8$ as a pragmatic but arbitrary default in which vertical transmission at the field scale often, but not always, occurs.

Growers' responses to differences in profit are notoriously difficult to quantify [8], and here we were forced to assume a value. An intuition for our default selection of $\eta = 10$ follows by noting that, using the default costs and losses in the"strategy vs. population" model, a controller will have an approximately 80% chance of switching to become a non-controller if there is sufficient infection in the rest of the system such that it is almost certain they will become infected over the next season (i.e. if $q_C \approx 1$).

2.13.2 Spatial model

The majority of parameters in the spatial model took the same values to those in the simpler non-spatial model (Table 1). However, following [6] and based on dispersal of Bemisia tabaci reported by [34] and [26], we set the scale parameter of our dispersal kernel to be $\alpha = 150$ m. The move to a spatially-explicit model required a re-scaling of the rate of secondary infection, which we set to $\beta = 110$ day^{-1} field^{-1}. This value was again chosen to ensure that, after 10 seasons, on average 50% of fields would be infected.

3 Results

3.1 Non-spatial model

Due to its mathematical tractability, our initial investigations were focused on the "strategy vs. population" model. However, when considering the effect of systematic uncertainty, and later the spatial model, we focus on the "grower vs. alternative" model.

3.1.1 Probability of infection, responsiveness, profits, and switching terms

To demonstrate the underlying drivers of dynamics in our models, we dissect the behaviour of the "strategy vs. population" model in some depth. We first investigated the response of the probability of infection, the expected profits and the probability of switching strategy to the total number of infected fields (Figure 2). Generally, as the number of infected fields increases, so too does the probability of infection for controllers and non-controllers (q_C and q_N respectively) (Figure 2(A)). However, the increased number of infected fields changes the relative importance of the infection pathways for non-controllers. With an increase in the number of infected fields, the instantaneous probability of horizontal transmission for non-controllers (p_N(Horiz), Equation 21), decreases due to the increased vertical transmission (p_N(Vert), Equation 19) (Figure 2(A)).

For the default parameterisation (Figure 2(B)), the expected profits for the non-controllers (P_N) are always higher than that of the population, so they should always have a zero probability of switching strategy (Figure 2(C)). Conversely, P_C is always below the population average, and thus they should always have a non-zero probability of switching. This results in an equilibrium where there are no controllers present, as all of them have abandoned using the unprofitable CSS. However, by reducing the cost of clean seed ($\phi = 0.125$, equivalent to a 50% subsidy), at around $I = 411$ fields control becomes more profitable and non-controllers have a non-zero probability of switching strategy (Figure 2(E)). As at this point controllers should not switch strategy, is an "all control" equilibrium. The point at which $P_N = P_C$ (i.e. when $I \approx 411$, which is the total number of infected fields in

the system and does not describe the number of infected controllers and non-controllers) leads to an equilibrium where both control strategies are present.

The value of η has no effect on the equilibrium attained by the growers in the "strategy vs." models, as the equilibrium is based only on the relative values of P_C, P_N and P, though it did affect the time it took to reach equilibrium (Figure 2(D)). This is in contrast to the "grower vs." models, where the two-strategy equilibrium is reached when the flow of growers out of one strategy matches the flow of growers out of the other (i.e. $z_{SC}S_C + z_{IC}I_C = z_{IN}I_N$). As the switching terms are set by the value of η, there is a dependence in the final equilibrium on the value of this parameter (Appendix 2 Figure 2).

3.1.2 Baseline results

Using the default parameters, we obtain a "no control" equilibrium when growers employ the "strategy vs." model (Figure 3(A)-(B)). At these parameter values, the rate of horizontal transmission and probability of vertical infection are so high that growers are likely to become infected, thus paying the cost of control (ϕ) and the loss due to disease (L). Therefore, control is not worthwhile. For the default parameters, both "strategy vs." models have similar dynamics. Providing growers with a 50% subsidy for clean seed use (i.e. reducing ϕ to 0.125) was sufficient to encourage control at equilibrium (Figure 3(E)-(F)) and introduces differences in early epidemic dynamics.

For the "grower vs. population" model, around 38% of growers control at equilibrium. For these parameters, any infected non-controlling grower (I_N) considers changing strategy as the presence of uninfected non-controllers (S_N) means that they will earn below the expected population profit, P (see Equation 39). Thus, there will be controllers at equilibrium. Only when the parameter values mean that there will be no susceptible growers will there be a "no control" equilibrium (Equation 78).

This is not observed in the "grower vs. alternative" model, where no growers control at equilibrium (Figure 3(D)). For this model, infected non-controlling growers (I_N) need only earn more than

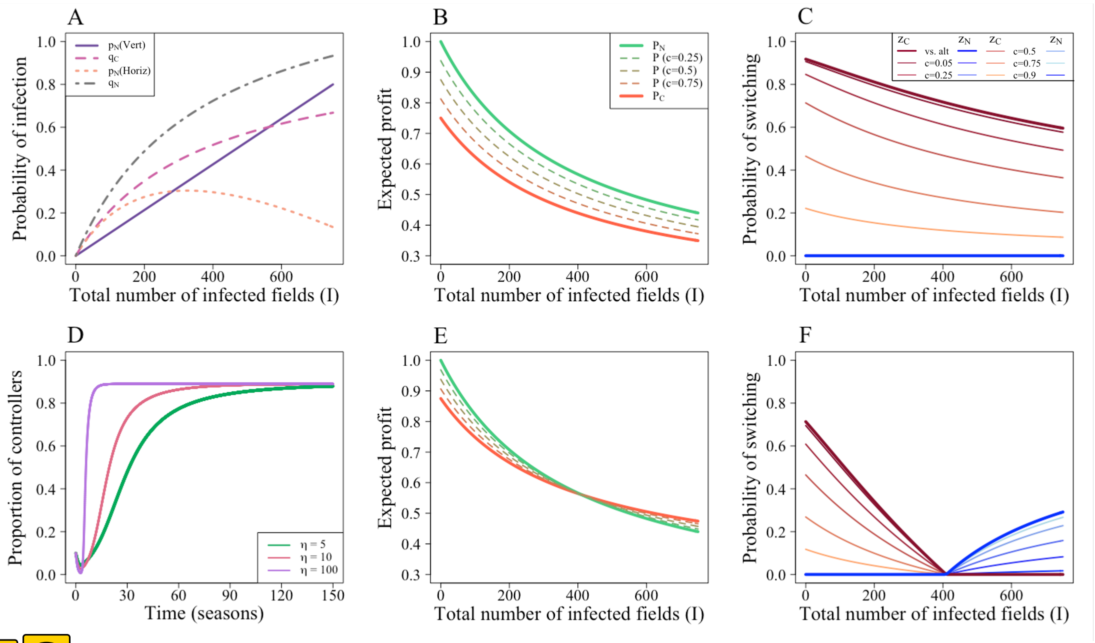

Figure 2: Probability of infection, responsiveness of growers, expected profits, and switching terms for the "strategy vs. population" model. (A) For the default parameterisation, the probability of infection increases as the number of infected growers increase, though the probability non-controllers will be infected via horizontal transmission falls as I increases. (B) The expected profits for controllers, non-controllers and the population for different proportions of controllers in the population (c). For the default parameters, $P_C < P$ for all values of I. (C) Switching probabilities for controllers and non-controllers for different c. As $P_C < P$, controllers should always have a non-zero probability of switching strategy, whereas non-controllers should never start to control. (D) The responsiveness of growers (η) does not affect the final equilibrium values, though it does impact the time it takes to reach equilibrium. ~~Note, however, for the "grower vs." models, the value of η does affect the final equilibrium values (see Appendix 2)~~ (E) shows the expected profits for controllers, non-controllers and the population ~~for ϕ~~ $= 0.125$. Now, at high levels of I, it is profitable to control. Where $P_C = P_N$, both strategies will be present at equilibrium. (F) For $\phi = 0.125$, for low values of I controllers should switch strategy, but as I increases non-controllers should have a non-zero probability of switching into the clean seed system. For this parameter set, the equilibrium value of I is therefore 411, with 349 infected controllers and 63 infected non-controllers.

the alternative strategy, so if there is a high number of infected controllers I_N should not switch strategy (Equation 77).

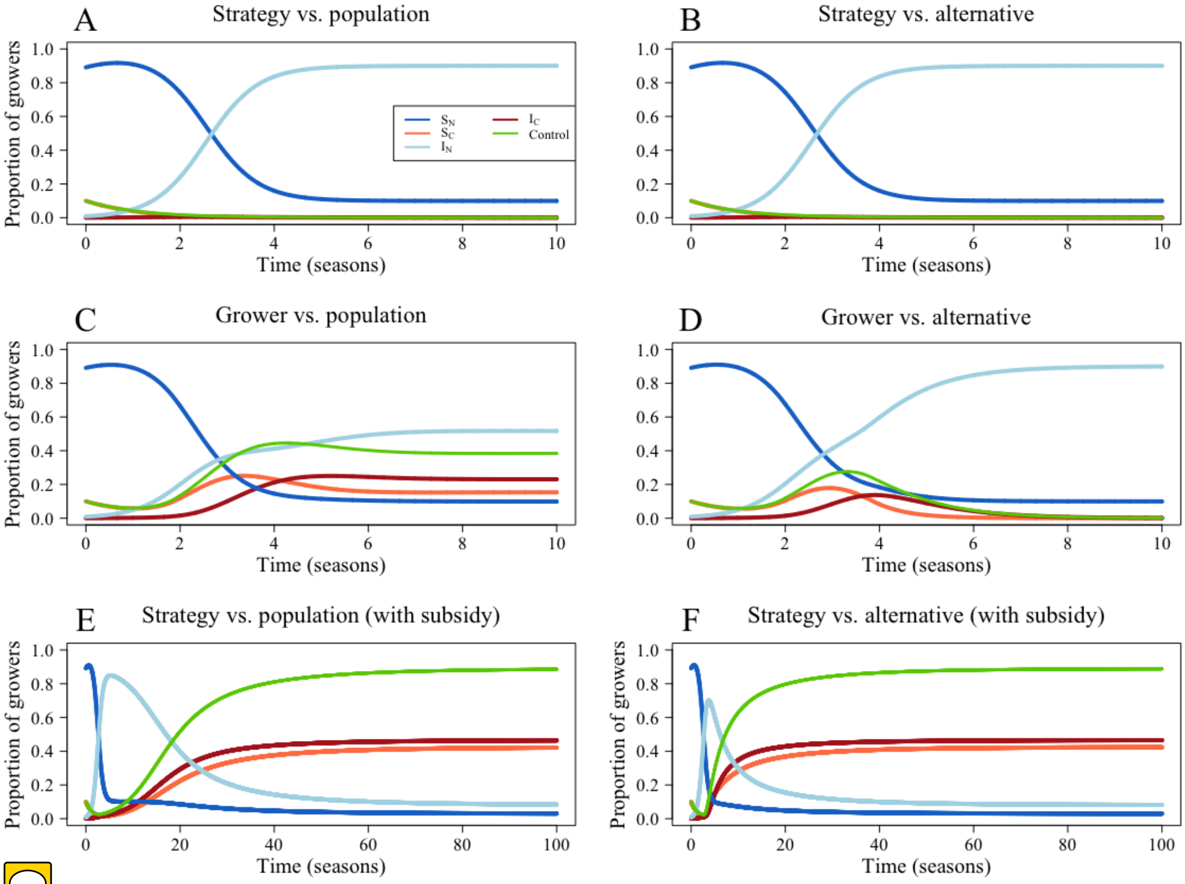

Figure 3: Model dynamics for different means of comparison. In (A) and (E), growers compare the expected profit of their strategy with that of the entire population; in (B) and (F) they compare the expected profit of their strategy with the expected profit of the alternative strategy. For (C), growers compare their own outcome with expected profit of the population and in (D) they compare their outcome with the expected value of the alternative strategy. The first four panels use the default parameters; in (A), (B) and (D), no growers control for disease, though in (B) around 38% of growers control. Default parameters are outlined in Table 1. For (E) and (F), a lower value of $\phi = 0.125$ (equivalent to a 50% subsidy on the cost of clean seed) was used, which lead to growers controlling at equilibrium. The subsidy extended the amount of time it took to reach equilibrium, so we ran the models for 100 seasons.

3.1.3 Characterising model equilibria

The pathogen's basic reproduction number in the absence of control (Appendix 1) is

$$R_0 = \text{Basic reproduction number} = R_0^H + R_0^V = \frac{\beta N}{\gamma} + p, \tag{61}$$

with distinct components corresponding to horizontal (R_0^H) and vertical (R_0^V) transmission ([35]; Appendix 1).

Strategy vs." models. The coupling of the switching terms in these formulations allows for mathematical analysis (Appendix 1). The equilibrium attained is uniquely specified by parameters; assuming the pathogen is present in the system at $t = 0$, there is no other dependence on initial conditions. As noted above, although the dynamics in reaching equilibrium can differ, the final state of the system does not depend on whether the "strategy vs. population" or "strategy vs. alternative strategy" model is considered. In each case, for an equilibrium with both strategies present, the expected payoffs (P_C and P_N) must be equal, otherwise one group of growers will continue to be incentivised to change strategy. For only controllers to be present, $P_C > P_N$ and vice versa for the non-controller equilibria. The differences in dynamics approaching equilibrium arise from the different calculations of Δ (Equations 34 - 37).

There are four equilibria, which can be distinguished by the presence of disease and the proportion of growers controlling at equilibrium.

- Disease-free equilibrium at which the disease is not able to spread even when there is no control via clean seed

$$(S_C, I_C, S_N, I_N) = (0, 0, N, 0). \tag{62}$$

  This equilibrium is stable whenever $R_0 < 1$ (and at this equilibrium no growers control for disease, since it is never rational to do so).

- Control-free, disease-endemic equilibrium at which the disease is able to spread in the absence of control, but nevertheless no grower controls

$$(S_C, I_C, S_N, I_N) = \left(0, 0, \frac{\gamma(1-p)}{\beta}, \frac{\beta N - \gamma(1-p)}{\beta}\right). \tag{63}$$

As there are no controllers at this equilibrium, $P_N = P$ (Equation 32). This equilibrium requires $R_0 > 1$, and is stable whenever:

$$Y - L\left(\frac{p(\beta N - \gamma(1-p))}{\beta N} + \frac{\beta N - \gamma(1-p)}{\beta N + \gamma p}\left(\frac{\beta N - p(\beta N - \gamma(1-p))}{\beta N}\right)\right) > Y - \phi - L\left(\frac{\beta N - \gamma(1-p)}{\beta N + \gamma p}\right) \tag{64}$$

This can be simplified to:

$$\begin{matrix}\text{Loss due to}\\ \text{disease}\end{matrix} \times \begin{matrix}\text{Probability of}\\ \text{vertical transmission}\end{matrix} \times \begin{matrix}\text{Proportion of}\\ \text{infected fields}\end{matrix} \times \left(1 - \begin{matrix}\text{Probability of}\\ \text{horizontal transmission.}\end{matrix}\right) < \begin{matrix}\text{Cost of}\\ \text{control,}\end{matrix}$$

~~(65)~~

$$Lp\left(\frac{\beta N - \gamma(1-p)}{\beta N}\right)\left(1 - \frac{\beta N - \gamma(1-p)}{\beta N + \gamma p}\right) < \phi. \tag{66}$$

This inequality shows that the control-free equilibrium exists and is stable when the expected cost of losses due to vertical transmission is less than the cost of control.

- All-control, disease-endemic equilibrium at which all growers control, but nevertheless disease is still present in the system

$$(S_C, I_C, S_N, I_N) = \left(\frac{\gamma}{\beta}, \frac{\beta N - \gamma}{\beta}, 0, 0\right) \tag{67}$$

Similarly to the previous equilibrium, as there are no non-controllers at this equilibrium, $P_C = P$ (Equation 32).

This is stable whenever $R_0^H > 1$ (i.e. horizontal transmission alone is sufficient for disease to

persist) and when the expected profit of controllers is greater than that of non-controllers for these equilibrium values:

$$Y - L\left(\frac{p(\beta N-\gamma)}{\beta N} + \frac{\beta N-\gamma}{\beta N}\left(\frac{\beta N - p(\beta N-\gamma)}{\beta N}\right)\right) < Y - \phi - L\left(\frac{\beta N-\gamma}{\beta N}\right) \tag{68}$$

Again, this can be simplified in terms of the expected losses and costs of control:

$$\begin{matrix}\text{Cost of} \\ \text{control}\end{matrix} < \begin{matrix}\text{Loss due to} \\ \text{disease}\end{matrix} \times \begin{matrix}\text{Probability of} \\ \text{vertical transmission}\end{matrix} \times \begin{matrix}\text{Proportion of} \\ \text{infected fields}\end{matrix} \times \left(1 - \begin{matrix}\text{Probability of} \\ \text{horizontal transmission}\end{matrix}\right),$$

~~(69)~~

$$\phi < Lp\left(\frac{\beta N-\gamma}{\beta N}\right)\left(1-\frac{\beta N-\gamma}{\beta N}\right), \tag{70}$$

i.e. the cost of control is less than the potential losses due to vertical transmission.

- Two-strategy, disease-endemic equilibrium at which both disease and control equilibrate at some intermediate level, with

$$S_C = \frac{N(R_0(\gamma pL - \beta\phi N) - \gamma pL)}{\gamma p^2 L}, \tag{71}$$

$$I_C = \frac{\beta\phi N^2(R_0(\beta\phi N - \gamma pL) + \gamma pL)}{\gamma p^2 L(\beta\phi N - \gamma pL)}, \tag{72}$$

$$S_N = \frac{N(\phi R_0 - pL)(\beta N(\beta\phi N - \gamma pL) + \gamma^2 pL)}{\gamma p^2 L(\beta\phi N - \gamma pL)}, \tag{73}$$

$$I_N = \frac{-\phi N R_0(\beta N(\beta\phi N - \gamma Lp) + \gamma^2 Lp)}{\gamma p^2 L(\beta\phi N - \gamma Lp)}. \tag{74}$$

This requires $R_0 > 1$ and it is stable whenever both of the simpler disease-present equilibria are not stable (i.e. neither Equation 66 or 70 is satisfied; Appendix 1).

Parameterisations leading to each of the four equilibria are possible, depending on the balance between the rate of horizontal transmission (β) and the cost of control (ϕ) (Figure 4(A)). This includes the "all control" equilibrium, which is due to the imperfect protection provided by the

control scheme; if horizontal infection is sufficiently significant, even all growers controlling it not guara[illegible] the system remains disease-free.

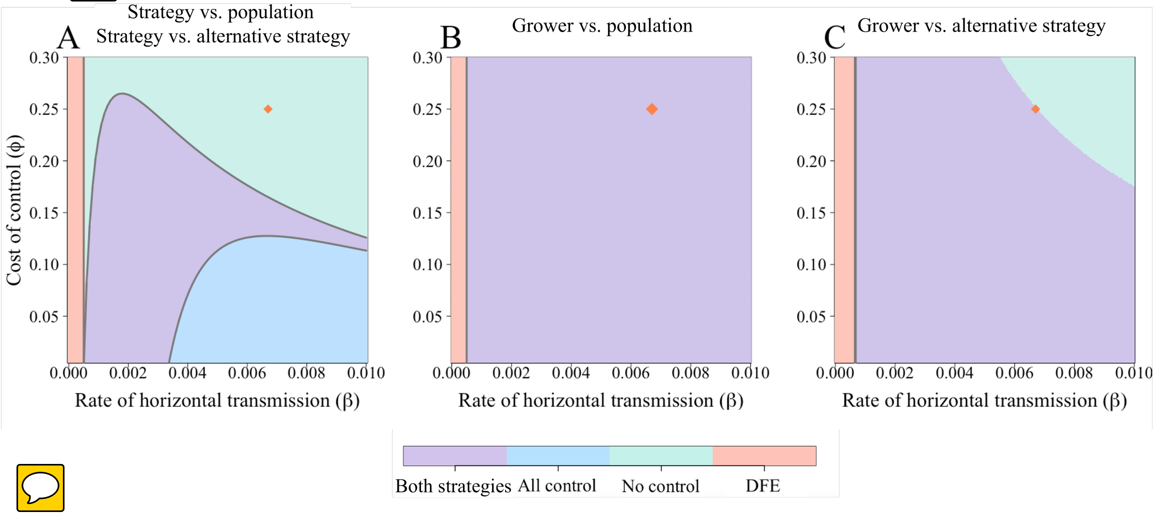

Figure 4: Effect of parameters on final equilibrium attained. (A) The "strategy vs. population" model has four possible, mutually-exclusive equilibria. Which is attained depends both on R_0 and the balance of benefit between controlling and not controlling. Boundaries between equilibria are determined by the stability conditions (Equations 66 and 70). Also shown in (B) and (C) are the equilibria for the "grower vs." models, though numerical expressions determining these cannot be derived analytically. Importantly, (B) does not show all equilibria achievable for the "grower vs. population" model, as it omits the "no control" equilibrium, which can only be attained under a narrow range of parameter conditions that cause all non-controllers to be infected at equilibrium. The orange diamonds mark the default values.

Grower vs." models. The more complex form of these models makes mathematical analysis difficult, as conditions for model equilibria no longer simply depend on the difference in profits between controllers and non-controllers. However, extensive numerical work (Appendix 1) indicates the equilibrium that is reached again depends only on model parameters. For these "grower vs." models to reach a two-strategy equilibrium, the flow between the two strategies must be equal, i.e.

$$z_{IN} I_N = z_{SC} S_C + z_{IC} I_C \tag{75}$$

The forms of the switching terms will differ between the two "grower vs." models, leading to different equilibria for each model (Figure 3(C)-(D)). There are two important differences from the

"strategy vs." models:

- The "all control" equilibrium is impossible because growers who controlled but nevertheless became infected always consider switching strategy, as they are earning the lowest possible payoff (P_{IC}, the "sucker's payoff"). As long as there is a non-zero probability of infection (which is necessary for control to be worthwhile) there will always be non-controlling growers.

- For the "grower vs. population" model, the "no control" equilibrium is only possible if all non-controllers are infected at equilibrium. For a "no control" equilibrium, the expected profit of the population must be less than or equal to P_{IN} to prevent I_N growers switching strategies (i.e. $P \leq P_{IN}$, Equation 43). At the "no control" equilibrium $P = P_N$ (Equation 32), leading to:

$$P = \frac{P_N(S_N + I_N)}{N} \leq P_{IN} \tag{76}$$

$$\tag{77}$$

As, in the "no control" equilibrium, $S_N + I_N = N$, this can be simplified:

$$P_N \leq P_{IN} \tag{78}$$

$$q_N P_{IN} + (1 - q_N)P_{SN} \leq P_{IN} \tag{79}$$

$$(1 - q_N)P_{SN} \leq P_{IN}(1 - q_N) \tag{80}$$

$$P_{SN} \leq P_{IN} \tag{81}$$

As this is impossible (Equation 9 & 10), it means that there must be no S_N present for a "no control" equilibrium (i.e. $q_N = 1$). In Figure 4 (B), a "no control" equilibrium would require higher β and p (Appendix 2 Figure 3).

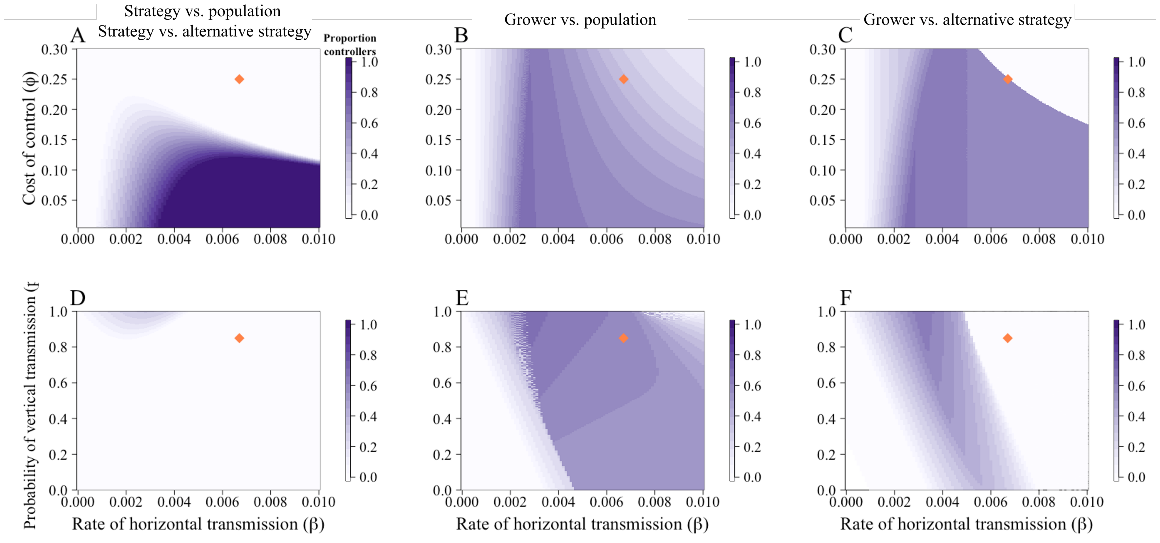

Figure 5: ~~Impact of~~ epidemiological and economic parameters ~~on CSS use~~. Plots show the equilibrium proportion of controllers in response to variations in parameters when comparisons are made via the strategy vs. population or alternative ((A) and (D)), grower vs. population ((B) and (E)) or grower vs. strategy ((C) and (F)) means of comparison. The orange diamonds mark the default values. (A)-(C) Effect of the rate of horizontal transmission (β) and cost of control (ϕ). At low values of β, controlling is worthwhile, but as infection becomes more likely, the value of control diminishes so it is not worthwhile to invest in control. (D)-(F) Effect of the rate of horizontal transmission (β) and probability of receiving infected planting material via trade (p). At these default parameter values (particularly the high cost of control), the probability of receiving infected material has little effect on uptake of control.

3.1.4 Effect of epidemiological and economic parameters on uptake of control

Responses were often intuitive (Figure 5). For example, an increased loss due to disease (L) led to more growers controlling, particularly at higher rates of vertical transmission (p) (not shown). However, there were also non-monotonic responses. We found that at medium-to-low values for cost of control (ϕ) and the rate of horizontal infection (β), high proportions of growers controlled for disease (Figure 5(A)-(C)). When β increased, the higher probability of infection means that control is too costly, as the grower will pay both the cost of control (ϕ) and the loss due to disease (L) (the "sucker's payoff").

A similar principle underpins Figure 5(E)-(F). At higher probabilities of vertical infection, disease

pressure increases and controllers are likely to be infected. For Figure 5(D), the high default cost of control (ϕ) means low levels of control are seen irrespective of the value of p; if ϕ was subsidised, the proportion of controllers would increase (Appendix 2 Figure 4).

In all cases, where there were higher proportions of controllers in the population, there were lower prevalences of infection and, generally, an increase in the average profit and yield attained by the growers (Appendix 2 Figure 5).

3.1.5 Effect of systematic uncertainty

Using the "grower vs. alternative" model, we investigated the effect of all growers misestimating epidemiological parameters relating to the spread and prevalence of disease. We focused on this model formulation as it only requires growers to misestimate the profitability of the alternative strategy. We investigated behaviour at two values of ϕ: the default parameter value of $\phi = 0.25$ (Figure 6(A) and (B)) or a value equating to a 50% subsidy ($\phi = 0.125$; Figure 6(C) and (D)).

Even when growers underestimate β such that they do not believe there to be any risk of horizontal transmission (i.e. $\nu_\beta = 0$, so $q_\beta = 0$), some still use the CSS as the probability of vertical transmission is non-zero (Figure 6(A) and (C)). As ν_β increases, fewer growers use the control scheme as they perceive they will achieve the "sucker's payoff". The default parameterisation (when $q_\beta = \beta$, i.e. $\nu_\beta = 1$) means that growers should never control. The higher cost of ϕ caused the system to reach a "no control" equilibrium Figure 6(A)), though with higher values of ν_β ($\nu_\beta > 2$) this would also be attained for $\phi = 0.125$.

When ν_I is small and increasing, there are more controllers at equilibrium (Figure 6(B) and (D)). At low values of ν_I, the non-infected controllers (S_C) growers perceive that $\tilde{P}_N > P_{SC}$, so they should still consider switching strategy (i.e. $z_{SC} > 0$) (Figure 6(E)). As ν_I increases, their perception of the profit, $\tilde{P}_N$ (Equation 49) falls and fewer S_C growers switch. This causes an increase in infected controllers (I_C), fewer S_C switch strategy before they become infected. Eventually, $\tilde{P}_N < P_{SC}$ and S_C growers stop switching strategy entirely (around season 4 in Figure 6(F)). Many of these

controllers will then become infected and switch strategy, leading to lower participation in control with higher ν_I. As ν_I increases, I_N growers are less likely to switch strategy as $\tilde{P}_C$ falls. Eventually, $z_{IN} = 0$ and no I_N growers should switch strategy (Figure 6(H)). As all I_C growers should have a non-zero probability of switching strategy, but no non-controllers should switch, this leads to a "no control" equilibrium when $\phi = 0.25$.

The subsidy means that even when ν_I is high, there will still be controllers at equilibrium. Unlike when misestimating β, misestimations of I affect perception of both horizontal and vertical transmission. The increased perception of vertical transmission combined with the lower ϕ is enough to encourage control at equilibrium.

3.2 Spatial, stochastic model

3.2.1 Underlying dynamics

In constructing our spatial model, we again focused on the "grower vs. alternative" comparison. Under the default parameterisation, no grower controls (Figure 7(A)), though providing a 50% subsidy encouraged participation in the CSS (Figure 7(B)). Disease spreads rapidly between fields, both due to the high values of β and ϕ (which discourages control) and the non-spatial aspect of trade, which allows disease to spread far away from its initial source.

The results of our spatial model are comparable with the deterministic version of the model (Figure 8). For equivalent rates of horizontal transmission, the equilibrium values for the deterministic model are similar to the values attained after 50 seasons in the spatial, stochastic model ((Figure 8)(A) and (B)). As in Figure 6, the kinks in these graphs are a result of susceptible controllers (S_C) growers ceasing to switch strategy (around $\beta_s = 50$ and $\beta = 0.003$ in 8(A) and (B)). As the infection pressure is increasing, these susceptible growers then become infected and switch into the non-control strategy, causing an overall decrease in controllers.

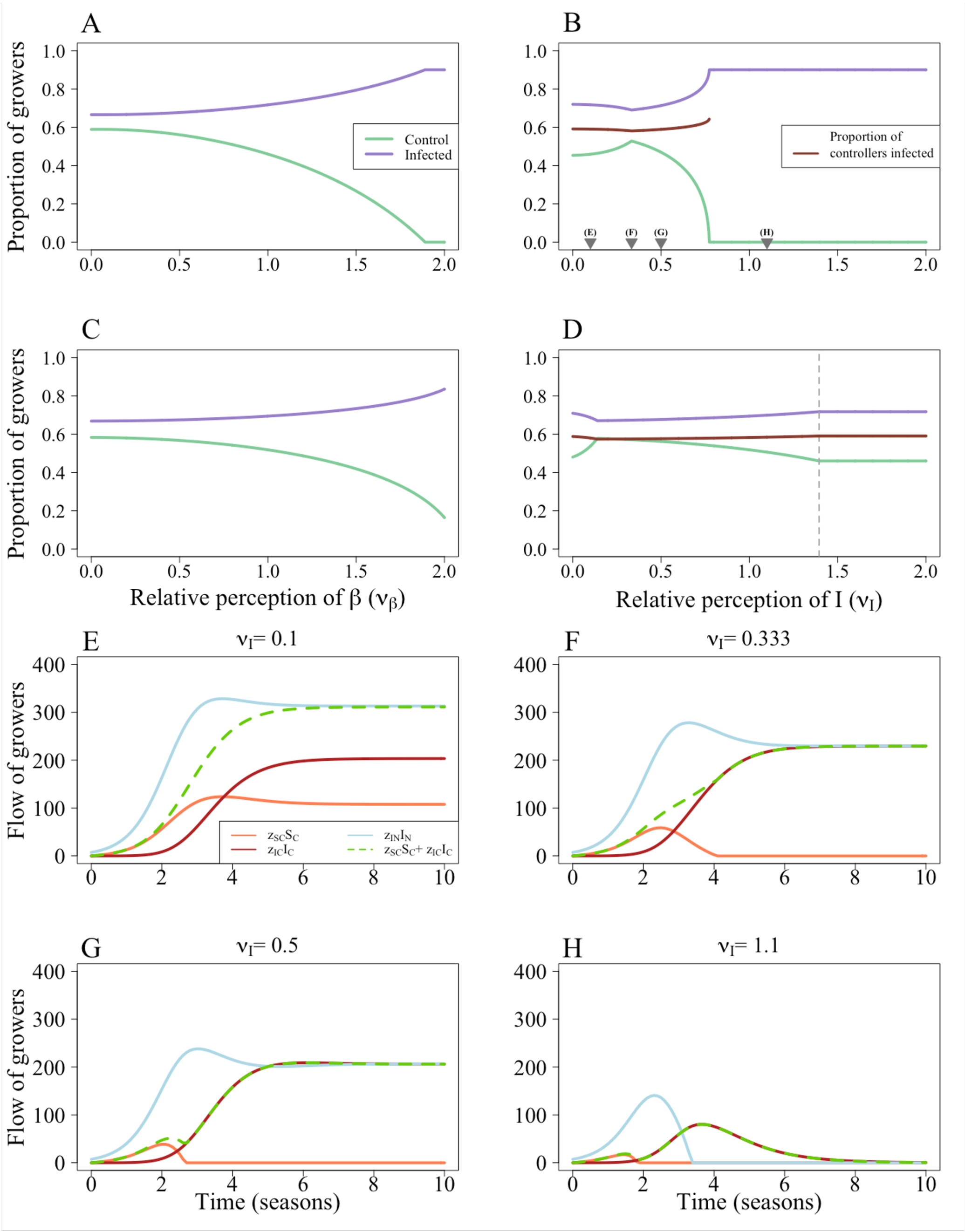

Figure 6: Effect of systematic uncertainty of epidemiological parameters and quantities. (A)-(B) are for the default parameters with $\phi = 0.25$, whereas (C)-(D) are for $\phi = 0.125$. In both cases, when ν_β is small there is a higher proportion of controllers ((A) and (C)), which decreases as ν_β increases. In (B) and (D), there is initially an increase in the proportion controlling with ν_I. In (B) the number of infected fields levels out once the conditions for a "no control" equilibrium are met. In (D) the number of infected fields stops changing when $q_I > N$, at which point the growers assume that all fields are infected (denoted by the vertical dashed line). The grey triangles in (B) show the values of ν_I used in (E)-(H). (E) At low values of ν_I, S_C growers still perceive that it is profitable to change strategy ($z_{SC} > 0$). (F) At higher values of ν_I, as the epidemic progresses and the number of infected fields increases, the perceived value of P_N (i.e. $\tilde{P_N}$) decreases and $z_{SC} = 0$. However, they continue to get infected and there is an increase in I_C fields. (G) At higher values of ν_I, S_C fields stop switching strategy earlier in t[illegible]pidemic. Additionally, as $\tilde{P}_C$ falls, so does z_{IN}. (H) Eventually, at very high values of ν_I, $\tilde{P}_C$ is below that of $\tilde{P}_{IN}$, so $z_{I_N} = 0$. This leads to a "no control" equilibrium.

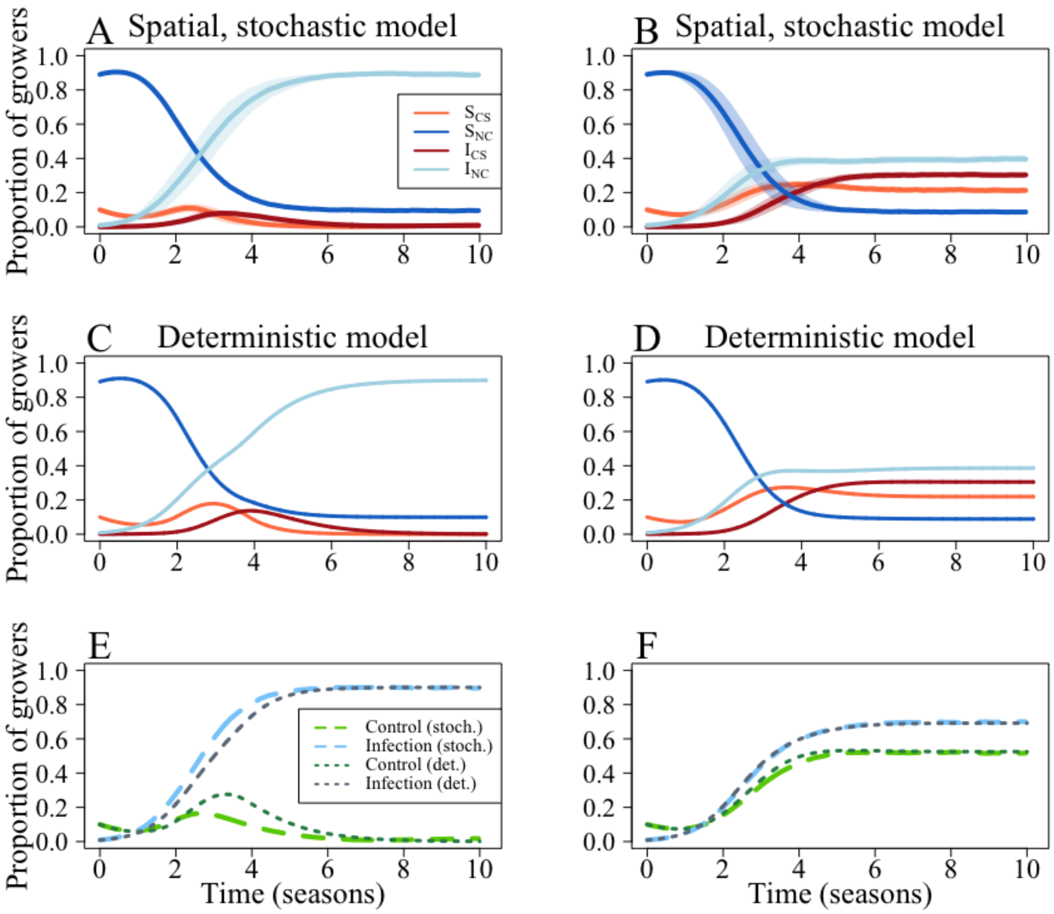

Figure 7: Comparison of default behaviour of the "grower vs. alternative" spatial, stochastic and deterministic models. (A) Dynamics for the grower vs. alternative spatial model. As with the deterministic model (B), under the default parameterisation no growers use the CSS after 10 seasons. Adding a subsidy in (C) and (D) allows for the two-strategy equilibrium. The figures show the mean for 100 runs of each model, and the error bars show one standard deviation. The equilibrium values and dynamics for spatial ((A) and (C)) and non-spatial models ((B) and (D)) are very similar in both cases, emphasised in (E) and (F), which show the prportions controlling and infected for the spatial, stochastic ("stoch.") and deterministic ("det.") models.

In both cases, when ϕ is varied, at around $\phi = 0.19$, growers should stop using the control strategy (Figure 8(C) and (D)).

3.2.2 Effect of systematic uncertainty

Growers misestimating the dispersal scale for the whitefly vector (α) has a similar effect to when growers misestimated β in the deterministic model. As the perceived value of α (q_α, given by

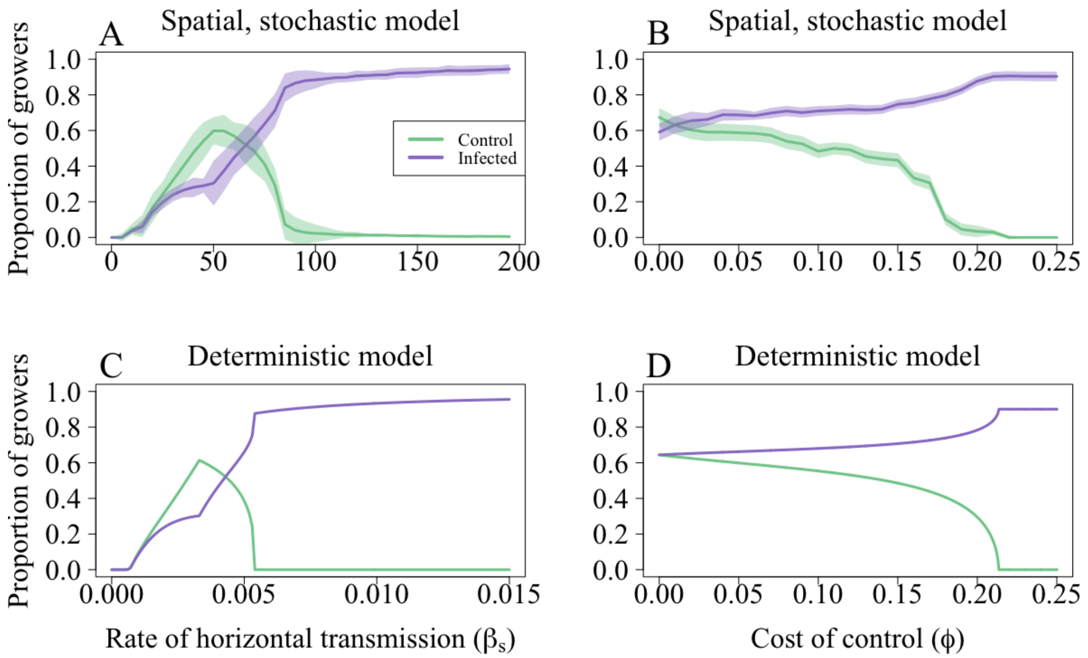

Figure 8: Response to changes in the rate of horizontal transmission and cost of control for the "grower vs. alternative" models. (A) and (C) The proportion of controllers and infected fields after 50 seasons for the spatial, stochastic model and (B) and (D) the equilibrium values for the deterministic model. Aside from the parameters being scanned over, the default parameters are used. Results are broadly similar between the two models, with differences arising from the speed of dynamics. In the spatial models, the means over 100 runs (excluding runs where disease went extinct, which occurred in each simulation when $\beta_S = 0$, and around 40 % of the time when $\beta_S = 40$, but above that value of β_S was very rare) and the error bars show one standard deviation around the mean.

$q_\alpha = \nu_\alpha \alpha$, Table 1) increases, fewer growers participate in the CSS as they estimated that they would be paying the dual penalty of the cost of control and loss due to disease (Figure 9).

When growers misestimate the rate of horizontal transmission, β_S, the pattern of CSS use again resembles that of the deterministic model (Figure 6(A)). At low values of the perceived value of β (q_{β_S}, with $q_{\beta_S} = \nu_{\beta_S}\beta_S$, Table 1), more growers use the control scheme as they believe that they are unlikely to be infected. As q_{β_S} increases, growers believe that they are likely to pay the dual penalty of the cost of control, ϕ, and the loss due to infection, L, and therefore abandon the CSS.

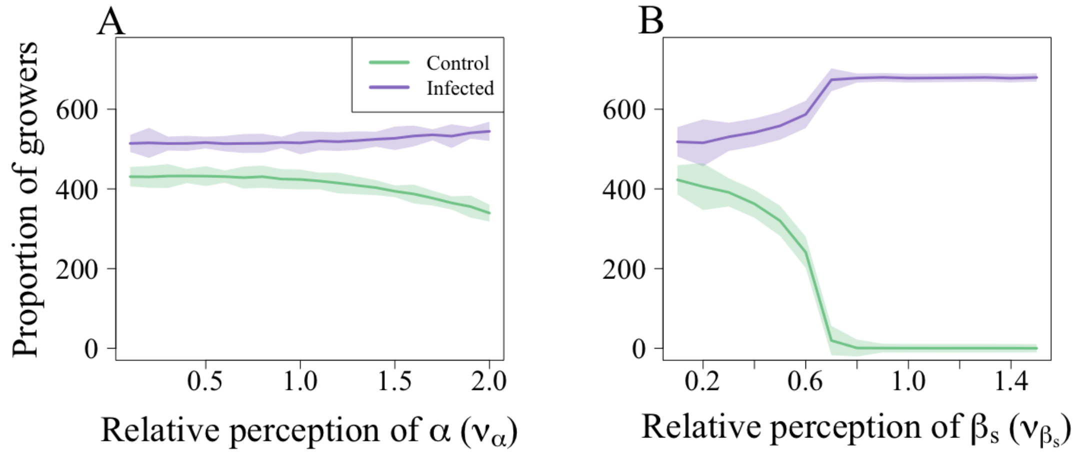

Figure 9: Effect of systematic misestimation in the spatial, stochastic model. (A) For the default parameters, as the perceptions of the dispersal scale for the whitefly vector (ν_α) increase, fewer growers use the control scheme as they estimate that they would likely end up infected. (B) As perceptions of the rate of horizontal transmission increase (ν_β), fewer growers use the CSS in a pattern that matches that seen in Figure 6(A). The mean values were calculated over 100 runs are the error bars show one standard deviation around the mean.

4 Discussion

Surprisingly few plant disease studies examine how behaviour alters the dynamics of epidemic models. Here we presented a model that integrates grower decision making into a simple model for CBSD and investigated the effect of economically- and epidemiologically- important parameters on disease spread.

We found that, save for the two "strategy vs." models that had the same equilibrium values, grower participation in the clean seed system (CSS) depended on what behaviour model the growers used. All models had different dynamics approaching equilibrium. Thus, while the "strategy vs." models are interchangeable in terms of the long-term outcomes, they have different outcomes for growers in the short term. Additionally, only the "strategy vs." comparisons permitted an "all control" equilibrium, as the "sucker's payoff" achieved by infected controllers means that they will always switch strategy in the "grower vs." comparisons. Therefore, it is important not only to include behaviour in epidemic models, but to consider how it is included based on what quantities

are being compared and what information will be available to individual growers.

Game theory predicts that high participation in voluntary disease control schemes is difficult to attain, as the success of disease control policies in reducing the probability of infection disincentiveses continued use of the scheme ([11], [36], [13]; reviewed in [2]). Indeed, in the case of voluntary vaccinations, at least with perfect immunity, models predict that actual level of vaccination will be less than that needed for herd immunity [1]. Yet if vaccines are not completely effective, the reduction in magnitude of positive externalities can lead to increased vaccine uptake until a stable "all vaccinated" equilibrium is attained [37]. That is, as the vaccine no longer confers perfect immunity to the vaccinated, they may then transmit the disease to the unvaccinated. This decreases the benefit of high levels of vaccination in the population to the unvaccinated, and thus encourages them to be engage in disease prevention themselves.

In our model, it was the imperfect protection offered by the CSS that allowed the "all control" equilibrium to be attained in the "strategy vs." models (Figure 4(A)). Although the CSS eliminates a grower's chance of receiving infected material via vertical transmission, it offers no protection from infection via the whitefly vector. By comparing the average payoff of everyone using their strategy, rather than basing decisions on their own outcomes, even growers who themselves received the lowest possible payoff do not necessarily have a positive probability of switching strategy. The "all control" equilibrium was not possible for the "grower vs." models as recipients of the "sucker's payoff" (i.e. infected controllers) will always consider switching strategy.

We found that use of CSS could reduce CBSD spread, but never lead to disease elimination (Equation 63). By considering the stability of the equilibria, we can see that for an "all control" equilibrium, disease must be capable of spreading via the whitefly alone vector (i.e. the horizontal-transmission $R_0^H > 1$, Appendix 1). Fields that were initially planted with clean seed can therefore become infected, allowing disease to remain endemic in the system. Thus, though increased use of the CSS leads to lower disease prevalence, some degree of disease spread is required for growers to consider the CSS worthwhile.

When constructing the spatial, stochastic model, we focused on the "grower vs. alternative" evaluation, where individual growers compare their own profits with the expected profit of the alternative strategy. The reasoning behind this was twofold: first, this has been used previously in plant epidemiological literature [5], [6]. Secondly, and most importantly, we believe this comparison to be most similar to what real growers might be expected to do. It requires a moderate amount of information, as it only relies on the individual knowing their own profit from the previous season and the expected profit of the alternative strategy (Table 2). The results for this model were comparable with those for the equivalent deterministic model (Figure 8), demonstrating the robustness of our results to stochastic and spatial effects. This was in part because growers' of the non-spatial aspect of trade, as it allowed for transmission across the relatively small landscape considered here [20]. When trade was not included (i.e. $p = 0$) the disease spread across the landscape in a more "wavelike" pattern as expected from the thin-tailed dispersal kernel for the whitefly ([38], Appendix 2 Figure 6). However, when trade occurs via a market or central organisation the majority of transactions occur over a scale larger than our landscape (a square with sides 3.16 km), with around 70 % of transactions occur over a scale of 10-50 km [7]. For more informal trade settings, with growers interacting with each other, a proximity-based kernel may be more appropriate ([20], [6], though both of these were modelled over a larger landscape than ours). However, such exchanges are hard to parameterise as the probability of exchanging planting material is highly variable across settings (varying between 30 - 92.51% over unspecified spatial scales; [39], [40], [41], [42] and [43]).

We assumed that all growers had the same responsiveness (our parameter η). This impacted the final equilibrium value attained in our "grower vs." models (Appendix 2 Figure 1), but not our "strategy vs." models (Figure 2). We have also assumed that growers would always choose the most profitable strategy; if the alternative strategy has a higher expected profit, growers have a non-zero probability of switching (Figure 1(C)). As η increases, growers are almost guaranteed to switch strategy, which approaches the assumption of perfect rationality in game theory [12]. Both rationality and responsiveness are likely to vary from grower to grower. Their distribution can be

described by a “risk attitude” framework, in which individuals fall on a spectrum between risk tolerant and risk averse [44]. Including such heterogeneity in risk perception in future studies would allow for greater understanding of behaviour in uncertain environments.

We have only considered economic incentives for grower participation in the CSS. Realistically, other considerations - such as preference for local varieties, flavour, etc. - will play an important role in determining participation. Indeed, surveys of cassava growers in Sub-Saharan Africa have found that such qualitative traits have a greater role in determining a grower’s preference than economic traits such as yield [7], [45]. These private preferences or perceptions of risk are intangible and though they are somewhat encapsulated with our “cost of control” parameter will vary from grower to grower.

For simplicity, in both our stochastic and deterministic models we included an average loss of yield. In reality, yield will depend on how far into the growing season the field was infected and the rate of within-field disease spread. The inclusion of time-dependent yield loss will favour clean seed use, as these fields will on average be infected later in the growing season than conventional fields. Indeed, this could shift the balance of payoffs such that the payoff for an infected field planted with clean seed is no longer the lowest possible payoff. If this shift were observed, an “all control” equilibrium may now be stable in the outcome-based comparisons that we have described.

Though we have not investigated it here, CSS can “bundle” desirable traits by providing CBSD resistant or tolerant planting material, changing the nature of the externalities generated by clean seed use. There are few such varieties available for cassava. Provision of resistant material, though of obvious benefit to the recipient, will also increase the benefit experienced by free riders by reducing their probability of infection. Cassava varieties that are tolerant to CBSD infection will benefit the focal grower, but may increase the disease pressure experienced by other growers and thus incentivise control. These are potentially important distinctions, which we intend to return to in future work.

Growers’ inability to properly perceive epidemiological parameters had varying effects on CSS participation. Underestimating the rate of horizontal transmission, β, increased participation as

growers believed that infection via this route was unlikely (Figure 6(A)). However, there was still a high probability of vertical transmission so control was worthwhile. As estimates of β increased, control was less favourable and participation decreased. A similar trend was observed in the spatial model when estimating α, the whitefly's dispersal scale parameter (Figure 9). Misestimations of the proportions of infected individuals had a non-monotonic pattern, with the proportion of controllers initially increasing with the perceived number of infected fields ($q_I I$) before falling as more controllers become infected and thus receive the lowest payoff (Figure 6(B)).

It is therefore important to understand how growers perceive their probability of infection, as this will influence their participation in control schemes and subsequent spread of disease. Overestimating the threat can discourage participation due to the perceived inevitability of infection regardless of any actions taken to prevent it. The effect of underestimation depends on the route of disease transmission in question, but control only remains attractive if it is seen as providing sufficient benefits. Regardless, lowering the cost of control increased participation. Again, here every grower has the same level of misperception, but in reality this will be closely linked to an individual grower's attitude towards risk, access to information and previous history of infection, which all will be highly variable between growers.

The inclusion of grower behaviour alters disease dynamics, as the option to participate in a control scheme lowered the probability of infection and increased the yield achieved by the growers. We have found that the means of inclusion of behaviour is also important; the quantities of comparison impact dynamics approaching equilibrium, but also determine the nature of the equilibria that can be achieved. Our investigation has been limited to one case study, but the decision modelling framework laid out in this paper is sufficiently flexible to be incorporated into a range of other disease systems. It would also allow grower behaviour to feed into other types of study, for example those in which control has a spatial element and/or is done reactively in response to detected infection ([46], [47], [48], [49]), or where estimates of parameters become more accurate over time and as disease spreads [50].

5 Acknowledgments

REMW acknowledges the Biotechnology and Biological Sciences Research Council of the United Kingdom (BBSRC; https://bbsrc.ukri.org/) for support via a University of Cambridge DTP PhD studentship (Project Reference 2119272).

References

[1] Bauch CT, Earn DJD. Vaccination and the theory of games. Proceedings of the National Academy of Sciences. 2004;101(36):13391-4.

[2] Chang SL, Piraveenan M, Pattison P, Prokopenko M. Game theoretic modelling of infectious disease dynamics and intervention methods: a review. Journal of Biological Dynamics. 2020;14(1):57-89. PMID: 31996099. Available from: https://doi.org/10.1080/17513758.2020.1720322.

[3] Delabouglise A, Boni MF. Game theory of vaccination and depopulation for managing livestock diseases and zoonoses on small-scale farms. Epidemics. 2020;30:100370.

[4] Mohr S, Beard R, Nisbet AJ, Burgess STG, Reeve R, Denwood M, et al. Uptake of Diagnostic Tests by Livestock Farmers: A Stochastic Game Theory Approach. Frontiers in Veterinary Science. 2020;7:36. Available from: https://www.frontiersin.org/article/10.3389/fvets.2020.00036.

[5] Milne AE, Bell JR, Hutchison WD, van den Bosch F, Mitchell PD, Crowder D, et al. The Effect of Farmers' Decisions on Pest Control with Bt Crops: A Billion Dollar Game of Strategy. PLOS Computational Biology. 2016 12;11(12):1-18.

[6] McQuaid CF, Gilligan CA, van den Bosch F. Considering behaviour to ensure the success of a disease control strategy. Royal Society Open Science. 2017a;4(12):170721. Available from: https://royalsocietypublishing.org/doi/abs/10.1098/rsos.170721.

[7] Szyniszewska AM, Chikoti PC, Tembo M, Mulenga R, Gilligan CA, van den Bosch F, et al. Cassava planting material movement and grower behaviour in Zambia: implications for disease management. bioRxiv. 2019. Available from: https://www.biorxiv.org/content/early/2019/03/06/528851.

[8] Milne AE, Gottwald T, Parnell SR, Alonso Chavez V, van den Bosch F. What makes or breaks a campaign to stop an invading plant pathogen? PLOS Computational Biology. 2020 02;16(2):1-20. Available from: https://doi.org/10.1371/journal.pcbi.1007570.

[9] Bate AM, Jones G, Kleczkowski A, Touza J. Modelling the effectiveness of collaborative schemes for disease and pest outbreak prevention. Ecological Modelling. 2021;442:109411. Available from: https://www.sciencedirect.com/science/article/pii/S0304380020304701.

[10] Siegal G, Siegal N, Bonnie RJ. An Account of Collective Actions in Public Health. American Journal of Public Health. 2009;99(9):1583-7. PMID: 19608946. Available from: https://doi.org/10.2105/AJPH.2008.152629.

[11] Geoffard PY, Philipson T. Disease Eradication: Private versus Public Vaccination. The American Economic Review. 1997;87(1):222-30. Available from: http://www.jstor.org/stable/2950864.

[12] Morris P. Introduction to Game Theory. Universitext. Springer; 2012.

[13] Bauch CT. Imitation dynamics predict vaccinating behaviour. Proceedings of the Royal Society B: Biological Sciences. 2005;272(1573):1669-75.

[14] Monger WA, Seal S, Isaac AM, Foster GD. Molecular characterization of the Cassava brown streak virus coat protein. Plant Pathology. 2001;50(4):527-34.

[15] Patil BL, Ogwok E, Wagaba H, Mohammed IU, Yadav JS, Bagewadi B, et al. RNAi-mediated resistance to diverse isolates belonging to two virus species involved in Cassava brown streak disease. Molecular Plant Pathology. 2011;12(1):31-41.

[16] Hillocks RJ, Raya MD, Mtunda K, Kiozia H. Effects of Brown Streak Virus Disease on Yield and Quality of Cassava in Tanzania. Journal of Phytopathology. 2001;149(7-8):389-94. Available from: https://onlinelibrary.wiley.com/doi/abs/10.1111/j.1439-0434.2001.tb03868.x.

[17] Legg J, Somado E, Barker I, Beach L, Ceballos H, Cuellar W, et al. A global alliance declaring war on cassava viruses in Africa. Food Security. 2014;6:231–248. Available from: https://doi.org/10.1007/s12571-014-0340-x.

[18] Maruthi MN, Hillocks RJ, Mtunda K, Raya MD, Muhanna M, Kiozia H, et al. Transmission of Cassava brown streak virus by Bemisia tabaci (Gennadius). Journal of Phytopathology. 2005;153(5):307-12. Available from: https://onlinelibrary.wiley.com/doi/abs/10.1111/j.1439-0434.2005.00974.x.

[19] Chipeta MM, Shanahan P, Melis R, Sibiya J, Benesi IRM. Farmers' knowledge of cassava brown streak disease and its management in Malawi. International Journal of Pest Management. 2016;62(3):175-84. Available from: https://doi.org/10.1080/09670874.2016.1167268.

[20] McQuaid CF, van den Bosch F, Szyniszewska A, Alicai T, Pariyo A, Chikoti PC, et al. Spatial dynamics and control of a crop pathogen with mixed-mode transmission. PLOS Computational Biology. 2017b 07;13(7):1-18. Available from: https://doi.org/10.1371/journal.pcbi.1005654.

[21] Legg J, Ndalahwa M, Yabeja J, Ndyetabula I, Bouwmeester H, Shirima R, et al. Community phytosanitation to manage cassava brown streak disease. Virus Research. 2017;241:236-53. Plant Virus Epidemiology. Available from: https://www.sciencedirect.com/science/article/pii/S0168170217301533.

[22] Madden LV, Hughes G, van den Bosch F. The study of plant disease epidemics. St Paul, MN: American Phytopathological Society (APS Press); 2007.

[23] Szyniszewska AM. CassavaMap, a fine-resolution disaggregation of cassava production and harvested area in Africa in 2014. Scientific data. 2020;7:159.

[24] Jeger MJ, Holt J, van den Bosch F, Madden LV. Epidemiology of insect-transmitted plant viruses: modelling disease dynamics and control interventions. Physiological Entomology. 2004;29:291-304.

[25] Ephraim N, Yona B, Evans A, Sharon A, Titus A. Effect of cassava brown streak disease (CBSD) on cassava (Manihot esculenta Crantz) root storage components, starch quantities and starch quality properties. International Journal of Plant Physiology and Biochemistry. 2015.

[26] Byrne DN. Migration and dispersal by the sweet potato whitefly, Bemisia tabaci. Agricultural and Forest Meteorology. 1999;97(4):309-16. Available from: https://www.sciencedirect.com/science/article/pii/S016819239900074X.

[27] Fu F, Rosenbloom DI, Wang L, Nowak MA. Imitation dynamics of vaccination behaviour on social networks. Proceedings of the Royal Society B: Biological Sciences. 2011;278(1702):42-9. Available from: https://royalsocietypublishing.org/doi/abs/10.1098/rspb.2010.1107.

[28] Cunniffe NJ, Gilligan CA. Use of Mathematical Models to Predict Epidemics and to Optimize Disease Detection and Management. In: Ristaino JB, Records A, editors. Emerging Plant Diseases and Global Food Security. APS Press; 2020. .

[29] Fabre F, Coville J, Cunniffe NJ. Optimising reactive disease management using spatially explicit models at the landscape scale. In: Scott P, Strange R, Korsten L, Gullino ML, editors. Plant Diseases and Food Security in the 21st Century:. Springer; 2021. .

[30] Keeling MJ, Rohani P. Modeling Infectious Diseases in Humans and Animals. Princeton University Press; 2008. Available from: http://www.jstor.org/stable/j.ctvcm4gk0.

[31] van den Bosch F, Jeger MJ, Gilligan CA. Disease control and its selection for damaging plant virus strains in vegetatively propagated staple food crops; a theoretical assessment. Proceedings of the Royal Society B: Biological Sciences. 2007;274(1606):11-8. Available from: https://royalsocietypublishing.org/doi/abs/10.1098/rspb.2006.3715.

[32] Gibson RW, Otim-Nape GW. Factors determining recovery and reversion in mosaic-affected African cassava mosaic virus resistant cassava. Annals of Applied Biology. 1997;131(2):259-71. Available from: https://onlinelibrary.wiley.com/doi/abs/10.1111/j.1744-7348.1997.tb05155.x.

[33] Mohammed IU, Ghosh S, Maruthi MN. Host and virus effects on reversion in cassava affected by cassava brown streak disease. Plant Pathology. 2016;65(4):593-600. Available from: https://bsppjournals.onlinelibrary.wiley.com/doi/abs/10.1111/ppa.12458.

[34] Byrne DN, Rathman RJ, Orum TV, Palumbo JC. Localized migration and dispersal by the sweet potato whitefly, Bemisia tabaci. Oecologia. 1996;105:320-8.

[35] Hamelin FM, Bowen B, Bernhard P, Bokil VA. Optimal Control of Plant Disease Epidemics with Clean Seed Usage. Bulletin of mathematical biology. 2021;83 5:46.

[36] Bauch CT, Galvani AP, Earn DJD. Group interest versus self-interest in smallpox vaccination policy. Proceedings of the National Academy of Sciences. 2003;100(18):10564-7. Available from: https://www.pnas.org/content/100/18/10564.

[37] Wu B, Fu F, Wang L. Imperfect Vaccine Aggravates the Long-Standing Dilemma of Voluntary Vaccination. PLOS ONE. 2011 06;6(6):1-7. Available from: https://doi.org/10.1371/journal.pone.0020577.

[38] Shaw MW. Simulation of population expansion and spatial pattern when individual dispersal distributions do not decline exponentially with distance. Proceedings of the Royal Soci-

ety of London Series B: Biological Sciences. 1995;259(1356):243-8. Available from: https://royalsocietypublishing.org/doi/abs/10.1098/rspb.1995.0036.

[39] Ntawuruhunga P, Legg J, Okidi J, Okao-Okuja G, Tadu G, Remington T. Southern Sudan, Equatoria Region, Cassava Baseline Survey Technical Report. IITA. 2007 08.

[40] Chikoti P, Melis R, Shanahan P. Farmer's Perception of Cassava Mosaic Disease, Preferences and Constraints in Lupaula Province of Zambia. American Journal of Plant Sciences. 2016:1129-38.

[41] Djaha KE, Abo K, Kone T, Kone D, Kone M. Analysis of the population structure of cassava growers, production systems, and plots' sanitary state in Côte d'Ivoire. Journal of Animal & Plant Sciences. 2018:5833-43.

[42] Houngue JA, Pita JS, Cacaï GHT, Zandjanakou-Tachin M, Abidjo EAE, Ahanhanzo C. Survey of farmers' knowledge of cassava mosaic disease and their preferences for cassava cultivars in three agro-ecological zones in Benin. Journal of Ethnobiology Ethnomedicine. 2018.

[43] Teeken B, Olaosebikan O, Haleegoah JAS, Oladejo E, Madu TU, Bello A, et al. Cassava Trait Preferences of Men and Women Farmers in Nigeria: Implications for Breeding. Economic Botany. 2018;72:263 277.

[44] Zhang R, Brennan TJ, Lo AW. The origin of risk aversion. Proceedings of the National Academy of Sciences. 2014. Available from: https://www.pnas.org/content/early/2014/11/26/1406755111.

[45] Houngue JA, Pita JS, Cacaï GHT, Zandjanakou-Tachin M, Abidjo EAE, Ahanhanzo C. Survey of farmers' knowledge of cassava mosaic disease and their preferences for cassava cultivars in three agro-ecological zones in Benin. Ethnobiology Ethnomedicine. 2019;14(29). Available from: https://doi.org/10.1186/s13002-018-0228-5.

[46] Cunniffe NJ, Stutt ROJH, DeSimone RE, Gottwald TR, Gilligan CA. Optimising and Communicating Options for the Control of Invasive Plant Disease When There Is Epidemiological Uncertainty. PLOS Computational Biology. 2015 04;11(4):1-24. Available from: https://doi.org/10.1371/journal.pcbi.1004211.

[47] Cunniffe NJ, Cobb RC, Meentemeyer RK, Rizzo DM, Gilligan CA. Modeling when, where, and how to manage a forest epidemic, motivated by sudden oak death in California. Proceedings of the National Academy of Sciences. 2016;113(20):5640-5. Available from: https://www.pnas.org/content/113/20/5640.

[48] Hyatt-Twynam SR, Parnell S, Stutt ROJH, Gottwald TR, Gilligan CA, Cunniffe NJ. Risk-based management of invading plant disease. New Phytologist. 2017;214(3):1317-29. Available from: https://nph.onlinelibrary.wiley.com/doi/abs/10.1111/nph.14488.

[49] Laranjeira FF, Silva SXB, Murray-Watson RE, Soares ACF, Santos-Filho HP, Cunniffe NJ. Spatiotemporal dynamics and modelling support the case for area-wide management of citrus greasy spot in a Brazilian smallholder farming region. Plant Pathology. 2020;69(3):467-83. Available from: https://bsppjournals.onlinelibrary.wiley.com/doi/abs/10.1111/ppa.13146.

[50] Thompson RN, Gilligan CA, Cunniffe NJ. Control fast or control smart: When should invading pathogens be controlled? PLOS Computational Biology. 2018 02;14(2):1-21. Available from: https://doi.org/10.1371/journal.pcbi.1006014.

[51] Diekmann O, Heesterbeek JAP, Roberts MG. The construction of next-generation matrices for compartmental epidemic models. Journal of The Royal Society Interface. 2010;7(47):873-85. Available from: https://royalsocietypublishing.org/doi/abs/10.1098/rsif.2009.0386.

[52] van den Driessche P. Reproduction numbers of infectious disease models. Infectious Disease Modelling. 2017;2(3):288-303. Available from: https://www.sciencedirect.com/science/article/pii/S2468042717300209.

Click here to access/download
Supporting Information
Grower_behaviour_paper-50-64.pdf
