# Supplementary material for: How growers make decisions impacts plant disease control
Source: PLoS Comput Biol. 2022 Aug 22;18(8):e1010309. doi: 10.1371/journal.pcbi.1010309 (PMC9394827; doi:10.1371/journal.pcbi.1010309)
Supplement: Attachment — Submitted filename: Review of PCOMPBIOL.docx [file pcbi.1010309.s005.docx]

Review of PCOMPBIOL-D-21-02295, “It ain't what you do, it's what drives why you do it: how the basis of growers' decision making affects the effectiveness of disease control”

**General appraisal**

This is a novel, sound and scientifically valuable paper which adds to our understanding of how the dynamics of growers’ behaviour impacts the management of plant diseases with a particular focus on CBSD. The model explanation and development are very clear and enjoyable to read, but the organisation of the paper is not very good. There are 4 different “models” that the author explores, with focus on one of them. However, it is often difficult to know which one the author is talking about, especially when discussing the results.

**General comments**

In this manuscript the authors develop a model to understand how the growers’ decision to use Cassava Clean Seed Systems (CSS) for disease control, affects the dynamics of the spread of Cassava Brown Streak Disease (CBSD). To model the behaviour of farmers, the authors use game theory to model 4 different types of scenarios that a grower can follow to maximise their profit when considering the adoption of CSS. For the dynamics of CBSD spread they use a process based deterministic formulation that is then extended to a spatial and stochastic model.

The paper is enjoyable to read in some parts and the model is very well explained and easy to follow. However, the organisation of the different sections and presentation of the results is not well organised. This really needs a revision to make the document flow. I made some comments in sections where it was clear to me it needed to change, but please carefully review the document and make sure it flows.

The figures also need careful revision. Often the caption is lacking all the elements needed to fully understand the figure and the reader needs to keep on navigating through the text to understand it. Make sure that all the parameters used in the figures are explained and that the legends are large enough to read.

Finally, I think the authors need to re-focus the questions they explore (lines 92-98) so the results follow from that and the paper impact is clearer. There are some very interesting results that get lost among the text which could be highlighted from the beginning in the abstract and also in the discussion. As such, the discussion needs to re-focus and really show the most interesting results found throughout the text.

Nonetheless, I think this is a very interesting paper with novel approaches and non-intuitive results that the authors nicely linked to their counterparts in human epidemiology. Reorganising the flow, carefully curating the figures and making sure of defining all parameters and variables would make this a much better manuscript.

**Specific comments**

1. It would be great if the authors could give examples of systems where each grower strategy may happen. For example, in the case grower vs. population, I can think of an organic farmer that has a larger profit due to the “premium” that the organic label confers them. In a region where spraying for disease control is the norm, they may choose to reject spraying to keep their higher profit in comparison to the profit of the spraying population.
2. Detailed comments are provided in an annotated PDF version of the submitted manuscript.
