# Supplementary material for: How growers make decisions impacts plant disease control
Source: PLoS Comput Biol. 2022 Aug 22;18(8):e1010309. doi: 10.1371/journal.pcbi.1010309 (PMC9394827; doi:10.1371/journal.pcbi.1010309)
Supplement: Attachment — Submitted filename: PLOS_Computational_Biology_resubmissioncover_letter.docx [file pcbi.1010309.s007.docx]

| Department of Plant Sciences  University of Cambridge  Downing Street  Cambridge, CB2 3EA |   **Department of Plant Sciences** |
| --- | --- |
| 27^th^ May 2022 |  |

Dear Editor,

**How growers make decisions impacts plant disease control**

*Rachel E Murray-Watson, Frédéric M Hamelin and Nik J Cunniffe*

Thank you for handling our manuscript and for facilitating such useful reviews. Please find below our response to the reviewers’ comments.

We have acted on all requested changes but one. We have chosen not to act on Reviewer 2’s suggestion of a new terminology, as we believe that given the lack of standard terms relating to these types of model in the literature, choosing our own terminology and clearly defining them in the manuscript is sufficient. However, we are happy to adopt the reviewer’s suggestion, if it is also preferred by the Editor.

As requested, we have submitted a “*Track changes*” version of the m/s showing alterations from the last version.

Rachel Murray-Watson

**Reviewer 1:**

Reviewer #1: I am grateful to the authors for their thoughtful and comprehensive response to the suggestions made on the previous draft of the paper. I have no further comments.

Response: We thank the reviewer for their appraisal of the manuscript, and for their initial comments which improved the overall work.

Action: NA

**Reviewer 2:**

First, I have a quick title suggestion: “How growers make decisions impacts plant disease control”.

Response: We thank the reviewer for this suggestion and agree that it is a better title for our paper.

Action: We have changed the title from “How grower decision-making is modelled impacts the effect of plant disease control” to “How growers make decisions impacts plant disease control”.

Second, I still think using “strategy vs” and “grower vs” are poor choices to describe the two classes models. Imagine if you were someone for whom English was a second language, you read this article and liked it and then wanted to talk to native speakers in the field about it. If you then started talking to them about a “strategy vs” model or a “grower vs” model, thinking this was a standard way of talking about such models, no one would know what you were talking about. I suggested finding a better terminology for the purpose of helping people understand and communicate about your paper. As it stands, the current terminology is an impediment, even among native speakers.

Response: Whilst we thank the reviewer for highlighting this, we do not agree that the current terminology is an impediment to discussing the paper. As it stands, and as you highlight, there is no standard terminology for these models which facilitates communication, whatever the languages of the participants in any conversation. We have defined (both in words and mathematically) what we mean by our choice of terminology to ensure that, if an individual wished to discuss the models, they would be able to define them.

Action: None taken.

Micro comments
Line 29: drop “do”

Response: We thank the reviewer for making this suggestion, which adds clarity to our sentence.

Action: Line 29: “Yet if many growers do participate in a control scheme” -> “Yet if many growers participate in a control scheme”

Line 111: use “a different”, not “an”

Response: We thank the reviewer spotting this typo.

Action: Line 111: Changed “an different” to “a different”

Line 351: something is missing, maybe “controlling it does not …”?

Response: We thank the reviewer noting this, and agree that the should be changed for clarity.

Action: Line 35: “even all growers controlling it not guarantee” -> “even if all growers control it does not guarantee”
