# Supplementary material for: How growers make decisions impacts plant disease control
Source: PLoS Comput Biol. 2022 Aug 22;18(8):e1010309. doi: 10.1371/journal.pcbi.1010309 (PMC9394827; doi:10.1371/journal.pcbi.1010309)
Supplement: Attachment — Submitted filename: PLOS_Comp_Bio_cover_letter_second_review.docx [file pcbi.1010309.s008.docx]

| Department of Plant Sciences  University of Cambridge  Downing Street  Cambridge, CB2 3EA |   **Department of Plant Sciences** |
| --- | --- |
| 20^th^ April 2022 |  |

Dear Editor,

**How grower decision-making is modelled impacts the effect of plant disease control.**

*Rachel E Murray-Watson, Frédéric M Hamelin and Nik J Cunniffe*

Thank you for handling our manuscript and for facilitating such useful reviews. Please find below our response to the reviewers’ comments.

We have acted on essentially all proposed changes, most notably by making significant structural changes to reorganise the manuscript and make the major results easier to digest by more closely focusing on a smaller number of questions (with various other, less important, results now de-emphasised and put in the Supplementary Materials). There has been a corresponding reduction in the number of figures in the main text from nine to six, reflecting how we have more tightly focused on a smaller number of key messages. We have also added an explicit recognition of their significant input in the “*Acknowledgements*” section.

The only exception is the suggestion by Reviewer 2 to change to an *Introduction – Results – Discussion – Methods* format. A key focus of our paper is the difference between what we term the “strategy vs.” and “grower vs.” models (which follow what Reviewer 2 calls “rational” and “strategic-adaptive” expectations respectively). Understanding the difference between these ways in which growers make decisions, as currently outlined in the *Methods*, is therefore a pre-requisite for interpreting the *Results*. Although we did carefully consider altering the format to put the *Results* first, we were unable to identify a workable way of doing this while keeping the m/s understandable. We have therefore retained the current *Introduction – Methods - Results – Discussion* format.

We note that some of us have used the *Introduction – Methods - Results – Discussion* format a number of times for publications in this journal, e.g. Cunniffe *et al.* (2021) *PLOS Computational Biology* 17:e1009759; Cunniffe *et al.* (2015) *PLOS Computational Biology* 11:e1004211; Cunniffe *et al.* (2014) *PLOS Computational Biology* 10: e1003753, and so hope the Editors will permit us to use it here for this paper, too.

As requested, we have submitted a “*Track changes*” version of the m/s showing alterations from the last version. However, given the number of changes, this is rather unwieldy, and so line numbers in the below are given relative to the “*Clean*” version of the updated m/s.

Rachel Murray-Watson

**General Comments**

We thank the Editors and all three Reviewers for their helpful comments, which have undoubtedly enhanced the quality of this piece of work.

**Editors’ Comments**

**In addition to the recommendations formulated by all three reviewers regarding the reorganization of the main content of this manuscript, the title and first sentence should emphasize stronger that this work addresses only plant diseases.**

**Response:** As stated above, we have made significant structural changes to the m/s to make our work easier to follow. We have also adopted the Editors’ suggestion to emphasise that our work relates to plant disease epidemics.

**Action:** We have altered the title and the first sentence of the Introduction (Line 26) as requested (and various other changes throughout, as described below).

**Reviewers’ Comments**

*Reviewer 1*

**Congratulations to the authors on writing a coherent and interesting paper on an area that is in need of new ideas and robust methodology. I think this is an important contribution to the interdisciplinary literature. In a paper of this technical complexity it is not possible to check in detail all of the mathematics and computational results. As far as possible in the time available I tracked the argument developed through the equations provided. This somewhat superficial evaluation seemed to accord with the results offered. Work of this type tends to be fully evaluated only after publication. I can see no technical reasons to deny publication, based on my reading.**

**Response:** We thank the reviewer for this very positive summary of our work.

**Action:** None requested.

**Lines 32-35. It would be good to have a citation that demonstrated that these intuitions are, in fact, correct; both those relating to disease prevalence and cost, and those relating to the actions of other growers. Recent work on HLB by Garcia Figuera et al. might be useful in this context, but since this is work from my group there is a conflict of interest in me promoting it. However there may be other works in the reference lists of these papers that would be useful. In any case, I would be inclined (if I was writing the current paper) to say that these are underlying assumptions of the model. They are not unreasonable assumptions to make of a rational agent.**
[**https://doi.org/10.1007/s12571-020-01133**](https://doi.org/10.1007/s12571-020-01133)
[**https://doi.org/10.1094/PHYTO-12-20-0544-R**](https://doi.org/10.1094/PHYTO-12-20-0544-R)

**Response:** We thank the reviewer for this useful suggestion and agree that adding in references to support our intuition is useful.

**Action:** We have added in two references at this point: Sherman and Gent, 2004 and Garcia-Figuera *et al.* 2021 (Line 34). The first talks about a variety of personal and economic factors (suggested by Reviewer 2), the second about HLB management and information networks/perceived risk (as suggested by the Reviewer 1). We have also clarified that these statements are assumptions by stating: “We assume that decisions regarding disease control are intrinsically linked…”.

**Line 43-44. “Yet if too many growers engage in free-riding behaviour, there will be a resurgence of infections, making disease control more likely” Perhaps, but empirical studies (see above, and also work by Ariel Singerman in Florida) find that growers report one of the main reasons they do not participate in collective action is that they think others will free ride. Once disease starts to spread they point to the uselessness of further collaboration and the resulting lack of coordination leads to a diversity of responses; it’s not safe to assume that more disease results in more effort to reduce disease.**

**Response:** We thank the reviewer for this interesting observation, as well as the useful references. Given our later findings – that above a certain threshold of disease incidence, participation in control becomes unlikely – we do agree that the increased use of control with increased disease incidence is not axiomatic and are happy to be more precise here.

**Action:** We have amended the sentence as follows: “which may make disease control more likely” (Line 43), and have added the following: “Indeed, perceived risk of other growers benefiting from an individual's control policies are a prominent reason why growers do not participate in control schemes ([18],[23])”. (Lines 47-48).

**Lines 53-62. I’m very much in favour of pursuing a game-theoretic approach in studying this kind of problem, but this paragraph highlights one of the issues. Most growers (at least in the systems with which I’m familiar) have only a vague idea about each others’ or the industry average costs. The authors acknowledge this unrealistic assumption on line 61. Whether it’s an important issue will depend on what role games with this type of information structure play in the subsequent analysis.**

**Response:** We acknowledge that assuming “perfect information” is unrealistic, though is common practice in game-theoretic models. However, we do not believe that this detracts from the main points of our paper, and indeed our different formulations of the decision model allow for differing degrees of information availability. We also note that we explicitly test the effect of systematic uncertainty in growers’ estimation of the current state of the system upon our results (our Q3), and so show our results are relatively robust in at least some cases when information is limited.

**Action:** Line 57 – 61: We have now added more text justifying our use of game theory and further acknowledging that the model we present is a limited, though useful, tool in understanding the broader issue.

**Lines 63-69. This paragraph is confusing. It is not explained in advance what is meant by “the alternative strategy”. An introductory sentence before Line 53 explaining the overall approach would be useful. I would reword the last sentence (lines 68-69) to make it clearer that you mean any games of the type that compare the growers’ strategy with another strategy; perhaps just include a placeholder after the vs. inside the quotation marks “grower vs. X”?**

**Response:** We thank the reviewer for highlighting this lack of clarity and for suggestions of how to ameliorate it. We agree that by discussing our terminology and approach more at this point the reader will gain a better understanding of the models and how they differ.

**Action:** Lines75-76: We have added a definition of “alternative strategy” as: the strategy that the grower does not currently use; for a controller, this would be non-control and vice versa. We have also added the following overview of the approach we take: “In our work, we assume growers have access to different combinations of three pieces of information: their own yield from the previous year, the expected profits of each strategy (control or non-control) and the overall expected profit of the population. They then compare these different quantities to assess profitability and thus whether they should change strategy. We have also modified the final sentence in this paragraph for clarity: To our knowledge, the ``grower vs. population" and ``grower vs. alternative" models have never been compared.

**Table 2. (Line 170) In the table heading, include a remark about whether the tick or cross mean the information is needed. It’s not difficult to work out, but give the reader a break.**

**Response:** We thank the reviewer for this suggestion, and agree that it will assist the reader.

**Action:** Table 2 (Line 244): We have added this clarification “The   refers to when information is required, and "X" when it is not necessary for decision-making.”

**Line 207. insert “s” on the end of depend to match tense.**

**Response:** We thank the reviewer for spotting this typo.

**Action:** Line 274: We have added “s” to end of “depend”.

**Line 301. Italics needed for Bemesia tabasci Is this the first full mention? If not then shorten to**

**B. tabaci**

**Response:** We thank the reviewer for spotting this inconsistency, as it was not the first full mention of *Bemisia tabasci.*

**Action:** Appendix 2 Line 907: We have ensured that *Bemisia tabasci* is italicised in all places in the text and have shortened it to *B. tabasci* where appropriate.

**Line 337. add a reminder of what eta is to help with interpretation of the description of the dependence**

**Response:** We thank the reviewer for highlighting this and agree that adding clarification ill assist the reader.

**Action:** We have added clarification of the meaning of η as “the responsiveness of growers”. We note that these results have now been moved to Appendix 4 Line 1105 – 1120.

**Lines 347-351. I’m not completely happy with the way you word the game’s mechanism for switching here. In the model, the mathematics ensures that the equilibrium solution contains a mix of strategies, but you stray into couching things in terms of what an actual human agent might do – “considers changing strategy” – and you’re not representing a process at that level of detail. I sympathize. It’s hard to construct this kind of model without attaching human attributes to the mechanisms, but perhaps this is one step too far that direction?**

**Response:** We thank the reviewer for this interesting observation. Though we do model human decision making, the reviewer is correct in saying that there is a distinction between what happens mathematically and what we desire our model to represent, and that it is useful to be more precise here.

**Action:** Line 361 – 362: We have changed the wording to be: “any non-controlling grower with an infected field ($I_N$) has a non-zero probability of changing strategy”.

**Line 358. I don’t think you’re measuring the pathogen’s R in this work. Your results all relate to fields as the unit of measurement so your estimates of R_{0}R0
relate to diseased fields, no?**

**Response:** We agree with the reviewer that our language here was imprecise, and that indeed we were measuring the R_0 of the field-scale system.

**Action:** Line 307: We have rephrased our definition of R_0 in terms of being at the “field-scale”

**Line 438-440. This is an important (if intuitively obvious) result. In driving/leading a population towards adoption of control this would be a central extension message. It would also be an important message to take to policy-makers about how funding incentives for control would be worthwhile. It’s important that the work reached this conclusion, because (earlier comments about avoiding over-interpreting the models in human terms notwithstanding) the models make relatively mild and rational assumptions about choice.**

**Response:** We thank the reviewer for this positive comment, as well as for contextualising our result in terms of how it would be useful in a real-world context.

**Action:** Line 400-410: We have added text to the end of this section summarising the important parameters for encouraging participation in control, emphasising this conclusion.

**Figure 9. Vertical axis represents a number but is labeled as a proportion.**

**Response:** We thank the reviewer for alerting us to this mistake

**Action:** We have updated the figure so that the y-axis is labelled as “Proportion of fields”. We note that this is now Figure 3 of Appendix 3, rather than Figure 9 in the main text.

**Lines 510-519. The comparison of outcomes predicted by Game Theory approaches with reality is interesting. It perhaps says as much about the limitations of the approach as it does about human nature? If the comment that the actual level of vaccination achieved will be less than that required for herd immunity is important to the rest of the paper it needs to be spelled out a bit more; it’s not intuitive, at least to me, why this should be a general result. The argument advanced in lines 515-519 is also not convincing. At least in the USA, one of the most commonly offered arguments against getting vaccinated against COVID-19 is that the vaccine is imperfect and allows transmission and infection; at least for these agents, both the rationale and the outcome run counter to that apparently predicted by standard Game Theory approaches?**

**Response:** We thank the reviewer for their comment, and apologise for the lack of clarity. The text that is referred to in this comment only concerns the limiting case of perfect control, which is not what our control mechanism (the CSS) provides. We agree that the way this was phrased would have confusing for the reader of our original submission.

**Action:** Line 466 – 481: We have expanded this paragraph to go into more detail of the previous studies and – crucially – how the assumptions about the effectiveness of control differ from what we consider here. We have also added additional text describing the nature of the free-rider problem and how, in previous theoretical studies, it has proved to be a limiting factor to disease control.

**Line 530. The words “alone” and “vector” need to be swapped in order.**

**Response:** We thank the reviewer for spotting this typo.

**Action:** Line 481: We have corrected the sentence such that it reads “the vector alone”.

**Lines 532-534. This is a nice result. A primia facie argument that CSS would not be worth buying if disease can spread horizontally and spoil one’s investment is enticing, but the results indicate that the conditions for adoption of CSS are more subtle than that; if disease can’t spread it’s not needed, and if it spreads too readily one can’t protect one’s investment. It seems that at least in some of the modeled scenarios the threshold disease spread at which CSS becomes worthwhile should be derivable? Would it be where the expected loss caused by the (uncertain) horizontal spread equals the cost of CSS?**

**Response:** We thank the reviewer for this positive comment. The reviewer is correct that for the “strategy vs.” models, the boundaries between each possible equilibrium (control-only, no-control, disease-free and both strategies) are derivable and presented in Appendix 1. These conditions will then predict what the threshold values for changing strategy. Whether it occurred at the threshold described by the reviewer would depend on the probability of infection (both vertical and horizontal), as the loss due to disease is incurred by both controllers and non-controllers. Therefore, if there is a high probability of being infected via horizontal transmission, the loss will be sustained by growers of both strategies, whilst the controllers will have to pay the additional cost of control. However, if infection only occurs via vertical transmission (and thus does not affect controllers), the threshold described by the reviewer would be correct.

**Action:** Line 484 – 489: We have added text to this part of the text directing the reader to the appendix, where the stability conditions are laid out, as well as a brief summary of what these conditions depend upon.

**Lines 535-539. I agree with the authors here. The “grower vs alternative” evaluation most closely approximates the approach taken by growers with whom I have worked and observed.**

**Response:** We thank the reviewer this comment supporting our conclusions. This is a useful reassurance for us, in the light of our decision to continue only with the ``grower vs. alternative” model for the bulk of our investigations.

**Action:** None requested.

*Reviewer 2*

**Reviewer #2: The manuscript is overall well written and summarizes a large amount of high-quality work. My comments at this time are fairly general and higher level and meant to improve the overall manuscript.

First, the authors should structure the manuscript as described in the Submission Guidelines. Specifically, the Materials and Methods sections should be moved to the end of the main text. this organization change from the “classic” organization is useful for mathematical model papers of this sort, as it emphasizes the results and discussion so that readers understand the paper’s contributions and can then bury themselves in the details of the modeling if desired. Besides this organizational issue, in my opinion, the paper is trying to do too much. The current version is quite long and yet still too terse in many key parts to understand the findings, their nuances, and their wider connections to the literature.**

**Response**: We acknowledge that the having the Materials and Methods after the Results/Discussion is the normal format for this journal. However, we strongly believe that because a large portion of our results hinge on understanding the difference between our model formulations (i.e. understanding the difference between comparing the average of your strategy against the population, or your outcome from the previous season against the alternative strategy etc.) that presenting our results before laying out the derivation of these models (as would be the case with the Reviewer’s suggestion) would make the results uninterpretable. As described in our overall cover letter (above), we would therefore prefer not to adopt this suggestion from Reviewer #2.

However, we have undertaken several other major restructurings. Some aimed to simplify the presentation by attempting to present fewer results: the most obvious example is moving all details of the spatial-stochastic model to a new appendix, Appendix 3. However, we have also carefully thought about which results to present in the main manuscript, and focused our presentation around three questions (Lines 125 – 133), using the same structure to organise the different sections of our results.

We hope that, by doing this, we address the author’s concerns about the structure of the paper. By doing this, we have also been able to spend more time on explaining our key results and connecting them more to existing literature.

**Action**

- We have abbreviated the primary questions of investigation.
- We have restructured the Methods section so that the “Parametrisation” section is now Section 2.2.
- We have relegated all methodological details of – and results from – the spatial-stochastic version of our model from the main text to a new appendix (Appendix 2).
- We have abbreviated our results (moving much of the results relating to the “strategy vs.” models) to Appendices 1, 2 and 3 (including most of the mathematical detail).
- We have re-structured the Results section such that they follow directly from the questions posited in the introduction.

**The overall paper objective (lines 89-91) is good, but achieving it by answering the five questions (lines 91-98) are too many. The main question to examine is “How does including grower behaviour in plant epidemic models affect the outcome of control schemes, particularly when control strategies provide incomplete control?” (lines 89-93). *“With incomplete control, under what conditions, if any, can high levels of grower adoption of control strategies be attained and maintained?” “Discussion of results will focus on the implications for encouraging adoption of control strategies. Sensitivity analysis will examine how results respond to changes in key parameters and assumptions.”* I recommend dropping some of them or burying more of them in an appendix/supplement (or a second paper) and simply summarize the results of question 1 to 5 in the main text in order to focus on the main point. Right now your main point is buried under a huge pile of equations, variables, tables, figures and text, making it hard to find. Your goal should not be to summarizer all the work you have done (which is a lot), but to communicate your key finding by doing a better job of explaining it, its nuances, connecting it the existing literature and developing policy recommendations.**

**Response:** We thank the reviewer for this comment and acknowledge that there was concern across all reviewers regarding the number of issues we were trying to address in this single piece of work. We also acknowledge that including so much mathematical detail in the main text may be confusing and distracting for the reader.

**Action:** Lines 125 – 133: In light of this comment, we have reduced our focus to three questions: (1) How does the formulation of the comparison (i.e. ``strategy vs." or ``grower vs.") affect the model and its results? (2) How does grower participation in the CSS depend on epidemiologically and economically important parameters, and how can high levels of control be encouraged? (3) How does systemic uncertainty in epidemiological parameters affect participation in the CSS? We regard these to be the crux of our paper.

We have moved our investigation of spatial and stochastic effects to a new appendix (Appendix 2). We have removed much of the mathematical derivations from the main text (for example, moving the explanation of the stability conditions for the “strategy vs.” models or the explanation for how to achieve the “No control” equilibrium for the “grower vs. population” model) and included them in the appendices (Appendix 3).

**Second, I recommend tying the human behavior modeling more directly to the existing literature, including explaining and justifying assumptions. For example, equation 33 is simply posited with little explanation or tying to existing papers. See how Milne et al. (2015) (your citation 6) and Saikai et al. 2020 (DOI 10.1002/ps.6016) state and explain/justify it. Once you stop trying to put everything you have done into one paper and instead focus on the main point, you can bring readers along and help them understand what you are doing and connect to the existing academic conversation. There are many examples I could have noted.**

**Response:** We agree with the reviewer that adding a clarification of the switching terms at this point will assist the reader. We have reformatted this section such that the description of the different formulations of Δ comes before the general description of the switching terms.

**Action:** We have moved these lines (now lines 222-234) to the beginning of the section on behavioural models (now Section 2.6). Additionally, we have described the general formulations for each type of comparison at this point (which are then expanded upon in Section 2.6).

**Along these same lines, I recommend using what you call “strategy vs” or “grower vs” assumptions, not both, and then connecting them more clearly to terminology used in existing literature. The difference between the two is whether the farmer the previous year’s outcomes to form expected changes in profit or forms expectations using complete knowledge of the system. I think the difference it akin to what macro-economists call adaptive expectations versus rationale expectations.**

**Response:** We thank the reviewer for this interesting observation. After a literature appraisal, we agree that the “strategy vs.” models are akin to “rational” expectations in the literature. However, whilst we agree that our “grower vs.” models have aspects of “adaptive” expectations, we argue that they are more akin to the “strategtic-adaptive” expectations described in Fenichel and Wang 2013. <https://link.springer.com/chapter/10.1007/978-1-4614-5474-8_10>, and now use this terminology at an appropriate point in our paper.

However, we – respectfully – disagree that only one comparison should be used in this work. In our view a key component of our work is the explicit test of how different possible ways in which growers can make decisions affects the uptake of control, i.e. the first of our key questions as described above. Importantly, though, this element of model comparison is now restricted to a single section of the results, and the rest of the results all relate to the “grower vs. alternative” strategy comparison.

We acknowledge that our previous presentation would have made it somewhat difficult to “see the wood for the trees”, and so this element of our thinking might have been lost. As described above, we have undertaken extensive editing and restructuring of the paper – particularly the results section - we hope that the main messages of the paper have now become clearer.

**Action:** We have added a paragraph to the introduction of the paper (Lines 83 - 90) that links what we term “strategy vs.” and “grower vs.” models with the literature on rational and strategic-adaptive expectations.

**I spent some time looking in the results sections, but I was not able to easily figure out how the results differed for the two assumptions (adaptive vs rationale expectations). I suggest choosing one or the other, not both. Which one has the most empirical support or theoretical support? Which one have most researchers used? Only keep them if you get very different results and have a good reason that both may be accurate assumptions.**

**Response:** We apologise to the reviewer for the lack of clarity, and acknowledge that in addressing five – arguably slightly overlapping – questions our main messages were confused. As described above, we have chosen to keep the comparison between the different model types in the paper, though we have consolidated the comparison to one section (Section 3.1). We have then continued with only the “grower vs. alternative” model for the investigations into the parameter scans and the misestimation of parameters, as this has been more widely used in other literature and is more akin to the “responsive” grower that you describe.

**Action:** We have restructured our results section such that the results fall into three headings, each addressing a different one of the three questions listed at the very end of the Introduction, i.e. (1) Q1: Effect of model formulations on model equilibria (2) Q2: Effect of epidemiological and economic parameters on uptake of control and (3) Q3: Effect of systematic uncertainty. Remaining results have been moved to the Appendix. We have additionally offered an explanation of why we have chosen the “grower vs. alternative” model in particular (Lines 125 – 133). In summary, its previous use in plant epidemiological literature and resemblance to Kaup’s reflexive producer were the primary drivers for this choice.

**In addition, the send part of the comparison (alternative or postulation) I think should be tied to discussions of social networks or how farmers develop their information to use when making decisions. Wah you term “alternative’ is basic expected profit maximization, with no information about how neighbors have done, while “population” assumes the farmer has clear information on what the population average is. Kaup (Rural Sociology 73(1), 2008, pp. 62–81) is a good place to start, Sherman and Gent (2014)** [**http://dx.doi.org/10.1094/PDIS-03-14-0313-FE**](http://dx.doi.org/10.1094/PDIS-03-14-0313-FE) **overview the issues in Plant Disease, and also see Saikai et al. (2020) (DOI 10.1002/ps.6016).**

**Response:** We thank the reviewer for this comment and for the useful references. Whilst we agree with the reviewer that we should contextualise our results in terms of how growers access information, we disagree with the assessment here that the “alternative” strategy requires no information about how neighbours have done. To compare with the “alternative” strategy (i.e. the strategy that the grower is not currently using), they still must have information about what that strategy’s expected profit is (i.e. have perfect information on a subset of neighbours). Growers must still, therefore, have information concerning their neighbours’ profits. While we agree that social networks can be an important aspect of grower decision making, we also believe that evaluations of profit (i.e. making decisions on economic, rather than social factors) are of importance when considering growers’ behaviour. Additionally, the modelling paper provided here (Saikai et al 2020) is an agent-based model, for which it is easier to include social networks than in our non-spatial model. The focus of our work was creating a relatively simple and flexible non-spatial model whose “social network” approximates that of Milne’s “landscape network” (i.e. growers have information for all other growers in the landscape; Milne 2015).

**Action:** We have added a definition of “alternative strategy” (Lines 75-76) to clarify what we intended by the term. We have also added a paragraph about information networks amongst growers, and how different communities have access to different amounts of information (and have different degrees of trust in that information).

**In some sense, the choice between adaptive vs rationale expectations is also a similar information problem that Kaup examined. How does a farmer “know’ the real probability of infection and loss? Do they have to rely on their own experience or can they some how get access to the “true” probabilities? The same applies for the comparison group: how do they know the expected profit for the alternative if they have never experienced it or the average for the group as a whole? My recommendation it to connect your assumptions to the existing literature and the terminology that they use. Right now, your work is in some sense in the same vein as these and other papers, but no one would know this via a google or key terms search, as you do not cite the key papers or use their terminology.**

**Response:** We thank the reviewer for this comment. We acknowledge that, in practice, it is difficult to assess how growers will make decisions as there will be many different factors, which are also likely to vary depending on the setting or characteristics of the grower. We also note that we are not attempting to validate or replicate previous work on grower behaviour, but instead want to create a simpler, more flexible model of behaviour that allows use to test different hypotheses with relative ease.

**Action:** We have added a paragraph to the discussion (Lines 552 - 569) addressing how growers will access information. In this we describe how social networks vary between growers, but also how the growers’ use of different information sources will impact their trust and subsequent participation in control.

**Lastly, I am not a fan of the “catch phrase” used in the title. I am not against the catch phrase, but I had to spend some time trying to figure out what exactly it was saying. It reminded me of an Escher sentence or similar. I also am not a fan of the use of the colloquial “ain’t”. I have no recommendation – if you want to use that title, do so, but I thought it best to at least give you my honest feedback about it.**

**Response:** We thank the reviewer for their honest feedback on our title.

**Action:** We have changed the title to “How grower decision-making is modelled impacts the effect of plant disease control.**”**

**I have not gone into any of the more detailed comments, but leave such comments for later versions once the paper is revised. Overall, I believe that there is a publishable paper buried in this work, but the authors have to stop trying to put everything they have done into one paper, focus on communicating their main point, and dropping material that does not contribute to making this point.**

**Response:** We thank the reviewer for this and the other helpful comments that they have provided. We have undertaken extensive restructuring of the paper in order to simplify it and believe it to be much improved.

**Action:** None requested.

*Reviewer 3*

**Review of PCOMPBIOL-D-21-02295, “It ain't what you do, it's what drives why you do it: how the basis of growers' decision making affects the effectiveness of disease control”**

**General appraisal**

**This is a novel, sound and scientifically valuable paper which adds to our understanding of how the dynamics of growers’ behaviour impacts the management of plant diseases with a particular focus on CBSD. The model explanation and development are very clear and enjoyable to read, but the organisation of the paper is not very good. There are 4 different “models” that the author explores, with focus on one of them. However, it is often difficult to know which one the author is talking about, especially when discussing the results.**

**Response:** We thank the reviewer for this appraisal for the manuscript, and we are pleased that the reviewer has enjoyed reading parts of it. We apologise for the lack of clarity when discussing the different models and for the organisation of the paper.

**Action:** We have undertaken major restructuring of the paper, primarily focussed on the following:

- We have abbreviated the primary questions of investigation.
- We have restructured the Methods section so that the “Parametrisation” section is now Section 2.2.
- We have relegated all methodological details of – and results from – the spatial-stochastic version of our model from the main text to a new appendix (Appendix 2).
- We have abbreviated our results (moving much of the results relating to the “strategy vs.” models) to Appendices 1, 2 and 3 (including most of the mathematical detail).
- We have re-structured the Results section such that they follow directly from the questions posited in the introduction.

**General comments**

**In this manuscript the authors develop a model to understand how the growers’ decision to use Cassava Clean Seed Systems (CSS) for disease control, affects the dynamics of the spread of Cassava Brown Streak Disease (CBSD). To model the behaviour of farmers, the authors use game theory to model 4 different types of scenarios that a grower can follow to maximise their profit when considering the adoption of CSS. For the dynamics of CBSD spread they use a process based deterministic formulation that is then extended to a spatial and stochastic model.**

**The paper is enjoyable to read in some parts and the model is very well explained and easy to follow. However, the organisation of the different sections and presentation of the results is not well organised. This really needs a revision to make the document flow. I made some comments in sections where it was clear to me it needed to change, but please carefully review the document and make sure it flows.**

**Response:** We again thank the reviewer for this appraisal, and agree that there were several elements that needed to be restructured to allow to reader to follow our arguments and interpret our results.

**Action:** The changes we have made have been detailed in the response to the previous comment. In particular, we have undertaken a major restructuring of the results section such that it directly corresponds to each of the three questions we pose in the introduction. We have also moved much of the mathematical detail and detail of the spatial-stochastic model to the appendix, to again allow us to focus on the main results.

**The figures also need careful revision. Often the caption is lacking all the elements needed to fully understand the figure and the reader needs to keep on navigating through the text to understand it. Make sure that all the parameters used in the figures are explained and that the legends are large enough to read.**

**Response:** We apologise to the reviewer for the unclear presentation of our figures, and agree that the captions in particular often lack information.

**Action:** We have revised each figure and its corresponding caption to ensure that it contains all the relevant information needed. We have also removed any unnecessary detail from the captions and increased the size of the figure legends.

**Finally, I think the authors need to re-focus the questions they explore (lines 92-98) so the results follow from that and the paper impact is clearer.**

**Response:** We thank the author for highlighting this, and agree that we were originally focussing on too many questions.

**Action:** Lines 125 – 133: We have narrowed our focus to three questions of interest: Lines 116 – 128: In light of this comment, we have reduced our focus to three questions: (1) How does the formulation of the comparison (i.e. ``strategy vs." or ``grower vs.") affect the model and its results? (2) How does grower participation in the CSS depend on epidemiologically and economically important parameters, and how can high levels of control be encouraged? (3) How does systemic uncertainty in epidemiological parameters affect participation in the CSS?

**There are some very interesting results that get lost among the text which could be highlighted from the beginning in the abstract and also in the discussion. As such, the discussion needs to re-focus and really show the most interesting results found throughout the text.**

**Response:** We thank the reviewer for this comment, and agree that the discussion needed to be re-focused, particularly in response to many of the previous changes we have outlined.

**Action:** We have undertaken extensive restructuring of the discussion. It now follows the ordering of the Results Section, focusing on both a description and contextualisation of our primary results. Discussion of other results (in particular the spatial-stochastic model) has been moved to Appendix 2.

**Nonetheless, I think this is a very interesting paper with novel approaches and non-intuitive results that the authors nicely linked to their counterparts in human epidemiology. Reorganising the flow, carefully curating the figures and making sure of defining all parameters and variables would make this a much better manuscript.**

**Response:** We thank the reviewer for this positive appraisal. We hope that in our extensive restructuring of the manuscript we have improved its flow and readability.

**Action:** Addressed above in previous (and subsequent) comments.

**Specific comments**

1. **It would be great if the authors could give examples of systems where each grower strategy may happen. For example, in the case grower vs. population, I can think of an organic farmer that has a larger profit due to the “premium” that the organic label confers them. In a region where spraying for disease control is the norm, they may choose to reject spraying to keep their higher profit in comparison to the profit of the spraying population.**

**Response:** We thank the reviewer for this interesting suggestion, which will certainly help to ground our theoretical work.

**Action:** Line 91 – 103: We have added a paragraph beginning “Hypothetical scenarios in which each of these model types can be constructed...”, which details examples of when each of our model formulations may be used in a real-world setting.

2. **Detailed comments are provided in an annotated PDF version of the submitted manuscript.**

*To aid the Editor and Reviewer #3 in understanding our responses, we have extracted the comments from the marked-up PDF provided by the Reviewer and summarised them here before describing which changes we have made in response.*

***Short title:***

**I would suggest deleting either the word control or the word management as adding both is redundant**

**Response:** We thank the reviewer for this observation.

**Action:** We have amended the title to: “Human behaviour and plant disease management”.

***Abstract:***

**Controlling → driving**

**Response:** We agree with the reviewer that, given we talk of disease control throughout the abstract, “driving: is a better verb to use in this context.

**Action:** Line 9: We have changed the wording to: “biological factors driving transmission”

**Remove “epidemics clearly”**

**Response:** We agree with the reviewer that this rewording.

**Action:** Line 9: We have changed the sentence as follows: “While the spread of plant disease depends strongly on biological factors driving transmission, it also has a human dimension.”

**“Benefit” → “perceived profit increase due to better disease management”**

**Response:** We thank the reviewer for this suggestion, which will make the language more precise

**Action:** Line 12 – 14: We have changed the sentence as follows: “we model how the perceived increase in profit due to disease management influences participation in clean seed systems (CSS)”.

**“Additionally, though the CSS can reduce the burden of disease, and allow a scenario in which all growers control, disease is not eliminated from the system.**” **→ "Additionally, even thought good disease management can be achieved through the implementation of CSS, the disease is not eliminated from the system"**

**Response:** We thank the reviewer for this suggestion. However, we believe that the possibility of an “all control” equilibrium is a key result from our model, and thus should be included in the abstract.

**Action:** Lines 19-21: We have amended the sentence to read: “Additionally, even though good disease management can be achieved through the implementation of CSS, and a scenario where all controllers use the CSS is achievable, CBSD is rarely eliminated from the system.”

**Match up short titles**

**Response:** We thank the reviewer for highlighting this inconsistency.

**Action:** We have amended the short title to: Human behaviour and plant disease management.

***Introduction:***

**Line 28:** **only 4 references for "widely considered" and all of them on game theory? There are many other approaches as well e.g. cellular automata, agent based models, opinion dynamics, physics inspired (e.g. Sznajd and Ising models) that you did not mention.**

**Response:** We agree that, at this point in the manuscript, we should introduce the reader to a broader range of models and body of previous work.

**Action:** Lines 28: We have added six extra references at this point, including two reviews. The referenced models now range from those using game theory to agent-based models that account for risk aversion of growers.

**Line 29: this is a strong sentence, can you back it up? Is this really true? Consider rewording or simply saying that you will follow a game-theoretical approach.**

**Response:** We agree that this statement is strong, though acknowledge that it is difficult to show.

**Action:** Line 30-31: We have re-worded the sentence to the following: “Where studies do consider individual's decisions there is much variation in how decision-making is modelled, although it clearly will affect the outcomes of their decisions.”

**Line 30: likely → unlikely**

**Response:** We find it interesting that the reviewer believes that increased disease will make control uptake more likely. However, given our later findings – that above a certain threshold of disease incidence, participation in control is unlikely – we agree that the increased use of control with increased disease incidence is not guaranteed.

**Action:** Line 47 – 48: We have amended the sentence as follows: “which may make disease control more likely”, and have added the following: “Indeed, perceived risk of other growers benefiting from an individual's control policies are a prominent reason why growers do not participate in control schemes ([18],[23]).”

**Line 48: thus can be modelled using game theory analysis (as it is not the only way to do it)**

**Response:** We agree with the reviewer that the current wording gives the impression that the only way to analyse such as system would be by using game theory, which was not our intention.

**Action:** Line 49. We have changed the sentence to read: “The strategic nature of these behaviours can be modelled using game-theoretic analysis.”

**Lines 48 – 52: delete (unnecessary paragraph...no need to define what game theory is)**

**Response:** Whilst we acknowledge that many readers will be familiar with game theory and its applications, we believe that having at least a short introduction to the field will be useful for those that do not and will therefore help them to understand the subsequent models presented in the paper.

**Action:** Line 49-61: We have shortened the paragraph, though not removed it entirely.

**Lines 54 – 55: unnecessary, just leave the references**

**Response:** We agree with the reviewer that contextualising the references is unnecessary

**Action:** Line 63: We have deleted “both use replicator-dynamic inspired approaches to model vaccination uptake”.

**Line 63: As you are introducing game-theory models in the previous paragraph, this would read as if [5] and [6] are also game-theoretical models but 6 is not. Please clarify this paragraph.**

**Response:** We apologise for this lack of clarity and have changed the text to reflect this observation.

**Action:** Line 71: We have changed the text to read: “An alternative means of assessing profitability, is used in [5], [6] and [10] (though the inclusion of grower characteristics in [6]} means it deviates from pure game-theoretic assumptions of rationality).”

**Move line 68 to line 72 after [6].**

**Response:** We respectfully disagree with the reviewer here, and argue that as line 72 discusses the lack of comparison between the two proposed “grower vs.” models (“grower vs. population” and “grower vs. alternative”), it should come after the discussion of both model types (on line 67).

**Action:** Line 80: We have not changed the ordering of the sentences but do note that much of the surrounding text has changed, which will help to clarify our meaning.

**Line 69: Can you give examples of scenarios where farmers may use each one of these 4 behaviour strategies?**

**Response:** We thank the reviewer for this suggestion and agree that having hypothetical examples of when growers may use each of these strategies will help the reader understand the different model formulations.

**Action:** Line 91 – 103: We have added a paragraph beginning “Hypothetical scenarios in which each of these model types can be constructed...”, which details examples of when each of our model formulations may be used in a real-world setting.

**Line 69: delete “to date in publication.”**

**Response:** We are happy to agree with the reviewer that this clarification is unnecessary.

**Action:** Line 82: We have deleted “to date in publication.”

**Line 88: Delete “making the system amenable to game theoretic analysis.”**

**Response:** We thank the reviewer for this suggestion to make our text more concise.

**Action:** Line 116: We have deleted “making the system amenable to game theoretic analysis.”

**Line 95: very unclear question/objective? what results? from 1), from 2), from both? what quantities?** **Line 98: all these questions? Focus on the most relevant results you would like to emphasise. The main question (as I understand) you would like to address is how, when and under what circumstances does a grower decide to adopt a control strategy to maximise their profit. For that, you consider epidemiological and economic parameters through a series of investigations/models**

**Response:** We thank the reviewer for these comments, and have chosen to address both together as they relate to the questions we aimed to address with this piece of work. We note that a similar concern was highlighted across all reviews. We acknowledge that in this paper, we were trying to address too much and therefore our main messages (relating to model formulations and the effect of parameters/uncertainty) were lost.

**Action:** Line 125 – 129: We have now consolidated our investigation to three questions: (1) How does the formulation of the comparison (i.e. ``strategy vs." or ``grower vs.") affect the model and its results? (2) How does grower participation in the CSS depend on epidemiologically and economically important parameters, and how can high levels of control be encouraged? (3) How does systemic uncertainty in epidemiological parameters affect participation in the CSS? We have moved our investigation of spatial and stochastic effects to a new appendix (Appendix 3).

***Materials and Methods:***

**Line 106: big assumption!**

**Response:** We acknowledge that this is a simplifying assumption and that, in practice, growers may have cassava from a variety of sources (clean vs replanted, for example) growing in their field. However, as we are not modelling within-field dynamics, we believe this simplification is justified.

**Action:** Line 142-143: We have qualified our simplifying assumption: “… and make the simplifying assumption that controllers plant only certified virus-free material, thereby ignoring the possibility of an individual grower cultivating cassava from a variety of sources”.

**Table 1: I might have missed it, but I don't see anywhere where you explain why did you chose these values. In any case, it would be nice to be able to locate this easily for readability**

**Response:** We apologise to the reviewer for this lack of clarity, and agree that introducing parameterisation earlier in the methods would be easier to follow.

**Action:** Line 162: We have moved the “Parameterisation” subsection of the methods to be Section 2.2, which follows directly after the first introduction of the parameter table.

**Line 142: If the grower can estimate this probability with perfect accuracy, they would know exactly what to do next year, as they would be able to predict which strategy is more profitable at all times. Maybe perceived instantaneous probability of vertical transmission is better?**

**Response**: Whilst it is true that for vertical transmission will only the instantaneous probability matters, for horizontal transmission the probability of infection at time of planting is only an approximation for the entire growing season. Thus, as growers are basing their decisions on the instantaneous probabilities of infection they therefore cannot know with certainty which strategy will remain profitable.

**Action:** NA

**Line 142: Earlier you said "relatively precisely" which one is it?**

**Response:** We thank the reviewer for highlighting this inconsistency.

**Action:** Line 198: We have deleted “relatively”.

**General: Write a single equation number (same goes for 20-21 and 22-24) and maybe define them in the text**

**Response:** We agree with the reviewer that only labelling the final mathematical expressions, rather than the word equations or their “intermediate” forms, reduces the overall number of equations and adds clarity for the reader.

**Action:** We have removed the equation numbering from all word equations and defined the variables described by the equations in the text.

**Line 152: , P_C**

**Response:** We thank the reviewer this suggestion, which will clarify the meaning of the equation for the reader.

**Action:** Line 215: We have amended the sentence to read: “the estimated expected profit for a controller, *P_C*, would be’

**Line 155: or estimate?**

**Response:** We thank the reviewer for this suggestion, which makes our language more consistent.

**Action:** Line 218-220: We have rephrased this sentence to be: “Calculating $P$ therefore requires growers to know the instantaneous proportion of the population controlling. This calculation of $P$ is instantaneous, i.e. based on the state of the system at the time of planting, and provides an estimate of $P$ for the next season.”

**Equation 26: Capital C**

**Response:** We thank the reviewer for spotting this typo.

**Action:** Equation 21: We have changed the subscript from “c” to “C”

**Line 159: again, what does this mean? You have to define these parameters before you put them into an equation and explain what they are The section that goes from lines 164-179 may be better at the beginning of 2.5 (assuming that you define \Delta and \nu before equation 33)**

**Response:** As both of these comments relate to the structure of the paper and how we introduce parameters, we have chosen to address them together. We agree that describing how Δ (the expected difference in profit) is estimated should come before the description of the switching terms (whose forms depend on Δ). We have also added general descriptions of the formulation for each comparison.

**Action:** We have moved lines 164-170 (now lines 214-228) to the beginning of the section on behavioural models (now Section 2.6).

**Equation 33: You have not defined \nu yet. I can see from table 1 that it is the "responsiveness of farmers", but what does this mean anyway, and what does it mean that it is 10?**

**Line 162: what does this mean? This becomes clearer as you read along, but it is really disconcerting the first time you read it. It would be better to define or explain it beforehand.**

**Response:** As both of these comments relate to the definition of η, we have chosen to address them together here. We acknowledge that a constant theme throughout these comments was uncertainty over the definitions of parameters and their values. We have since restructured the paper such that the section on parameterisation is now Section 2.2, and thus precedes all model development.

**Action:** We have changed the structure such that the section on parameterisation is now Section 2.2, and precedes the derivation of the switching terms/behavioural model. We have also added a definition of η at Line 229 to remind the reader of its meaning (the responsiveness of growers).

**Equation 47: again, no definition of these two new parameters in the text.**

**q_\beta represents the perceived value of the rate of horizontal transmission and q_I is the perceived number of infected fields**

**Response:** We agree with the reviewer that adding definitions of these parameters would assist the reader, and omitting the definitions was an oversight.

**Action:** Line 275-277: We have added definitions for both q_β and q_I to the main text before the relevant equations.

**Line 231: what happened here? I think 2.8 should be 2.7.1? and so on up until 2.12?**

**Response:** We thank the reviewer for highlighting this inconsistency in formatting.

**Action:** We have amended the labelling of the subsections such that Sections 2.8 to 2.12 are now subsections of Section 2.7.

**Line 266: How does this work? What is a Bernoulli trial? That is not common knowledge I would think Could find a citation? Otherwise seems laborious to describe a Bernouilli Trial**

**Response:** We thank the reviewer for highlighting this oversight.

**Action:** We have added a reference that explains Bernoulli trials. We note that this section is now in Appendix 1.

**Equations 51 and 60: italics**

**Response:** We thank the reviewer for spotting this typo

**Action:** We have corrected the mistakes and italicised the correct elements of the equation (which has now moved to Appendix 3 Line 903).

**Line 275: This section may be better located before the model explanation, this way parameters such as \nu become clearer from the beginning. Maybe just after line 117**

**Response:** We agree with the reviewer that the structure in the m/s as originally submitted relied on the reader reading ahead to understand parameters.

**Action:** We have changed the structure such that the section on parameterisation is now Section 2.2 and precedes the derivation of the switching terms/behavioural model.

**Also maybe separate the non-spatial and the spatial model?**

**Response:** We thank the reviewer for this comment, and as it feeds into a larger concern amongst reviewers about the structure/length of the paper, we have decided to separate the two forms of the model (non-spatial and spatial) entirely, and to focus entirely on the non-spatial model in the main text

**Action:** Parameterisation for the spatial model has been moved to Appendix 3.

***Results:***

**Line 311: Maybe here you could better organise the sections to make it more readable e.g. write 3.1.1 as strategy vs. population model then write in bold "Probability of infection..."**

**Response:** We thank the reviewer for this suggestion, and note that it follows a wider thread of comments across reviews about the structuring of results.

**Action:** We have now altered the Results section such that the results fall under three headings, each relating to one of the questions outlined in the introduction. We have also modified the results such that only those relating to the first question (concerning the comparison between model formulations) looks across all three models; the rest solely focus on the “grower vs. alternative” model. We note that this (and other suggested changes to the results section) now appear in the Appendix.

**Line 319: change “due to the increased vertical transmission” to “as field infection has already taken place through vertical transmission”**

**Response:** We thank the reviewer for suggesting this rephrasing and agree that it will clarify our explanation for the reader.

**Action:** We have changed the explanation “due to the increased vertical transmission” to “decreases as field infection has already taken place through vertical transmission”. We note that this result has been moved to Appendix 3 (Line 1105).

**Line 328: “should not switch strategy” → “switching strategy”**

**Response:** We agree with the reviewer that this sentence needed to be rephrased to remove any attribution of human traits to the mathematical constraints in this model.

**Action:** We have rephrased the sentence to “cannot switch strategy”. We note that this result has been moved to Appendix 3 Line 1114.

**Line 332: Be specific. The results shown are for "strategy vs. population" model. By making it general it gets confusing as you are not showing results for "strategy vs. alternative" in this instance.**

**Response:** We agree with the reviewer that, though this result holds for the “strategy vs. alternative” model, as the figure refers only to the “strategy vs. population” model we should be more specific in our description.

**Action:** We have now clarified our description to specifically refer to the “strategy vs. population” model and added the following sentence after the description: “This also holds true for the ``strategy vs. alternative" model.” We note that this result has been moved to Appendix 3 Line 1120 – 1121.

**Lines 334-338: Although interesting, it is not relevant here as you are discussing the strategy vs. models.**

**Response:** We agree with the reviewer that this point of the manuscript is not the most effective place to discuss the effect of η on the “grower vs.” models.

**Action:** We have moved the text describing the effect of η on the “grower vs.” models to the Appendix 2 Line 1063 – 1066.

**Line 341: again, be specific: add “vs. population”, “vs. alternative” and “respectively”**

**Response:** We agree with the reviewer that specifying which sub-panel refers to each model will clarify our results for the reader.

**Action:** We have changed the reference to: “strategy vs. population" and “strategy vs. alternative" models (Figure 3(A)-(B) respectively).

**Figure 2: Figures should be understandable on their own. You have to go into the text to follow what each parameter means. Define those in the figure caption please. Add q_N and definition of ϕ**

**Response:** We thank the reviewer for this comment, and agree that adding definitions of each parameter would ease interpretation for the reader.

**Action:** We have added definitions of ϕ (the cost of control) and the probabilities of infection to this caption. (Note: this figure has now been moved to Appendix 1.)

**Delete Note, however, for the “grower vs.” models, the value of η does affect the final equilibrium values (see Appendix 2). Also, please make legends bigger so they are readable.**

**Response:** We agree with the reviewer that confining discussion of the effect of parameters on alternative strategies should be confined to the main text:

**Action:** We have removed “Note, however…” from the figure legend of Figure 2 (which is now Appendix 1 Figure 1). We have also increased the size of the figure legends across all figures.

**Figure 3: 1. Legend font of the figure is super tiny you can barely read it. Make it bigger.**

**Response:** Thank you for suggesting this improvement to the graph.

**Action:** We have now remade all of our figures to have larger figure legends for clarity.

**2. You did not explain what each line represent. I can assume S_N is population of susceptible no controllers, but you have to make it explicit. Also, what does the green line (control) represents? Is this the green line in C? How do you get this one? I assume is the sum of Infected and Susceptible controllers?**

**Response:** We agree with the reviewer that adding clarification of what each line represents on the graph will make it easier for readers to interpret results.

**Action:** We have added the following to the figure legend (of what is now Figure 3): Here, S_N are susceptible non-controllers, I_N infected non-controllers, S_C are susceptible controllers and I_C infected controllers. C represents S_C + I_C.

**you mean C (instead of B)?**

**Response:** Thank you for spotting this typo.

**Action:** Figure 3: We have changed the reference in the figure legend to sub-panel C instead of sub-panel B.

**Line 361: We need a section number or at the very least a new paragraph here. Line 404: new section**

**Response:** We thank the reviewer for suggesting this structural change, as it will help the reader understand to which model we refer. As both comments refer to similar points, we have address these comments together.

**Action:** We have moved these results to Appendix 3 and integrated them in Lines 988 – 1047.

**Line 362: what parameters?**

**Response:** We thank the reviewer for highlighting this ambiguity in the text.

**Action:** Figure 2: We have added the following text: “Effect of the rate of horizontal transmission ($\beta$) and the cost of control ($\phi$) on final equilibrium attained.”

**Figure 4: The text above your figure 4a comes out of the figure frame.**

**Response:** We thank the reviewer for pointing out this issue in our figure.

**Action:** We have remade this figure (which is now Figure 2) to correct the issue.

**what does DFE stands for?** **what are the parameters used in this figure? are they the ones found in Table 1? You could not reproduce this figure without this knowledge**

**Response:** We thank the reviewer for their observations on this figure, and agree that adding more to the figure legend will assist the reader. We have also fixed the formatting issue.

**Action:** Figure 2: We have added the following text: “Parameters are as in Table 1” and “Below $R_0 = 1$, only the disease-free equilibrium (DFE) is possible.”

**why do we have both "strategy vs." as title in this figure? do they reach the same equilibria? why is it different in the caption then? Be specific.**

**Response:** As we have explained in the main text, despite the fact that they are using different quantities of comparison (population averages vs. strategy averages), both of these models have the same equilibria (because at equilibrium, the strategy profits must be equal). Thus, rather than repeating a graph, we have included two headings on the same graph.

**Action:** Figure 2: For clarity, in the legend we have added “strategy vs. alternative” to the explanation in the caption.

**Line 422: awkwardly written sentence.**

**Response:** We apologise to the reviewer for this lack of clarity.

**Action:** We have rephrased the sentence as follows: “The non-zero value of the loss due to disease (*L*) means that the conclusion presented in Equation App. 56 is impossible. Therefore, for a “no-control” equilibrium to be possible, all fields must be infected (i.e. N = I_N and q_N = 1).” We note that this has been moved to Appendix 3 Lines 1095 – 1099.

**why don't you show this instead? you cannot see this result, so don't mention it here. Line 424: so don't mention it in the caption figure. In the figure mention only what you can see**

**Response:** As both of these comments refer to Figure 4 (B) (which is now Figure 2 (B)), we address both of them here. We agree with the reviewer that it is confusing to refer to a result that is not shown in the figure here, however we believe that it is important to highlight that the equilibria shown in Figure 4 (B) for the “grower vs. alternative” comparison are not the only possible equilibria for the model.

**Action:** We have abbreviated our discussion of the different possible equilibria in (B), making reference to the corresponding figure in Appendix 2, and confine discussion of what leads to the different equilibria to the main text.

**Figure 5: Change sub-title to “Control uptake through CSS dependent on epidemiological and economic parameters”.**

**Response:** We thank the reviewer for this suggestion to clarify the title of our figure.

**Action:** Figure 4: We have changed the figure to show the effect of the parameters on both the uptake of control and the proportion of infected fields, and have thus changed the subtitle to: “CSS participation and proportion of infected fields dependent on economic and epidemiological parameters.”

**Sub figure titles come out of the whole figure and legend of what the contours show is very small, you really need to look for it to know what the contours tell.**

**Response:** We agree with the reviewer that the legend on the figure was small and thus it was difficult to discern differences between graphs.

**Action:** Figure 4: We have re-made the figure with larger figure legends and titles.

**This is not what I am seeing. For small values of \beta, there is no point in controlling (presumably because there is very little risk of horizontal transmission?) For slightly larger values of \beta, when horizontal transmission becomes significant? control becomes more relevant, especially for small values of \phi where the cost of control does not impact severely the growers' profit. As cost of control increases, the incentive to embrace CSS diminishes.**

**Response:** We thank the reviewer for highlighting our inaccurate language, as we should have referred to “medium-to-low” values of β. The reviewer is correct, in that very low values of β render control useless, as there is no disease in the system to protect against.

**Action:** Note, this figure has been reformulated and the sub-panel to which the reviewer refers is now in Appendix 4 Figure 4. We have clarified in the text what we meant by “low values” of β, which should have been “medium-to-low” values. We have added the following text: *At low values of $\beta$, no-one should control as the probability of infection is sufficiently low that it is not necessary. As $\beta$ increases, controlling is more beneficial and the cost of control is perceived to be worthwhile. However, as infection becomes more likely, the value of control diminishes so it is not worthwhile to invest in control.*

**what does these figues tell you?**

**Response:** This figure shows, in the first case, what a counter-intuitive response (that increased rate of horizontal transmission can decrease control). Also, it shows that the impact of parameters really depends on the model set-up.

**Action:** We have clarified the text but also reformulated the figure for clarity (see above comment).

**Line 425: Responses to what? No introduction to what you are doing here**

**Response:** We thank the reviewer for highlighting this unclear statement.

**Action:** Line 378: We have added “We investigate the change in uptake of control and proportion of disease fields with when the cost of control (ϕ) , probability of vertical transmission (*p*) and loss due to disease (*L*) are varied (Figure 4). We have also amended the sentence to “Responses to changes in parameter values…” .

**Line 428: why not? this is quite relevant result for what you are doing here**

**Response:** We agree with the reviewer that these results are important, though we have focussed on a subset of parameter scans that are illustrative of broader patterns. However, we agree that examining the effect of the loss due to disease (*L*) on parameter values would be valuable for readers.

**Action:** We have included a further parameter scan in Figure 4 (C) and (F) that shows a parameter scan over the rate of horizontal transmission (β) and the loss due to disease (*L*).

**Line 432: this is not what you are showing, as by keeping cost of control low, the CCS adoption is still quite high (from the figure). You need to work in this interpretation of results**

**Response:** We thank the reviewer for pointing out our lack of clarity over this result. We should have emphasised that changing the value of β (rate of horizontal transmission) narrowed the range of values of ϕ (cost of control) for which growers would use the CSS. This has now been corrected.

**Action:** “When β increased, the higher probability of infection means that control is too costly” was changed to “When β increased, the higher probability of infection narrows the range of costs for which a controller will consider participation in the CSS*”* (Line 385)*.* We note that for all models but the “grower vs. alternative” model, this has now been moved to Appendix 4.

**Line 437: maybe you can show this here in another set of 3 subfigures below?**

**Response:** We agree with the reviewer that showing the effect of subsidy on the uptake of control is an interesting result, though we have decided to confine these results to the Appendix to better focus our messaging to the three primary questions. Additionally, it is clear from panel (A) that lowering the cost of control encourages participation in the CSS.

**Action:** We have added a paragraph that summarises the implications of our parameters for encouraging participation in control. In that, we emphasise the benefit of a lower cost of control (which could be a subsidy) and again direct the reader towards Appendix 4.

**Line 438: This is very relevant, why not inculde it in main text?**

**Response:** We thank the reviewer for this suggestion and agree that adding the effect on yield and profit is an important result.

**Action:** We have modified the previous figure from the appendix to now only consider the “grower vs. alternative” strategy and added it to the main text. Additionally, we have included more subpanels on how the proportion of infected fields changes with parameter values to the (new) Figure 4.

**Line 448: worth mentioning what \nu_\beta and \nu_I mean and why did you choose those values**

**Response:** Thank you for this suggestion, which will certainly assist the reading of the text.

**Action:** Lines 412 – 414 and Lines 423: We have added definitions of both of these parameters and clarified that the range of values chosen was to allow for under- and over-estimation of the parameters.

**End of page 34: what happened here?**

**Response:** We thank the reviewer for highlighting this formatting issue, which was caused by the figure and legend being too large.

**Action:** Figure 6: It has been resolved by removing the panels with the subsidy ((C) and (D)), which has been de-emphasised throughout the text.

**Figure 7: spatial-stochastic**

**Response:** Thank you for highlighting this grammatical error.

**Action:** This error has been rectified throughout the text.

**Figure 8: you mean Figure 8A shows the proportion of controllers over time (seasons) for the spatial-stochastic model while Figure 8C shows the proportion of growers dependent on the rate of horizontal transmission for the deterministic model. of what? no good explanation here** **B is for the spatial-stochastic.**

**Response:** We thank the reviewer for noticing this inconsistency and inaccuracy of language.

**Action:** Appendix 2 Figure 8: We have modified the legend for the appropriate figure to be: (A) and (C) The proportion of controllers and infected fields after 50 seasons for the spatial-stochastic model and (B) and (D) the equilibrium values of control ($S_C + I_C$) and infection ($I_N + I_C$) for the deterministic model.

**What do they say? You mention in the text, but don't say anything here**.

**Response:** We agree with the reviewer that we should emphasise the main result from this figure in the legend.

**Action:** Appendix 2 Figure 8: We have added the following text: “The results in (A) and (C) closely align with the equilibrium values in the non-spatial model ((B) and (D)), indicating that our results are robust to spatial and stochastic effects.”

**This do not belong in the figure, maybe in the text, but not in the figure.**

**Response:** We thank the reviewer for this comment, and agree that details of extinction events should be discussed in the main text.

**Action:** We have moved discussion of extinction events to the main text (Lines 919-921).

***Discussion:***

**Line 497: Surprisingly few plant disease studies examine how behaviour alters the dynamics of epidemic models. Here we presented a model that integrates grower decision making into a simple model for CBSDand investigated the effect of economically- and epidemiologically- important parameters on disease spread. - > Surprisingly, few plant disease studies examine how human behaviour alters the dynamics of epidemic models. Here we presented a model that integrates game theory to simulate grower decision into a model for CBSD...**

**Response:** We thank the reviewer for this suggestion, which improves the accuracy of our language and what we aimed to describe.

**Action:** Line 436 – 437: We have changed the sentence to: “Surprisingly, few plant disease studies examine how human behaviour alters the dynamics of epidemic models. Here we presented a model that integrates game theory to simulate grower decision-making into a model for CBSD”.

**Line 501: If you read this in isolation it doesn't say anything. Here you write your most interesting findings.	Line 503: delete (Not needed here, but it could go into the "strategy vs." results)	Line 506: I am not sure what are you trying to say here. Clarify please. I changed the sentence, but I don't know if that is what you mean	Line 507: Delete therefore. And replace with “We have found that”	You could start here just after line 500, then talk about your results**

**Response:** As these four comments relate to the opening paragraph of the introduction, we have chosen to address them all together here. We thank the reviewer for their detailed comments and suggestions on this section of the manuscript, and agree that much of this section is simple repetition of the previous section (Results).

**Action:** We have changed lines 446-454 to read as follows: “We found that basing decisions on rational (the “strategy vs.” models) or strategic-adaptive (“grower vs.” models) changed the potential outcomes for the model. Only the ``strategy vs.” comparisons permitted an ``all control” equilibrium, whereas the low payoff for controllers with infected fields (the ``sucker's payoff") means that they will always have a non-zero probability of switching strategy in the ``grower vs.” comparisons. This difference in possible equilibria from the models shows that it is important not only to include behaviour in epidemic models, but to consider how it is included based on what quantities are being compared and what information will be available to individual growers, such as the disease pressure, profit of other growers and breakdown of the expected profit for each strategy.”

We have also significantly restructured the Discussion section such that it follows the new narrative of the paper, focussing on the three questions outlined in the Introduction. We have also added a summary of the main results to the first paragraph of the Discussion.

**Line 509: For example… Wants an example of how information impacts decisions? Like whether they can compare against the population or alternative? Or from life?**

**Response:** We thank the reviewer for suggestion the inclusion of an example here, which will emphasise the point we are making.

**Action:** Line 453: We have referred back to our results: “such as the disease pressure, profit of other growers or exact rate of horizontal transmission.”

**Line 520: This is quite interesting! and very nice analogy**

**Response:** We thank the reviewer for this positive comment.

**Action:** None requested

**Line 525: New paragraph**

**Response:** We agree with the reviewer that separating the paragraph at this point (where we start discussing the “all control” equilibrium” will emphasise this result. However, we note that in the restructuring this paragraph has been incorporated into a separate part of the text.

**Action:** We have included the message of this paragraph Lines 446 – 454.

**Line 527: what does this say?**

**Response:** We agree with the reviewer we need to contextualise this result (that the all-control equilibria are not possible for the “grower vs.” models) rather than simply re-stating it.

**Action:** Line 450: We have now added the following clarification: “This difference in the number of possible equilibria from the models highlights not only that including behaviour is important, but also that its means of inclusion (i.e. the way in which profitability is assessed) can restrict the potential range of outcomes predicted by the model.”

**Line 528: Add “In the strategy vs. model”**

**Response:** The reviewer is correct that this specific equation refers only to the “strategy vs.” models. However, we have now simplified this discussion and no longer refer to the equation in the discussion.

**Action:** Line 478: We have clarified the sentence to read: “In the ``strategy vs." models, we found that use of CSS could reduce CBSD spread”. On lines 484 – 489 we have also clarified that the result holds true for the other model types also, though cannot be proven analytically.

**Line 554: big assumption and usually not true**

**Response:** We agree with the reviewer that this is a big assumption and would not hold true in a real-world setting. We therefore dedicated the next several lines to discussing this limitation.

**Action:** Lines 501 – 512: We have dedicated the next several lines to discussing this limitation.

**Line 569: are they? Mmh**

**Response:** Line 489 – 499: We agree that, with our parameterisation, the value of the cost of control = 0.25 (as was obtained from the literature) does not currently account for these non-monetary costs. However, we argue that the general parameter of phi could account for costs outside of the direct cost of participation in the CSS.

**Action:** We have modified the text to read “could be encapsulated”.

**Line 573: and many other variables!!! e.g. weather, variety chosen, etc.**

**Response:** We thank the reviewer for highlighting this oversight, and agree that we should note the range of variables that will affect cassava yield.

**Action:** Line 572 – 573: We have added the following to the text: “(as well as many other factors, such as environmental stochasticity, which variety of cassava was planted, availability of fertiliser or pesticide etc., ([61], [62], [63]))”.

**Line 604: but not always!**

**Response:** We agree that use of the CSS does not always increase the yield, and thank the reviewer for pointing this out.

**Action:** Line 587: We have changed our assertion to: “has the potential to increase the yield of controllers”
